# Advances in nanomaterial-enhanced immunosensors for ultra-sensitive tumor marker detection: Enabling early cancer diagnostics

**DOI:** 10.3389/fchem.2026.1849931

**Published:** 2026-08-18

**Authors:** Shengbo Jin, Jun Yu, Yuxin Jiang, Mingzhu Li

**Affiliations:** 1 Liaoning University of Traditional Chinese Medicine, Shenyang, Liaoning, China; 2 Liaoning Cancer Hospital and Institute, Shenyang, Liaoning, China

**Keywords:** bioconjugation, cancer diagnosis, immunology, immunosensor, interdisciplinary technology, nanomaterials, nanotechnology, tumor marker

## Abstract

Tumor markers are critical for early cancer diagnosis and directly influence treatment outcomes and patient survival. Although enzyme-linked immunosorbent assay (ELISA) and radioimmunoassay (RIA) show satisfactory selectivity in clinical applications, their limited sensitivity for low-abundance biomarkers, relatively long assay time, and reproducibility issues have promoted the development of advanced immunosensing platforms. Recent progress in nanomaterial synthesis has improved immunosensor performance by enhancing antibody immobilization, electron transfer, catalytic activity, signal amplification, photoelectric conversion, and luminescence efficiency. This review summarizes nanomaterial-enhanced immunosensors for clinically relevant tumor biomarkers, including carcinoembryonic antigen (CEA), prostate-specific antigen (PSA), cancer antigen 153 (CA153), alpha-fetoprotein (AFP), cancer antigen 125 (CA125), cancer antigen 199 (CA199), and human epidermal growth factor receptor 2 (HER2). To reduce repetition and emphasize analytical performance rather than nominal material categories, this review adopts a platform-centered and mechanism-oriented framework. Electrochemical, photoelectrochemical, electrochemiluminescent, and chemiluminescent immunosensors are compared as major signal transduction platforms, with representative nanomaterials discussed according to their roles in antibody immobilization, electron-transfer acceleration, catalytic amplification, charge separation, luminescence regulation, magnetic enrichment, and interface stabilization. In addition, cross-platform comparisons of sensitivity, detection limit, specificity, recovery, assay time, storage stability, cost, scalability, and clinical applicability are provided to clarify the translational value of different sensing strategies. This comparative analysis highlights that clinical applicability is primarily determined by integrated platform performance, including sensitivity, stability, manufacturability, and compatibility with point-of-care testing, rather than by the use of a specific nanomaterial alone.

## 1 Introduction

As the second leading cause of global mortality, cancer accounts for nearly one-sixth of all deaths worldwide, with the International Agency for Research on Cancer reporting 9.7 million cancer-related deaths in 2022 and projecting this number could rise to 35 million by 2050 (Bray et al., 2024; Siegel et al., 2024). For decades, cancer research has focused on elucidating molecular differences between normal and neoplastic cells, while clinical evidence consistently underscores early diagnosis as the pivotal determinant for improving cure rates and reducing case fatality (Hajdu and Vadmal, 2013; Tang et al., 2017).

Current cancer diagnostic modalities encompass traditional techniques: medical imaging (CT/MRI/PET-CT), histopathological biopsy, endoscopic examination, and serum tumor marker assays; as well as emerging molecular diagnostic approaches including next-generation sequencing (NGS) and circulating tumor DNA (ctDNA) detection (Li et al., 2022; Li et al., 2024; Zhong Y. et al., 2021). Though histopathological biopsy remains the diagnostic gold standard for cancer confirmation, this method is inherently invasive and demonstrates suboptimal diagnostic sensitivity (Chen L. et al., 2020). Medical imaging and endoscopic examination can only detect millimeter-scale structural alterations, often leading to mid-to-late-stage diagnoses (Cao et al., 2023; Zaak et al., 2001). Molecular diagnostic techniques also exhibit certain limitations including high costs, technical complexity, and the requirement for integration with clinical context for comprehensive interpretation (Brunstein, 2016; Li et al., 2017); for example, detection of genetic mutations does not equate to disease manifestation, necessitating functional validation or longitudinal follow-up to confirm pathogenicity (Ho et al., 2024). Within this context, tumor markers—biologically active molecules secreted by tumor cells or produced through systemic host responses (including proteins, enzymes, hormones, and glycoproteins)—offer distinct advantages as their concentration fluctuations dynamically correlate with tumor burden and permit non-invasive monitoring via serological assays (Abele et al., 1999; Fiebiger and Wiltschke, 2001; Xing et al., 2024). Several clinically significant tumor biomarkers have already achieved widespread clinical application (Table 1). Despite these advantages, the clinical potential of tumor biomarkers remains hindered by the analytical limitations of conventional detection platforms.

**TABLE 1:** List of clinically significant tumor markers for common malignant tumors.

Tumor marker	Type of cancer	References
CEA	Colorectal cancer, ovarian cancer, lung cancer and other cancers	Beauchemin et al. (2013), Seale and Tkaczuk (2022), Shimada et al. (2014)
PSA	Prostate cancer	Stenman et al. (1999)
AFP	Liver cancer, germ cell tumors	Ahn et al. (2021), Galle et al. (2019)
CA153	Breast cancer	Han et al. (2021), Molina et al. (2005)
CA199	Pancreatic cancer, gallbladder cancer, bile duct cancer, stomach cancer	Klein (2021), Rawla et al. (2019), Wagle et al. (2025)
CA125	Ovarian cancer	Fawzy et al. (2016), Zhang et al. (2021)
HER2	Breast cancer, ovarian cancer, endometrial cancer, fallopian tube cancer, stomach cancer	Carvajal et al. (2018)

Compiled by the authors based on References.

Conventional detection technologies (ELISA and RIA) exhibit notable limitations in clinical diagnostics. While traditional assays fulfill basic diagnostic requirements (Azzouzi et al., 2022; Dai et al., 2019; Malati, 2007; Shen et al., 2022), ELISA demonstrates insufficient sensitivity in specific clinical scenarios: studies show that conventional ELISA fails to detect CA125 levels below 15 U/mL in post-surgical ovarian cancer patients, missing subtle elevations (5–15 U/mL) indicative of metastatic recurrence—a critical gap given that early recurrence detection significantly improves 5-year survival rates (Charkhchi et al., 2020; Wang et al., 2021). This limitation stems from protracted assay duration (4–6 h/assay) and labor-intensive procedures (≥12 manual steps, e.g., dilution/washing) (Henry and Hayes, 2006). Therefore, this necessitates alternative sensing paradigms capable of ultrasensitive biomarker quantification at extremely low concentrations to improve patient outcomes. The key differences between traditional methods and nanotechnology-based methods are detailed in Table 2.

**TABLE 2:** Comparison between traditional and nanotechnology-based methods.

Comparison item	Traditional methods	Nanotechnology-based methods	References
Detection principle	Antigen–antibody recognition with direct signal readout	Nanomaterial-assisted signal amplification combined with antigen–antibody recognition	Cao et al. (2023), Zaak et al. (2001), Brunstein (2016), Li et al. (2017), Ho et al. (2024), (Ahn et al. (2021) ; Galle et al. (2019) ; Stenman et al. (1999), Augustine et al. (2019) ; Chen et al. (2020b) ; Cui et al. (2007) ; Deng et al. (2020) ; Lilja et al. (2008) ; Lin et al. (2021) ; Liu et al. (2022) ; Ma et al. (2016) ; Nasrollahpour et al. (2023) ; Pathak et al. (2017) ; Qin et al. (2019) ; Ranjan et al. (2024) ; Ren et al. (2025) ; Shang et al. (2021) ; Shanmugam et al. (2015) ; Srivastava et al. (2018) ; Tao and Rouhi (2023) ; Wang et al. (2011) ; Wang et al. (2013a) ; Wang et al. (2022) ; Yang et al. (2019) ; Yoon et al. (2020) ; Zhang et al. (2024a) ; Zheng et al. (2019) ; Zhu et al. (2023), Amani et al. (2017) ; Asbaghian-Namin et al. (2021) ; Assari et al. (2020) ; Bao et al. (2021) ; Bölükbaşi et al. (2022) ; Bott-Neto et al. (2022) ; Chen et al. (2023) ; Ehzari et al. (2020) ; El Harakeh et al. (2009) ; El-Said et al. (2020) ; Gangopadhyay et al. (2024) ; Ghanei-Motlagh and Taher (2018) ; Hao et al. (2019) ; Hughes et al. (2021) ; Lin et al. (2023) ; Ma et al. (2018) ; Ma et al. (2021) ; Martins et al. (2021) ; Olorundare et al. (2023) ; Pei et al. (2024) ; Qin et al. (2023) ; Riaz et al. (2009) ; Rong et al. (2021) ; Tang et al. (2023) ; Wu et al. (2023) ; Yan et al. (2015) ; Yan et al. (2022) ; Zeng et al. (2013) ; Zhang et al. (2024b) ; Zhong et al. (2021b), (Baumgardner et al. (2010) ; Biswas et al. (2021) ; Bushira et al. (2022) ; Chen et al. (2022) ; Cotchim et al. (2024) ; Fan et al. (2013) ; Gharehaghaji et al. (2024) ; Gholamin et al. (2023) ; Gillen et al. (2010) ; Hu et al. (2023) ; Kilimci et al. (2025) ; Ma et al. (2020) ; Park et al. (2009) ; Qi et al. (2024) ; Qiu et al. (2022) ; Razmi and Mohammad-Rezaei (2013) ; Wang et al. (2013b) ; Xu et al. (2023) ; Yang et al. (2025) ; Zhou et al. (2025), Baumgardner et al. (2010) ; Biswas et al. (2021) ; Bushira et al. (2022) ; Chen et al. (2022) ; Cotchim et al. (2024) ; Fan et al. (2013) ; Gharehaghaji et al. (2024) ; Gholamin et al. (2023) ; Gillen et al. (2010) ; Hu et al. (2023) ; Kilimci et al. (2025) ; Ma et al. (2020) ; Park et al. (2009) ; Qi et al. (2024) ; Qiu et al. (2022) ; Razmi and Mohammad-Rezaei (2013) ; Wang et al. (2013b) ; Xu et al. (2023) ; Yang et al. (2025) ; Zhou et al. (2025), (Afzali and Fayazi (2016) ; Amir et al. (2024) ; Badkoobehhezaveh et al. (2018) ; Bi et al. (2023) ; Devaraji and Gopinath (2018) ; Dou et al. (2016) ; Ehzari and Safari (2022) ; Kesavan and Chen (2021) ; Ong et al. (2016) ; Roskoski (2014) ; Sakthivel et al. (2024) ; Sha et al. (2015) ; Wu et al. (2025a) ; Zhang et al. (2019), Afzali and Fayazi (2016), Amir et al. (2024), Badkoobehhezaveh et al. (2018), Bi et al. (2023), Devaraji and Gopinath (2018), Dou et al. (2016), Ehzari and Safari (2022), Kesavan and Chen (2021), Ong et al. (2016), Roskoski (2014), Sakthivel et al. (2024), Sha et al. (2015), Wu et al. (2025a), Zhang et al. (2019)
Sensitivity	Moderate	High to ultra-high owing to signal amplification	Augustine et al. (2019), Chen et al. (2020b), Cui et al. (2007), Deng et al. (2020), Lilja et al. (2008), Lin et al. (2021), Liu et al. (2022), Ma et al. (2016), Nasrollahpour et al. (2023), Pathak et al. (2017), Qin et al. (2019), Ranjan et al. (2024), Ren et al. (2025), Shang et al. (2021), Shanmugam et al. (2015), Srivastava et al. (2018), Tao and Rouhi (2023), Wang et al. (2011), Wang et al. (2013a), Wang et al. (2022), Yang et al. (2019), Yoon et al. (2020), Zhang et al. (2024a), Zheng et al. (2019), Zhu et al. (2023), Amani et al. (2017) ; Asbaghian-Namin et al. (2021) ; Assari et al. (2020) ; Bao et al. (2021) ; Bölükbaşi et al. (2022) ; Bott-Neto et al. (2022) ; Chen et al. (2023) ; Ehzari et al. (2020) ; El Harakeh et al. (2009) ; El-Said et al. (2020) ; Gangopadhyay et al. (2024) ; Ghanei-Motlagh and Taher (2018) ; Hao et al. (2019) ; Hughes et al. (2021) ; Lin et al. (2023) ; Ma et al. (2018) ; Ma et al. (2021) ; Martins et al. (2021) ; Olorundare et al. (2023) ; Pei et al. (2024) ; Qin et al. (2023) ; Riaz et al. (2009) ; Rong et al. (2021) ; Tang et al. (2023) ; Wu et al. (2023) ; Yan et al. (2015) ; Yan et al. (2022) ; Zeng et al. (2013) ; Zhang et al. (2024b) ; Zhong et al. (2021b), (Baumgardner et al. (2010) ; Biswas et al. (2021) ; Bushira et al. (2022) ; Chen et al. (2022) ; Cotchim et al. (2024) ; Fan et al. (2013) ; Gharehaghaji et al. (2024) ; Gholamin et al. (2023) ; Gillen et al. (2010) ; Hu et al. (2023) ; Kilimci et al. (2025) ; Ma et al. (2020) ; Park et al. (2009) ; Qi et al. (2024) ; Qiu et al. (2022) ; Razmi and Mohammad-Rezaei (2013) ; Wang et al. (2013b) ; Xu et al. (2023) ; Yang et al. (2025) ; Zhou et al. (2025), Baumgardner et al. (2010) ; Biswas et al. (2021) ; Bushira et al. (2022) ; Chen et al. (2022) ; Cotchim et al. (2024) ; Fan et al. (2013) ; Gharehaghaji et al. (2024) ; Gholamin et al. (2023) ; Gillen et al. (2010) ; Hu et al. (2023) ; Kilimci et al. (2025) ; Ma et al. (2020) ; Park et al. (2009) ; Qi et al. (2024) ; Qiu et al. (2022) ; Razmi and Mohammad-Rezaei (2013) ; Wang et al. (2013b) ; Xu et al. (2023) ; Yang et al. (2025) ; Zhou et al. (2025), (Afzali and Fayazi (2016) ; Amir et al. (2024) ; Badkoobehhezaveh et al. (2018) ; Bi et al. (2023) ; Devaraji and Gopinath (2018) ; Dou et al. (2016) ; Ehzari and Safari (2022) ; Kesavan and Chen (2021) ; Ong et al. (2016) ; Roskoski (2014) ; Sakthivel et al. (2024) ; Sha et al. (2015) ; Wu et al. (2025a) ; Zhang et al. (2019) (Afzali and Fayazi (2016), Amir et al. (2024), Badkoobehhezaveh et al. (2018), Bi et al. (2023), Devaraji and Gopinath (2018), Dou et al. (2016), Ehzari and Safari (2022), Kesavan and Chen (2021), Ong et al. (2016), Roskoski (2014), Sakthivel et al. (2024), Sha et al. (2015), Wu et al. (2025a), Zhang et al. (2019)
Detection limit	ng mL-1 level	pg–fg mL-1 level	Augustine et al. (2019), Chen et al. (2020b), Cui et al. (2007), Deng et al. (2020), Lilja et al. (2008), Lin et al. (2021), Liu et al. (2022), Ma et al. (2016), Nasrollahpour et al. (2023), Pathak et al. (2017), Qin et al. (2019), Ranjan et al. (2024), Ren et al. (2025), Shang et al. (2021), Shanmugam et al. (2015), Srivastava et al. (2018), Tao and Rouhi (2023), Wang et al. (2011), Wang et al. (2013a), Wang et al. (2022), Yang et al. (2019), Yoon et al. (2020), Zhang et al. (2024a), Zheng et al. (2019), Zhu et al. (2023), Amani et al. (2017) ; Asbaghian-Namin et al. (2021) ; Assari et al. (2020) ; Bao et al. (2021) ; Bölükbaşi et al. (2022) ; Bott-Neto et al. (2022) ; Chen et al. (2023) ; Ehzari et al. (2020) ; El Harakeh et al. (2009) ; El-Said et al. (2020) ; Gangopadhyay et al. (2024) ; Ghanei-Motlagh and Taher (2018) ; Hao et al. (2019) ; Hughes et al. (2021) ; Lin et al. (2023) ; Ma et al. (2018) ; Ma et al. (2021) ; Martins et al. (2021) ; Olorundare et al. (2023) ; Pei et al. (2024) ; Qin et al. (2023) ; Riaz et al. (2009) ; Rong et al. (2021) ; Tang et al. (2023) ; Wu et al. (2023) ; Yan et al. (2015) ; Yan et al. (2022) ; Zeng et al. (2013) ; Zhang et al. (2024b) ; Zhong et al. (2021b)), (Baumgardner et al. (2010) ; Biswas et al. (2021) ; Bushira et al. (2022) ; Chen et al. (2022) ; Cotchim et al. (2024) ; Fan et al. (2013) ; Gharehaghaji et al. (2024) ; Gholamin et al. (2023) ; Gillen et al. (2010) ; Hu et al. (2023) ; Kilimci et al. (2025) ; Ma et al. (2020) ; Park et al. (2009) ; Qi et al. (2024) ; Qiu et al. (2022) ; Razmi and Mohammad-Rezaei (2013) ; Wang et al. (2013b) ; Xu et al. (2023) ; Yang et al. (2025) ; Zhou et al. (2025), Baumgardner et al. (2010) ; Biswas et al. (2021) ; Bushira et al. (2022) ; Chen et al. (2022) ; Cotchim et al. (2024) ; Fan et al. (2013) ; Gharehaghaji et al. (2024) ; Gholamin et al. (2023) ; Gillen et al. (2010) ; Hu et al. (2023) ; Kilimci et al. (2025) ; Ma et al. (2020) ; Park et al. (2009) ; Qi et al. (2024) ; Qiu et al. (2022) ; Razmi and Mohammad-Rezaei (2013) ; Wang et al. (2013b) ; Xu et al. (2023) ; Yang et al. (2025) ; Zhou et al. (2025), (Afzali and Fayazi (2016) ; Amir et al. (2024) ; Badkoobehhezaveh et al. (2018) ; Bi et al. (2023) ; Devaraji and Gopinath (2018) ; Dou et al. (2016) ; Ehzari and Safari (2022) ; Kesavan and Chen (2021) ; Ong et al. (2016) ; Roskoski (2014) ; Sakthivel et al. (2024) ; Sha et al. (2015) ; Wu et al. (2025a) ; Zhang et al. (2019) (Afzali and Fayazi (2016), Amir et al. (2024), Badkoobehhezaveh et al. (2018), Bi et al. (2023), Devaraji and Gopinath (2018), Dou et al. (2016), Ehzari and Safari (2022), Kesavan and Chen (2021), Ong et al. (2016), Roskoski (2014), Sakthivel et al. (2024), Sha et al. (2015), Wu et al. (2025a), Zhang et al. (2019)
Dynamic range	Relatively narrow	Broad	Augustine et al. (2019), Chen et al. (2020b), Cui et al. (2007), Deng et al. (2020), Lilja et al. (2008), Lin et al. (2021), Liu et al. (2022), Ma et al. (2016), Nasrollahpour et al. (2023), Pathak et al. (2017), Qin et al. (2019), Ranjan et al. (2024), Ren et al. (2025), Shang et al. (2021), Shanmugam et al. (2015), Srivastava et al. (2018), Tao and Rouhi (2023), Wang et al. (2011), Wang et al. (2013a), Wang et al. (2022), Yang et al. (2019), Yoon et al. (2020), Zhang et al. (2024a), Zheng et al. (2019), Zhu et al. (2023), Amani et al. (2017) ; Asbaghian-Namin et al. (2021) ; Assari et al. (2020) ; Bao et al. (2021) ; Bölükbaşi et al. (2022) ; Bott-Neto et al. (2022) ; Chen et al. (2023) ; Ehzari et al. (2020) ; El Harakeh et al. (2009) ; El-Said et al. (2020) ; Gangopadhyay et al. (2024) ; Ghanei-Motlagh and Taher (2018) ; Hao et al. (2019) ; Hughes et al. (2021) ; Lin et al. (2023) ; Ma et al. (2018) ; Ma et al. (2021) ; Martins et al. (2021) ; Olorundare et al. (2023) ; Pei et al. (2024) ; Qin et al. (2023) ; Riaz et al. (2009) ; Rong et al. (2021) ; Tang et al. (2023) ; Wu et al. (2023) ; Yan et al. (2015) ; Yan et al. (2022) ; Zeng et al. (2013) ; Zhang et al. (2024b) ; Zhong et al. (2021b), (Baumgardner et al. (2010) ; Biswas et al. (2021) ; Bushira et al. (2022) ; Chen et al. (2022) ; Cotchim et al. (2024) ; Fan et al. (2013) ; Gharehaghaji et al. (2024) ; Gholamin et al. (2023) ; Gillen et al. (2010) ; Hu et al. (2023) ; Kilimci et al. (2025) ; Ma et al. (2020) ; Park et al. (2009) ; Qi et al. (2024) ; Qiu et al. (2022) ; Razmi and Mohammad-Rezaei (2013) ; Wang et al. (2013b) ; Xu et al. (2023) ; Yang et al. (2025) ; Zhou et al. (2025), Baumgardner et al. (2010) ; Biswas et al. (2021) ; Bushira et al. (2022) ; Chen et al. (2022) ; Cotchim et al. (2024) ; Fan et al. (2013) ; Gharehaghaji et al. (2024) ; Gholamin et al. (2023) ; Gillen et al. (2010) ; Hu et al. (2023) ; Kilimci et al. (2025) ; Ma et al. (2020) ; Park et al. (2009) ; Qi et al. (2024) ; Qiu et al. (2022) ; Razmi and Mohammad-Rezaei (2013) ; Wang et al. (2013b) ; Xu et al. (2023) ; Yang et al. (2025) ; Zhou et al. (2025), (Afzali and Fayazi (2016) ; Amir et al. (2024) ; Badkoobehhezaveh et al. (2018) ; Bi et al. (2023) ; Devaraji and Gopinath (2018) ; Dou et al. (2016) ; Ehzari and Safari (2022) ; Kesavan and Chen (2021) ; Ong et al. (2016) ; Roskoski (2014) ; Sakthivel et al. (2024) ; Sha et al. (2015) ; Wu et al. (2025a) ; Zhang et al. (2019) Afzali and Fayazi (2016), Amir et al. (2024), Badkoobehhezaveh et al. (2018), Bi et al. (2023), Devaraji and Gopinath (2018), Dou et al. (2016), Ehzari and Safari (2022), Kesavan and Chen (2021), Ong et al. (2016), Roskoski (2014), Sakthivel et al. (2024), Sha et al. (2015), Wu et al. (2025a), Zhang et al. (2019)
Response time	Usually longer	Generally shorter	Augustine et al. (2019), Chen et al. (2020b), Cui et al. (2007), Deng et al. (2020), Lilja et al. (2008), Lin et al. (2021), Liu et al. (2022), Ma et al. (2016), Nasrollahpour et al. (2023), Pathak et al. (2017), Qin et al. (2019), Ranjan et al. (2024), Ren et al. (2025), Shang et al. (2021), Shanmugam et al. (2015), Srivastava et al. (2018), Tao and Rouhi (2023), Wang et al. (2011), Wang et al. (2013a), Wang et al. (2022), Yang et al. (2019), Yoon et al. (2020), Zhang et al. (2024a), Zheng et al. (2019), Zhu et al. (2023), Amani et al. (2017) ; Asbaghian-Namin et al. (2021) ; Assari et al. (2020) ; Bao et al. (2021) ; Bölükbaşi et al. (2022) ; Bott-Neto et al. (2022) ; Chen et al. (2023) ; Ehzari et al. (2020) ; El Harakeh et al. (2009) ; El-Said et al. (2020) ; Gangopadhyay et al. (2024) ; Ghanei-Motlagh and Taher (2018) ; Hao et al. (2019) ; Hughes et al. (2021) ; Lin et al. (2023) ; Ma et al. (2018) ; Ma et al. (2021) ; Martins et al. (2021) ; Olorundare et al. (2023) ; Pei et al. (2024) ; Qin et al. (2023) ; Riaz et al. (2009) ; Rong et al. (2021) ; Tang et al. (2023) ; Wu et al. (2023) ; Yan et al. (2015) ; Yan et al. (2022) ; Zeng et al. (2013) ; Zhang et al. (2024b) ; Zhong et al. (2021b)), (Baumgardner et al. (2010) ; Biswas et al. (2021) ; Bushira et al. (2022) ; Chen et al. (2022) ; Cotchim et al. (2024) ; Fan et al. (2013) ; Gharehaghaji et al. (2024) ; Gholamin et al. (2023) ; Gillen et al. (2010) ; Hu et al. (2023) ; Kilimci et al. (2025) ; Ma et al. (2020) ; Park et al. (2009) ; Qi et al. (2024) ; Qiu et al. (2022) ; Razmi and Mohammad-Rezaei (2013) ; Wang et al. (2013b) ; Xu et al. (2023) ; Yang et al. (2025) ; Zhou et al. (2025), Baumgardner et al. (2010) ; Biswas et al. (2021) ; Bushira et al. (2022) ; Chen et al. (2022) ; Cotchim et al. (2024) ; Fan et al. (2013) ; Gharehaghaji et al. (2024) ; Gholamin et al. (2023) ; Gillen et al. (2010) ; Hu et al. (2023) ; Kilimci et al. (2025) ; Ma et al. (2020) ; Park et al. (2009) ; Qi et al. (2024) ; Qiu et al. (2022) ; Razmi and Mohammad-Rezaei (2013) ; Wang et al. (2013b) ; Xu et al. (2023) ; Yang et al. (2025) ; Zhou et al. (2025), (Afzali and Fayazi (2016) ; Amir et al. (2024) ; Badkoobehhezaveh et al. (2018) ; Bi et al. (2023) ; Devaraji and Gopinath (2018) ; Dou et al. (2016) ; Ehzari and Safari (2022) ; Kesavan and Chen (2021) ; Ong et al. (2016) ; Roskoski (2014) ; Sakthivel et al. (2024) ; Sha et al. (2015) ; Wu et al. (2025a) ; Zhang et al. (2019) Afzali and Fayazi (2016), Amir et al. (2024), Badkoobehhezaveh et al. (2018), Bi et al. (2023), Devaraji and Gopinath (2018), Dou et al. (2016), Ehzari and Safari (2022), Kesavan and Chen (2021), Ong et al. (2016), Roskoski (2014), Sakthivel et al. (2024), Sha et al. (2015), Wu et al. (2025a), Zhang et al. (2019)
Multiplex detection	Limited	Readily achievable	Amir et al. (2024), Cotchim et al. (2024), Ehzari and Safari (2022), Sakthivel et al. (2024), Shang et al. (2021), Tang et al. (2023), Zhou et al. (2025)
Instrumentation	Mature clinical instruments	Electrochemical/photoelectrochemical/electrochemiluminescence platforms	Yola (2021), Chen et al. (2020b) ; Wang et al. (2011) ; Wang et al. (2022) ; Zheng et al. (2019) ; Zhu et al. (2023), Amani et al. (2017) ; Asbaghian-Namin et al. (2021) ; Assari et al. (2020) ; Augustine et al. (2019) ; Bao et al. (2021) ; Bott-Neto et al. (2022) ; Deng et al. (2020) ; Ehzari et al. (2020) ; El-Said et al. (2020) ; Lilja et al. (2008) ; Lin et al. (2021) ; Lin et al. (2023) ; Liu et al. (2022) ; Ma et al. (2018) ; Ma et al. (2021) ; Nasrollahpour et al. (2023) ; Pathak et al. (2017) ; Ranjan et al. (2024) ; Ren et al. (2025) ; Shang et al. (2021) ; Srivastava et al. (2018) ; Tao and Rouhi (2023) ; Yan et al. (2022) ; Yoon et al. (2020) ; Zhang et al. (2024a), (Baumgardner et al. (2010) ; Biswas et al. (2021) ; Bushira et al. (2022) ; Chen et al. (2022) ; Cotchim et al. (2024) ; Fan et al. (2013) ; Gharehaghaji et al. (2024) ; Gholamin et al. (2023) ; Gillen et al. (2010) ; Hu et al. (2023) ; Kilimci et al. (2025) ; Ma et al. (2020) ; Park et al. (2009) ; Qi et al. (2024) ; Qin et al. (2023) ; Qiu et al. (2022) ; Razmi and Mohammad-Rezaei (2013) ; Tang et al. (2023) ; Wang et al. (2013b) ; Wu et al. (2025a) ; Xu et al. (2023) ; Yang et al. (2025) ; Zhong et al. (2021b) ; Zhou et al. (2025), (Amir et al. (2024), Badkoobehhezaveh et al. (2018), Bi et al. (2023), Devaraji and Gopinath (2018), Ehzari and Safari (2022), Ong et al. (2016), Sakthivel et al. (2024), Zhang et al. (2019)
Cost	Moderate to high	Potentially lower reagent consumption but higher material preparation cost	Ahn et al. (2021), Galle et al. (2019), Stenman et al. (1999), Yola (2021), Augustine et al. (2019) ; Chen et al. (2020b) ; Cui et al. (2007) ; Deng et al. (2020) ; Lilja et al. (2008) ; Lin et al. (2021) ; Liu et al. (2022) ; Ma et al. (2016) ; Nasrollahpour et al. (2023) ; Pathak et al. (2017) ; Qin et al. (2019) ; Ranjan et al. (2024) ; Ren et al. (2025) ; Shang et al. (2021) ; Shanmugam et al. (2015) ; Srivastava et al. (2018) ; Tao and Rouhi (2023) ; Wang et al. (2011) ; Wang et al. (2013a) ; Wang et al. (2022) ; Yang et al. (2019) ; Yoon et al. (2020) ; Zhang et al. (2024a) ; Zheng et al. (2019) ; Zhu et al. (2023), Amani et al. (2017) ; Asbaghian-Namin et al. (2021) ; Assari et al. (2020) ; Bao et al. (2021) ; Bölükbaşi et al. (2022) ; Bott-Neto et al. (2022) ; Chen et al. (2023) ; Ehzari et al. (2020) ; El Harakeh et al. (2009) ; El-Said et al. (2020) ; Gangopadhyay et al. (2024) ; Ghanei-Motlagh and Taher (2018) ; Hao et al. (2019) ; Hughes et al. (2021) ; Lin et al. (2023) ; Ma et al. (2018) ; Ma et al. (2021) ; Martins et al. (2021) ; Olorundare et al. (2023) ; Pei et al. (2024) ; Qin et al. (2023) ; Riaz et al. (2009) ; Rong et al. (2021) ; Tang et al. (2023) ; Wu et al. (2023) ; Yan et al. (2015) ; Yan et al. (2022) ; Zeng et al. (2013) ; Zhang et al. (2024b) ; Zhong et al. (2021b), Baumgardner et al. (2010) ; Biswas et al. (2021) ; Bushira et al. (2022) ; Chen et al. (2022) ; Cotchim et al. (2024) ; Fan et al. (2013) ; Gharehaghaji et al. (2024) ; Gholamin et al. (2023) ; Gillen et al. (2010) ; Hu et al. (2023) ; Kilimci et al. (2025) ; Ma et al. (2020) ; Park et al. (2009) ; Qi et al. (2024) ; Qiu et al. (2022) ; Razmi and Mohammad-Rezaei (2013) ; Wang et al. (2013b) ; Xu et al. (2023) ; Yang et al. (2025) ; Zhou et al. (2025)–Baumgardner et al. (2010) ; Biswas et al. (2021) ; Bushira et al. (2022) ; Chen et al. (2022) ; Cotchim et al. (2024) ; Fan et al. (2013) ; Gharehaghaji et al. (2024) ; Gholamin et al. (2023) ; Gillen et al. (2010) ; Hu et al. (2023) ; Kilimci et al. (2025) ; Ma et al. (2020) ; Park et al. (2009) ; Qi et al. (2024) ; Qiu et al. (2022) ; Razmi and Mohammad-Rezaei (2013) ; Wang et al. (2013b) ; Xu et al. (2023) ; Yang et al. (2025) ; Zhou et al. (2025), (Afzali and Fayazi (2016) ; Amir et al. (2024) ; Badkoobehhezaveh et al. (2018) ; Bi et al. (2023) ; Devaraji and Gopinath (2018) ; Dou et al. (2016) ; Ehzari and Safari (2022) ; Kesavan and Chen (2021) ; Ong et al. (2016) ; Roskoski (2014) ; Sakthivel et al. (2024) ; Sha et al. (2015) ; Wu et al. (2025a) ; Zhang et al. (2019) (Afzali and Fayazi (2016), Amir et al. (2024), Badkoobehhezaveh et al. (2018), Bi et al. (2023), Devaraji and Gopinath (2018), Dou et al. (2016), Ehzari and Safari (2022), Kesavan and Chen (2021), Ong et al. (2016), Roskoski (2014), Sakthivel et al. (2024), Sha et al. (2015), Wu et al. (2025a), Zhang et al. (2019)
Clinical application	Widely established	Mostly laboratory research with promising clinical translation	Brunstein (2016), Cao et al. (2023), Ho et al. (2024), Li et al. (2017), Zaak et al. (2001), (Ahn et al. (2021) ; Galle et al. (2019) ; Stenman et al. (1999), (Yola (2021), Augustine et al. (2019) ; Chen et al. (2020b) ; Cui et al. (2007) ; Deng et al. (2020) ; Lilja et al. (2008) ; Lin et al. (2021) ; Liu et al. (2022) ; Ma et al. (2016) ; Nasrollahpour et al. (2023) ; Pathak et al. (2017) ; Qin et al. (2019) ; Ranjan et al. (2024) ; Ren et al. (2025) ; Shang et al. (2021) ; Shanmugam et al. (2015) ; Srivastava et al. (2018) ; Tao and Rouhi (2023) ; Wang et al. (2011) ; Wang et al. (2013a) ; Wang et al. (2022) ; Yang et al. (2019) ; Yoon et al. (2020) ; Zhang et al. (2024a) ; Zheng et al. (2019) ; Zhu et al. (2023), Amani et al. (2017) ; Asbaghian-Namin et al. (2021) ; Assari et al. (2020) ; Bao et al. (2021) ; Bölükbaşi et al. (2022) ; Bott-Neto et al. (2022) ; Chen et al. (2023) ; Ehzari et al. (2020) ; El Harakeh et al. (2009) ; El-Said et al. (2020) ; Gangopadhyay et al. (2024) ; Ghanei-Motlagh and Taher (2018) ; Hao et al. (2019) ; Hughes et al. (2021) ; Lin et al. (2023) ; Ma et al. (2018) ; Ma et al. (2021) ; Martins et al. (2021) ; Olorundare et al. (2023) ; Pei et al. (2024) ; Qin et al. (2023) ; Riaz et al. (2009) ; Rong et al. (2021) ; Tang et al. (2023) ; Wu et al. (2023) ; Yan et al. (2015) ; Yan et al. (2022) ; Zeng et al. (2013) ; Zhang et al. (2024b) ; Zhong et al. (2021b)), (Baumgardner et al. (2010) ; Biswas et al. (2021) ; Bushira et al. (2022) ; Chen et al. (2022) ; Cotchim et al. (2024) ; Fan et al. (2013) ; Gharehaghaji et al. (2024) ; Gholamin et al. (2023) ; Gillen et al. (2010) ; Hu et al. (2023) ; Kilimci et al. (2025) ; Ma et al. (2020) ; Park et al. (2009) ; Qi et al. (2024) ; Qiu et al. (2022) ; Razmi and Mohammad-Rezaei (2013) ; Wang et al. (2013b) ; Xu et al. (2023) ; Yang et al. (2025) ; Zhou et al. (2025), Baumgardner et al. (2010) ; Biswas et al. (2021) ; Bushira et al. (2022) ; Chen et al. (2022) ; Cotchim et al. (2024) ; Fan et al. (2013) ; Gharehaghaji et al. (2024) ; Gholamin et al. (2023) ; Gillen et al. (2010) ; Hu et al. (2023) ; Kilimci et al. (2025) ; Ma et al. (2020) ; Park et al. (2009) ; Qi et al. (2024) ; Qiu et al. (2022) ; Razmi and Mohammad-Rezaei (2013) ; Wang et al. (2013b) ; Xu et al. (2023) ; Yang et al. (2025) ; Zhou et al. (2025), (Afzali and Fayazi (2016) ; Amir et al. (2024) ; Badkoobehhezaveh et al. (2018) ; Bi et al. (2023) ; Devaraji and Gopinath (2018) ; Dou et al. (2016) ; Ehzari and Safari (2022) ; Kesavan and Chen (2021) ; Ong et al. (2016) ; Roskoski (2014) ; Sakthivel et al. (2024) ; Sha et al. (2015) ; Wu et al. (2025a) ; Zhang et al. (2019) (Afzali and Fayazi (2016), Amir et al. (2024), Badkoobehhezaveh et al. (2018), Bi et al. (2023), Devaraji and Gopinath (2018), Dou et al. (2016), Ehzari and Safari (2022), Kesavan and Chen (2021), Ong et al. (2016), Roskoski (2014), Sakthivel et al. (2024), Sha et al. (2015), Wu et al. (2025a), Zhang et al. (2019)

Data compiled by the authors based on References.

To address these technical challenges, researchers have adopted an interdisciplinary approach integrating nanotechnology with immunology over the past 5 years, incorporating advanced nanomaterials into immunosensor design to develop next-generation diagnostic platforms. Nanomaterials (1–100 nm) demonstrate exceptional optoelectronic properties and bio-interactive capabilities stemming from surface effects, quantum confinement, and macroscopic quantum tunneling phenomena (Li et al., 2023; Padmanabhan and Kyriakides, 2015; Rao and Shi, 2022). Representative materials such as NPs, QDs, CNTs, and graphene are frequently employed as nanomaterials in immunosensor applications, enhancing device performance through three primary mechanisms: First, surface plasmon resonance effects enable the conversion of antigen-antibody binding events into quantifiable optical signals; second, their high specific surface areas improve antibody immobilization efficiency; and third, quantum confinement effects facilitate single-molecule level signal amplification (Lai et al., 2018; Viswambari Devi et al., 2015). These attributes enable nanomaterial-based immunosensors to achieve ultra-sensitive detection of trace tumor markers in diverse biological matrices (e.g., blood, saliva). For instance, the immunosensor utilizing gold nanoparticles (AuNPs) achieves optical signal transduction through surface plasmon resonance effects (mechanism 1). Its high specific surface area enables efficient antibody immobilization within 15 min (mechanism 2), while quantum confinement effects amplify electrochemical signals by accelerating electron transfer kinetics (mechanism 3). The synergistic combination of these mechanisms results in a HER2 detection limit of 3 fg mL^-1^ (100-fold improvement over conventional ELISA) with a total assay time compressed to under 30 minutes—representing over 90% time reduction compared to the 4–6 h required for traditional ELISA, achieving a dual breakthrough in sensitivity and detection efficiency (Yola, 2021).

Based on these considerations, this review discusses recent advances in nanomaterial-enhanced immunosensors for clinically relevant tumor biomarkers, including carcinoembryonic antigen (CEA), prostate-specific antigen (PSA), cancer antigen 153 (CA153), alpha-fetoprotein (AFP), cancer antigen 125 (CA125), cancer antigen 199 (CA199), and human epidermal growth factor receptor 2 (HER2). To avoid repetitive marker-by-marker descriptions and hierarchical overlap among nanomaterial categories, the manuscript adopts a mechanism-oriented and platform-centered organization. Specifically, electrochemical, photoelectrochemical, electrochemiluminescent, and chemiluminescent immunosensors are discussed as the major signal transduction platforms, while individual tumor biomarkers are presented as representative applications within these platforms. Within each sensing strategy, representative nanomaterials, including noble metal nanoparticles, quantum dots (QDs), carbon nanotubes (CNTs), graphene derivatives, MXenes, metal-organic frameworks (MOFs), covalent organic frameworks (COFs), and related nanocomposites, are analyzed according to their functional contributions to antibody immobilization, electron-transfer acceleration, catalytic amplification, photoinduced charge separation, luminescence enhancement, magnetic enrichment, anti-interference capability, and interface stabilization. This organization emphasizes that nanomaterials act as functional modules within specific sensing mechanisms rather than as independent classification endpoints. To further strengthen comparative analysis, Tables 3, 4 summarize representative platforms and compare their sensitivity, detection limits, specificity, stability, cost, scalability, and clinical applicability. This framework provides a clearer basis for evaluating which sensing platforms are most suitable for future clinical translation and point-of-care tumor marker detection.

**TABLE 3:** Platform-centered summary of representative nanomaterial-enhanced immunosensors for tumor marker detection.

Platform	Representative biomarkers	Representative systems	Main nanomaterial functions	Analytical strengths	Major limitations
Electrochemistry	CEA, PSA, CA153, AFP, CA125, CA199, HER2	SWCNTs/AuNPs; MXene-MWCNTs/AuNPs; MoO_3_-RGO-IL; PQQ-CNT; MWCNTs/PANI/AuNPs; AuPt-BG; NiCo@Fc-MWCNTs-LDH/3D-RGO@Ag/Au; Au/MXene/NG-CuMnCoO_x_	Electron-transfer acceleration, antibody immobilization, catalytic current amplification, magnetic enrichment, ratiometric signal correction	Low cost, simple readout, miniaturization potential, broad biomarker coverage	Matrix interference, electrode variability, fouling, need for standard calibration
Photoelectrochemistry	CEA, PSA, CA199	Au@WP5-PANI-BiOBr; PB-PDA/BiOI; CdS/V_2_O_5_; TiO_2_/Ni-g-C_3_N_4_; SnSe QDs	Light absorption, photoinduced charge separation, heterojunction-mediated recombination suppression, photocurrent modulation	Low background, high sensitivity, strong signal-to-noise ratio	Requires light source, photoelectrode stability, and reproducible semiconductor interfaces
Electrochemiluminescence	CEA, PSA, CA153, CA125, CA199, HER2	CS-AgNPs-luminol; Fe_3_O_4_@TMU-10/Ni-CdTe QDs; PPY-luminol-AuNPs; Pt/RGO/CeO_2_; N-GQDs/PtNPs; Pt-doped CdTe QDs	Luminescence enhancement, coreactant acceleration, quantum-dot signal amplification, magnetic enrichment, quenching/enhancement modulation	Very low detection limits, wide linear ranges, low optical background	Complex probe chemistry, stability concerns, higher fabrication complexity
Chemiluminescence	CEA and selected tumor markers	CuFe_2_O_4_@Au-ABEI; nanozyme-assisted chemiluminescent systems	Catalytic light generation, magnetic enrichment, electron-transfer-mediated chemiluminescent amplification	No external excitation source, simplified optical readout, potential for rapid assays	Quantitative stability, reagent storage, and device integration require improvement

**TABLE 4:** Cross-platform comparison of analytical performance and clinical applicability.

Platform	Sensitivity/detection limit	Specificity/selectivity	Stability	Cost	Scalability	Clinical applicability	Key translational issue	References
Electrochemistry	fg–pg·mL^-1^ to ng·mL^-1^ depending on interface and format	Good when antibody orientation and blocking are optimized; vulnerable to matrix fouling	Moderate to good; depends on electrode reproducibility and antifouling layer	Low to moderate	High; compatible with screen-printed and portable electrodes	High for point-of-care and decentralized testing	Need batch-to-batch reproducibility, antifouling validation, and clinical calibration	Assari et al. (2020), Augustine et al. (2019), Bi et al. (2023), Biswas et al. (2021), Bölükbaşi et al. (2022), Chen et al. (2022), Chen et al. (2023), Cotchim et al. (2024), Deng et al. (2020), Ehzari and Safari (2022), Gangopadhyay et al. (2024), Ghanei-Motlagh and Taher (2018), Gharehaghaji et al. (2024), Hu et al. (2023), Kilimci et al. (2025), Lin et al. (2021), Ma et al. (2020), Ma et al. (2021), Martins et al. (2021), Olorundare et al. (2023), Pathak et al. (2017), Pei et al. (2024), Qi et al. (2024), Qiu et al. (2022), Ranjan et al. (2024), Rong et al. (2021), Sakthivel et al. (2024), Tao and Rouhi (2023), Wu et al. (2023), Yan et al. (2022), Yang et al. (2025), Yola (2021), Zhang et al. (2024a), Zhang et al. (2024b)
Photoelectrochemistry	Often fg–pg·mL^-1^ due to low background photocurrent	Good; steric hindrance-based signal modulation improves selectivity	Moderate; depends on semiconductor photostability and illumination conditions	Moderate	Moderate; requires controlled light source and stable photoelectrode	Promising for high-sensitivity laboratory or portable optical-electrochemical systems	Need stable photoactive interfaces and standardized illumination/readout	Bott-Neto et al. (2022), Chen et al. (2020b), Gholamin et al. (2023), Qin et al. (2023), Wang et al. (2022), Zhong et al. (2021b), Zhu et al. (2023)
Electrochemiluminescence	Frequently fg-level with wide linear ranges	High in sandwich or ratiometric formats; signal probes improve specificity	Moderate to good, but luminophore/coreactant stability must be verified	Moderate to high	Moderate; complex labeling may affect scale-up	High for ultrasensitive detection and multiplex readout	Need simplified probe synthesis and long-term storage validation	Amir et al. (2024), Asbaghian-Namin et al. (2021), Bao et al. (2021), Ehzari et al. (2020), Lin et al. (2023), Liu et al. (2022), Nasrollahpour et al. (2023), Shang et al. (2021), Tang et al. (2023), Wu et al. (2025a), Zhou et al. (2025)
Chemiluminescence	pg-level to sub-pg depending on catalytic system	Good with antibody-based recognition and magnetic separation	Variable; reagent and catalyst stability are critical	Low to moderate	Moderate; compatible with simple optical readers	Suitable for rapid assays if quantitative control is improved	Need reagent stabilization, calibration, and integrated device design	Ren et al. (2025)

Data compiled by the authors based on References.

**FIGURE 1:**
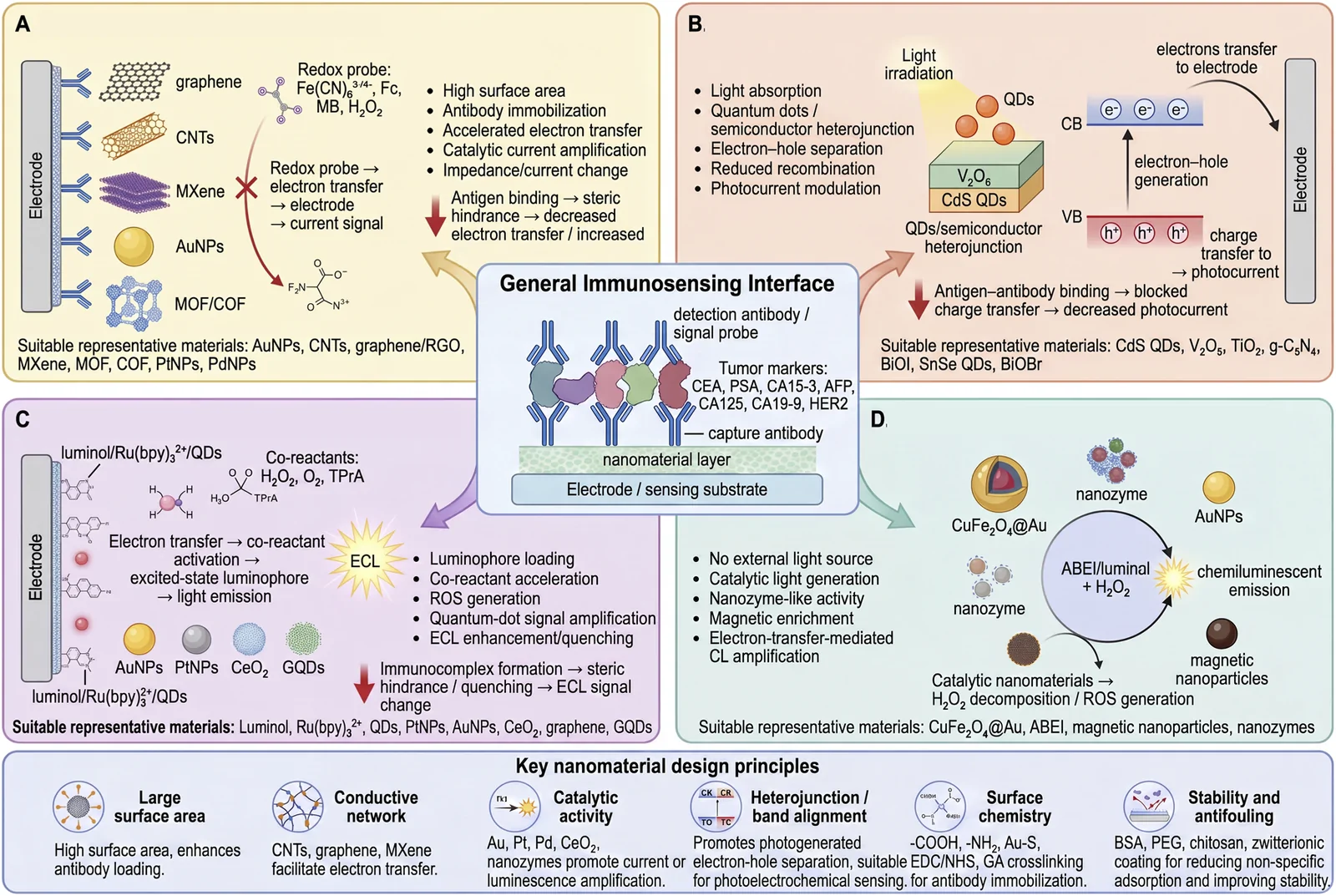
Schematic overview of signal amplification mechanisms and electron-transfer pathways in nanomaterial-enhanced immunosensors for tumor marker detection. **(A)** Electrochemical immunosensors, **(B)** Photoelectrochemical immunosensors, **(C)** Electrochemiluminescent immunosensors, **(D)** Chemiluminescent immunosensors.

To provide a clearer mechanistic overview, Figure 1 summarizes the major signal amplification mechanisms and electron-transfer pathways involved in nanomaterial-enhanced immunosensors. This schematic illustrates how different nanomaterial modules contribute to electrochemical, photoelectrochemical, electrochemiluminescent, and chemiluminescent signal transduction for tumor marker detection.

Nanomaterials enhance immunosensor performance through multiple mechanisms, including high-density antibody immobilization, accelerated electron transfer, catalytic current amplification, photoinduced charge separation, luminescence regulation, co-reactant activation, magnetic enrichment, and interface stabilization. In electrochemical immunosensors, conductive nanomaterials and catalytic nanoparticles improve interfacial electron transfer and convert antigen–antibody recognition into current or impedance changes. In photoelectrochemical immunosensors, semiconductor nanomaterials and heterojunctions promote light absorption, electron–hole separation, and photocurrent modulation. In electrochemiluminescent systems, luminophores, quantum dots, and catalytic nanocomposites regulate co-reactant reactions and light emission. In chemiluminescent platforms, nanozyme-like catalysts and magnetic nanomaterials promote reactive oxygen species generation and electron-transfer-mediated light emission. Together, these mechanisms illustrate how nanomaterials function as modular components for signal amplification and sensitive tumor marker detection. Created by the authors based on Refs. (Fawzy et al., 2016; Klein, 2021; Molina et al., 2005; Rawla et al., 2019; Wagle et al., 2025; Zhang et al., 2021).

## 2 Nanomaterial-enhanced immunosensing strategies for CEA detection

CEA is an acidic glycoprotein possessing properties characteristic of human embryonic antigens (Kailemia et al., 2017). Clinical trials have shown that elevated CEA levels are found not only in gastrointestinal malignancies but also in breast, lung, and other malignancies, with CEA recognized as a broad-spectrum tumor marker (Beauchemin et al., 2013; Seale and Tkaczuk, 2022; Shimada et al., 2014). CEA detection holds critical significance for early cancer diagnosis and therapeutic response monitoring (Qiu et al., 2017). For CEA detection, recent nanomaterial-enhanced immunosensors can be more appropriately categorized according to their signal transduction mechanisms, including photoelectrochemical, electrochemical, electrochemiluminescent, and chemiluminescent platforms. In photoelectrochemical systems, materials such as Au@WP5-PANI-BiOBr and V_2_O_5_/CdS quantum dots mainly improve light absorption, charge separation, and photocurrent conversion. In electrochemical systems, CNTs, graphene derivatives, MXene, AuNPs, and MoO_3_-based composites primarily enhance electron transfer, antibody immobilization, and catalytic current amplification. In electrochemiluminescent systems, graphene-ionic liquid matrices, Pt/Pd nanoparticles, and MoS_2_ nanoprobes contribute to dual-signal modulation and luminescence quenching/amplification. In chemiluminescent platforms, CuFe_2_O_4_@Au nanocomposites enhance catalytic activity and electron-transfer-mediated light emission. Therefore, the sensing mechanism provides the primary classification framework, whereas the nanomaterials are discussed according to their functional roles in signal amplification and analytical performance.

### 2.1 Photoelectrochemical immunosensors for CEA detection

**FIGURE 2:**
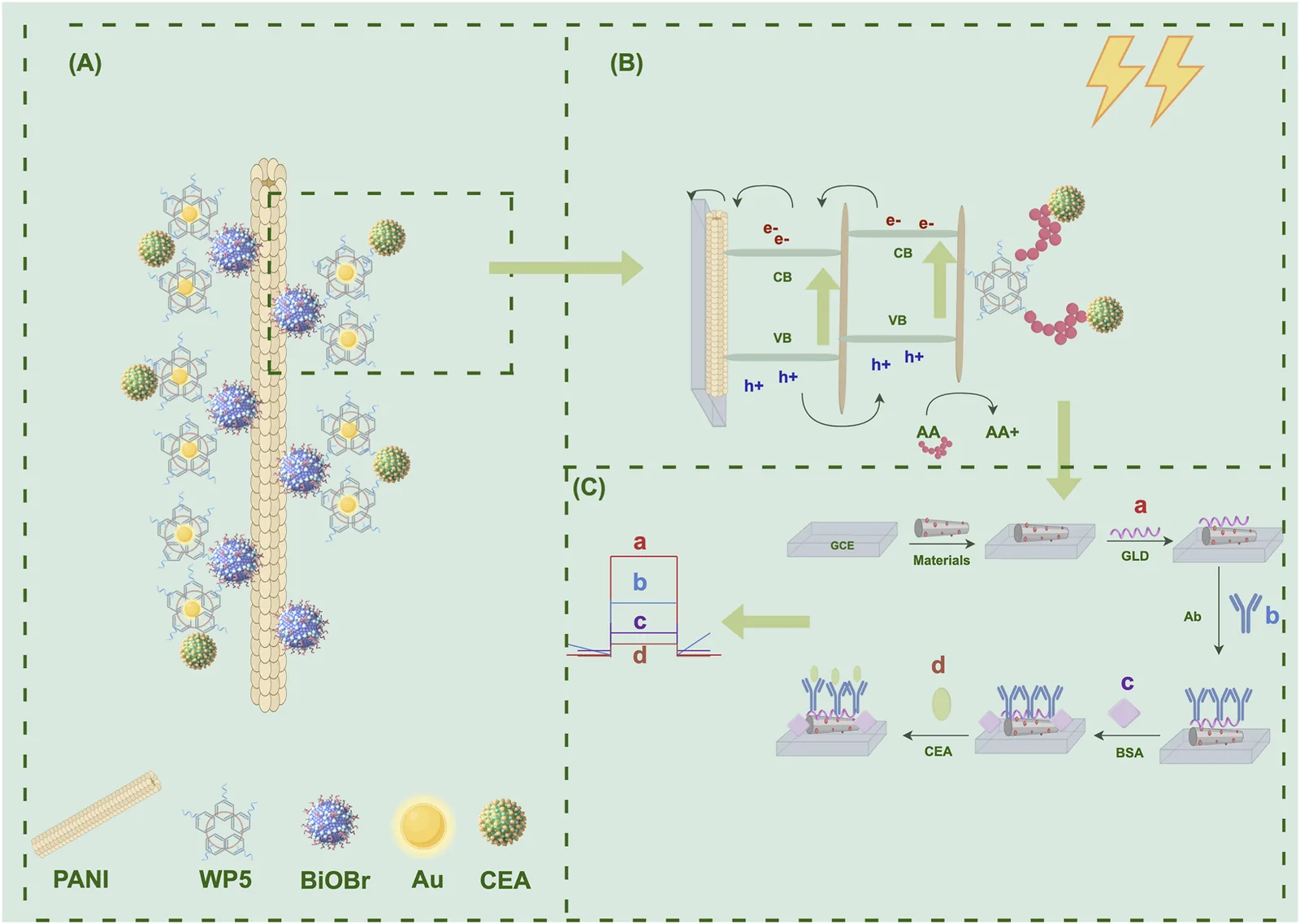
Schematic diagram of the CEA photoelectrochemical immunosensor based on Au@WP5-PANI-BiOBr nanocomposite. **(A)** Immunosensor preparation via PANI hollow tube synthesis, WP5 covalent grafting, BiOBr nanosheet hybridization, and AuNP deposition. **(B)** Signal amplification through BiOBr electron-hole separation, PANI electron transfer, and WP5-AA interaction. **(C)** Assembly including GCE surface activation, antibody immobilization, BSA blocking, and CEA detection with amperometric readout. Adapted from Ref (Wang et al., 2022). with modifications.

Wang et al. (2022) first showed ascorbic acid (AA) acts as a key electron donor modulating photocurrent intensity. They developed a photoelectrochemical immunosensor using pillar [5]arene-modified AuNP/polyaniline-BiOBr composite (Au@WP5-PANI-BiOBr). This composite leverages synergistic mechanisms—AuNPs’ plasmonic effects, pillar [5]arene-AA host-guest recognition, and PANI’s three-dimensional (3D) conductive network—to boost carrier separation and electron transfer, forming the technical basis for CEA detection. Enhancement mechanisms include: plasmon-enhanced carrier separation, accelerated AA oxidation via BiOBr holes, and efficient charge transport. These material properties directly enable a “photocurrent decay” strategy, where antibody-antigen binding blocks electron pathways to quantify targets. After bovine serum albumin (BSA) blocking, anti-CEA antibodies on the electrode immunoreacted with CEA, reducing photocurrent proportionally, as depicted in Figure 2. The sensor achieved a linear range (0.01–50.0 ng/mL) with 3.0 pg/mL sensitivity.

Zhu et al. (2023) synthesized Prussian Blue nanoparticles (PBNPs) by ferric chloride and potassium ferrocyanide precipitation, followed by polydopamine (PDA) coating via *in situ* self-polymerization at pH 8.5 to form PB-PDA nanocomposites. The PDA layer enabled antibody conjugation through Ethyldimethylaminopropyl Carbodiimide/N-Hydroxysuccinimide (EDC/NHS) coupling while preserving PBNPs’ redox activity. After anti-CEA functionalization, PB-PDA NPs were immobilized on carboxylated graphene-modified screen-printed carbon electrodes. Under alkaline hydrolysis, PB-PDA generated redox-active electron acceptors that amplified the photocurrent of microstructured BiOI photocathodes, enabling CEA detection from 1.0 × 10^−3^–100.0 ng mL^-1^ with a detection limit of 54.9 fg mL^-1^.

Band alignment between semiconductors can promote electron–hole separation (Wang et al., 2011; Zheng et al., 2019). Chen et al. (Chen Y. et al., 2020) constructed a type-II CdS/V_2_O_5_ heterojunction by etching V_2_O_5_ nanosheets to expose active sites and anchoring CdS QDs through a solvothermal process. In this photoelectrochemical immunosensor, CdS QDs enhanced light absorption and electron–hole generation, while V_2_O_5_ served as an electron acceptor to suppress recombination and improve charge separation. This synergistic structure amplified the photocurrent response, enabling CEA detection from 0.5 pg mL^-1^ to 1.0 ng mL^-1^ with a detection limit of 0.1 pg mL^-1^. Overall, the CdS/V_2_O_5_ heterojunction provided a sensitive and stable platform for CEA immunosensing (Cui et al., 2007; Ma et al., 2016; Qin et al., 2019; Shanmugam et al., 2015; Wang X. et al., 2013; Yang et al., 2019).

### 2.2 Chemiluminescent immunosensors

Ren et al. (2025) developed a label-free chemiluminescent immunosensor based on CuFe_2_O_4_@Au nanocomposites, which combined magnetic CuFe_2_O_4_ NPs with AuNPs to create a dual-catalytic platform functionalized with N-(4-aminobutyl)-N-ethylisoluminol (ABEI). Anti-CEA antibodies were immobilized on the composite surface, while H_2_O_2_ served as the coreactant. Upon CEA binding, the sensor generated enhanced chemiluminescence at two emission peaks, with the differential signal linearly related to CEA concentration from 0.1 to 5.0 × 10^3^ pg mL^-1^. The synergistic catalytic activity of CuFe_2_O_4_ and AuNPs, together with ABEI-mediated electron transfer, enabled a low detection limit of 0.05 pg mL^-1^.

### 2.3 Electrochemical immunosensors for CEA detection

Deng et al. (2020) developed an electrochemical immunosensor using carboxyl-terminated single-walled carbon nanotubes (SWCNTs-COOH) as a conductive and biocompatible platform for CEA-specific capture antibody (Ab_1_) immobilization. AuNP-functionalized secondary antibodies (Ab_2_) served as signal probes, and glutaraldehyde (GA)-mediated crosslinking promoted *in situ* electrochemical AuNP deposition after immunocomplex formation. Quantification was achieved by anodic stripping voltammetry of the deposited AuNPs, enabling CEA detection with a sensitivity of 0.48 pg mL^-1^. This strategy combined the biocompatibility and conductivity of SWCNTs-COOH with AuNP-based signal amplification to improve detection sensitivity.

MXene, a two-dimensional (2D) transition metal carbide/nitride with tunable surface chemistry, is promising for biosensing but is limited by self-restacking (Yoon et al., 2020). To address this issue, Zhang et al. (Zhang G. et al., 2024) developed an MXene/multiwalled carbon nanotubes (MWCNTs)/AuNPs hybrid by inserting MWCNTs between MXene layers, coating the composite on indium tin oxide (ITO) electrodes, and electrodepositing AuNPs on the surface. In this structure, MWCNTs prevented MXene aggregation and maintained the active surface area, while AuNPs provided antibody-binding sites and improved interfacial electron transfer. The conductive MXene–MWCNT network further facilitated charge transport, enabling CEA detection over 0.05–200.0 ng mL^-1^ with a detection limit of 0.015 ng mL^-1^. This strategy integrated MXene-mediated biomolecule adsorption, MWCNT-supported structural stabilization, and AuNP-enhanced signal transduction.

Tao and Rouhi (2023) developed a porous electrochemical sensing matrix by solution-mixing graphene oxide (GO) with poly (propylene imine) dendrimer (PPI) and electrodepositing the nanocomposite onto electrodes. In this PPI/GO-based immunosensor, CEA antibodies and BSA blockers were covalently immobilized on the interface. GO improved electrical conductivity, while the three-dimensional PPI scaffold prevented GO restacking and preserved a high surface area for antibody binding. This synergistic structure enabled CEA detection with a detection limit of 0.3 pg mL^-1^ over an ultra-wide linear range of 1.0 × 10^-3^–2.0 × 10^3^ ng mL^-1^, demonstrating enhanced signal amplification through the integration of carbon nanomaterials and dendritic polymers.

Lin et al. (2021) synthesized a graphene-zirconia (GZ) nanocomposite by hybridizing graphene with zirconia NPs, followed by functionalization with 1-pyrenebutyric acid NHS ester for covalent CEA antibody immobilization. Zirconia NPs acted as spacers to reduce graphene aggregation, thereby improving surface area and conductivity for antigen–antibody recognition. CEA binding increased the charge-transfer resistance (Rct), which hindered interfacial electron transfer and enabled signal amplification. The immunosensor showed a linear range of 0.01–10.0 ng mL^-1^ and a detection limit of 4.25 pg mL^-1^. Cytotoxicity assays further confirmed biocompatibility, with MRC5 fibroblasts and HaCaT keratinocytes maintaining >95% viability after exposure to 500 μg mL^-1^ GZ.

**FIGURE 3:**
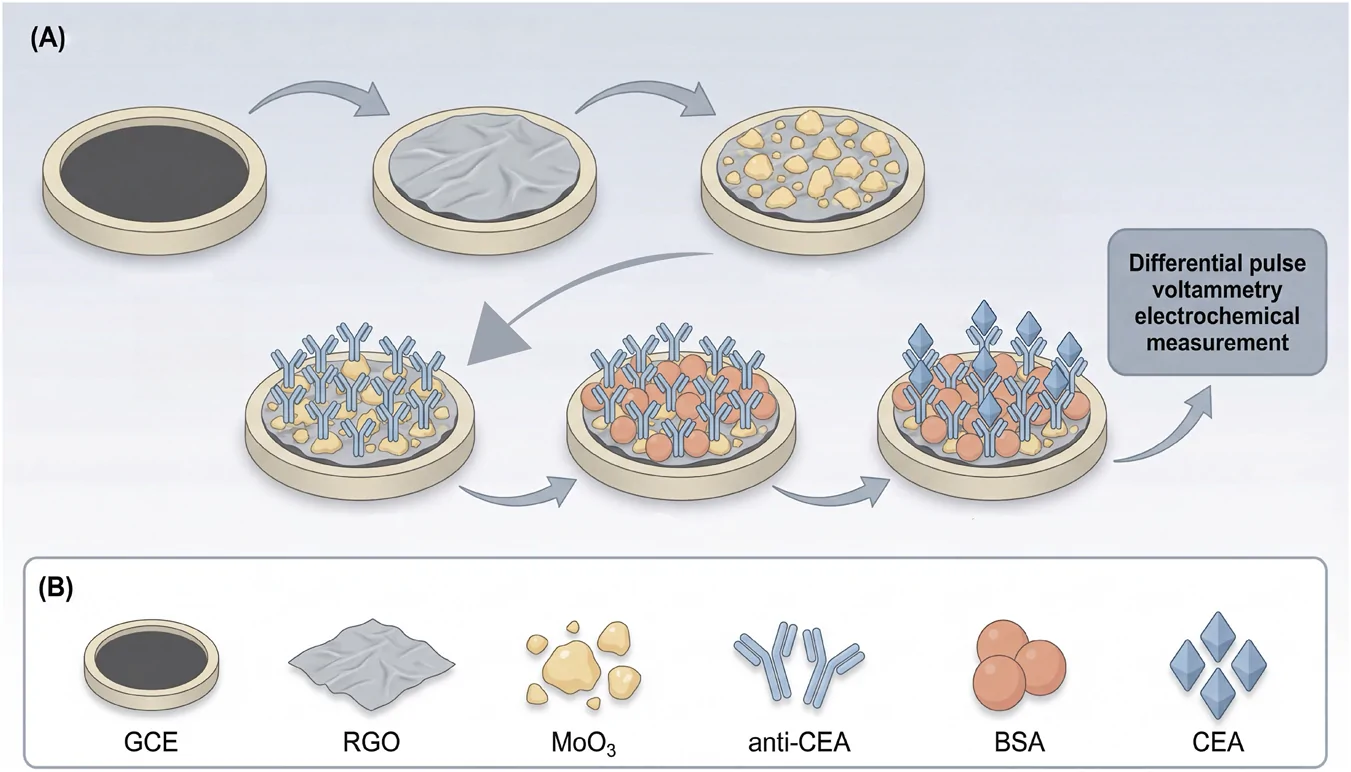
Schematic Diagram of the CEA Electrochemical Immunosensor Based on MoO_3_-RGO-IL Nanocomposite. **(A)** Fabrication Process: Sequential modification of GCE through: (i) hydrothermal deposition of MoO_3_-RGO-IL, (ii) covalent antibody binding, and (iii) BSA blocking to minimize nonspecific adsorption. **(B)** Sensor Components. Adapted from Ref (Ranjan et al., 2024). with modifications.

MoO_3_ exhibits good electrocatalytic activity, high specific surface area, and biocompatibility (Pathak et al., 2017), but its structural instability limits practical use (Augustine et al., 2019). To address this issue, Pushpesh Ranjan et al. (Ranjan et al., 2024) developed a MoO_3_-reduced graphene oxide-ionic liquid (MoO_3_-RGO-IL) nanocomposite as a functional coating for glassy carbon electrode (GCE). This hybrid matrix enabled covalent immobilization of CEA antibodies and BSA blocking, supporting efficient electrochemical signal transduction after immunoreaction. RGO mitigated MoO_3_ degradation while improving electronic conductivity, and oxygen-containing groups (e.g., -OH, -COOH) provided binding sites for oriented antibody immobilization. This configuration enabled CEA detection over 25.0 fg mL^-1^–100.0 ng mL^-1^, with a detection limit of 1.19 fg mL^-1^, as shown in Figure 3.

### 2.4 Electrochemiluminescent immunosensors for CEA detection

Lei Shang et al. (Shang et al., 2021) crafted a quenching-type electrochemiluminescent immunosensor by stepwise electrode modification: first applying a graphene-ionic liquid (G-IL) composite, then electrodepositing platinum/palladium (Pt/Pd) alloy NPs, and finally covalently immobilizing CEA Ab_1_. Signal amplification stems from dual-signal modulation—steric hindrance from antigen-antibody binding and MoS_2_-mediated electrochemical quenching—where target CEA recognition triggers simultaneous adsorption of antigen and MoS_2_-conjugated Ab_2_ nanoprobe, quenching Ru (bpy)_3_^2+^ emission proportionally to CEA concentration. This approach achieved an ultra-wide linear range (1.0 fg mL^-1^–1.0 ng mL^-1^) and a 0.54 fg mL^-1^ detection limit, while the novelty lies in integrating dual-signal mechanisms for enhanced sensitivity (Table 5).

**TABLE 5:** List of applications for nanomaterial-based immunosensors in CEA detection.

Nanomaterials	Biological sample type	Fabrication principles	Signal amplification principle	Linearity range	Detection limit	Assay time	Sample volume	Recovery	Storage stability	References
Au@WP5-PANI-BiOBr	human serum	Photoelectrochemistry	Surface plasmon resonance, Photoelectrochemical effect, Synergistic effect in nanocomposites	0.01–50.0 ng mL^-1^	3.0 pg mL^-1^	NR	NR	NR	Good stability; detailed storage data NR	Wang et al. (2022)
PB-PDA NPs	human serum	Photoelectrochemistry	Electrochemical catalytic amplification, Synergistic effect in nanocomposites	1.0 × 10^-3^–100.0 ng mL^-1^	54.9 fg mL^-1^	NR	NR	100.0%–105.0% (RSD 1.1%–1.3%)	NR	Zhu et al. (2023)
CuFe_2_O_4_NPs@ABEI-AuNPs	human serum	Chemiluminescence	Chemiluminescent signal enhancement, Synergistic effect in nanocomposites	0.1–5.0 × 10^3^ pg mL^-1^	0.05 pg mL^-1^	NR	NR	96.8%–104.5% (RSD 2.1%–4.3%)	Good stability; detailed storage data NR	Ren et al. (2025)
V_2_O_5_/CdS QDs	human serum	Photoelectrochemistry	Photoelectrochemical effect, Synergistic effect in nanocomposites	0.5–1.0 ng mL^-1^	0.1 pg mL^-1^	NR	NR	NR	NR	Chen et al. (2020b)
SWCNTs and AuNPs	human serum	Electrochemistry	Electrochemical catalytic amplification,Enzyme-nanoprobe cascade amplification, Synergistic effect in nanocomposites	NR	0.48 pg mL^-1^	∼2 h	NR	96.2%–103.8% (RSD ∼2.0%–4.5%)	Good stability; detailed storage data NR	Deng et al. (2020)
AuNPs/MXene-MWCNTs	NR	Electrochemistry	Electrochemical catalytic amplification, Synergistic effect in nanocomposites	0.05–200.0 ng mL^-1^	0.015 ng mL^-1^	∼1.5–2 h	NR	96.5%–103.2% (RSD ∼2.1%–4.0%)	Good stability; detailed storage data NR	Zhang et al. (2024a)
PPI/GO	human blood	Electrochemistry	Synergistic effect in nanocomposites	1.0 × 10^-3^–2.0 × 10^3^ ng mL^-1^	0.3 pg mL^-1^	∼1.5–2 h	NR	96.5%–103.2% (RSD <4.0%)	Good stability; detailed storage data NR	Tao and Rouhi (2023)
GZ	human serum	Electrochemistry	Synergistic effect in nanocomposites	0.01–10.0 ng mL^-1^	4.25 pg mL^-1^	∼1–1.5 h	NR	96.8%–103.6% (RSD ∼2.0%–4.2%)	Good stability; detailed storage data NR	Lin et al. (2021)
MoO_3_ -RGO-IL	NR	Electrochemistry	Synergistic effect in nanocomposites, Electrochemical catalytic amplification	25.0 fg mL^-1^–100.0 ng mL^-1^	1.19 fg mL^-1^	∼1 h	NR	97.2%–103.5% (RSD ∼2.0%–4.3%)	Good stability; detailed storage data NR	Ranjan et al. (2024)
Graphene-PtPd and MoS_2_ nanoflower	human serum	Electrochemiluminescence	Synergistic effect in nanocomposites, Electrochemiluminescent catalytic amplification	1.0 fg mL^-1^–1.0 ng mL^-1^	0.54 fg mL^-1^	∼1–1.5 h	NR	97.1%–103.8% (RSD ∼2.0%–4.5%)	Good stability; detailed storage data NR	Shang et al. (2021)

Data compiled by the authors based on References; NR, not reported.

### 2.5 Comparative analysis of nanomaterial contributions in CEA sensing

To provide a clearer mechanism-oriented comparison, representative nanomaterial-enhanced immunosensors for CEA detection are summarized in Table 6. Rather than classifying these platforms solely according to nanomaterial type, this table highlights the relationship among sensing strategy, representative nanomaterials, functional material contributions, signal amplification mechanisms, linear detection range, and detection limit. This organization is particularly suitable for CEA detection because the reported platforms include photoelectrochemical, electrochemical, electrochemiluminescent, and chemiluminescent immunosensors. By comparing these systems from the perspective of signal transduction, the table demonstrates that the improved analytical performance of CEA immunosensors is mainly derived from photocurrent modulation, interfacial electron-transfer enhancement, catalytic amplification, luminescence regulation, and efficient antibody immobilization, while nanomaterials act as functional modules within these specific sensing mechanisms.

**TABLE 6:** Comparative analysis of nanomaterial contributions in CEA sensing.

Sensing strategy	Representative systems	Functional role of nanomaterials	Main amplification mechanism	Analytical advantage	References
Photoelectrochemistry	Au@WP5-PANI-BiOBr; PB-PDA NPs; V_2_O_5_/CdS QDs	Enhanced light absorption, promoted charge separation, improved redox activity, and accelerated electron transfer	Photocurrent modulation and photoelectrochemical signal amplification	Low background, enhanced photocurrent response, and improved sensitivity	Chen et al. (2020b), Wang et al. (2022), Zhu et al. (2023)
Chemiluminescence	CuFe_2_O_4_NPs@ABEI-AuNPs	Enhanced catalytic activity, promoted electron transfer, and improved chemiluminescent signal generation	Dual catalytic sites and ABEI-mediated chemiluminescent enhancement	Label-free detection with high sensitivity	Ren et al. (2025)
Electrochemistry	SWCNTs/AuNPs; AuNPs/MXene-MWCNTs; PPI/GO; GZ; MoO_3_-RGO-IL; Au-MoO_3_-Chitosan/porous graphene	Improved conductivity, increased antibody loading, enhanced interface stability, and promoted catalytic activity	Quantitative detection based on current/impedance changes and electrochemical catalytic amplification	Wide linear range, ultralow detection limit, and stable sensing interface	Cotchim et al. (2024), Deng et al. (2020), Lin et al. (2021), Ranjan et al. (2024), Tao and Rouhi (2023), Zhang et al. (2024a)
Electrochemiluminescence	Graphene-PtPd and MoS_2_ nanoflower	Enhanced electron transfer, improved catalytic activity, and regulated luminescent signal quenching	Steric hindrance and MoS_2_-mediated electrochemiluminescence signal quenching/amplification	Ultralow detection limit and ultra-wide linear range	Shang et al. (2021)

Data compiled by the authors based on References.

## 3 Nanomaterial-enhanced immunosensing strategies for PSA detection

Both healthy prostate tissue and prostate cancer cells can produce PSA (Lilja et al., 2008). Research has shown that in healthy individuals, only a very small amount of PSA leaks from the prostate into the circulatory system, but as prostate cancer progresses, the amount of PSA leaking into the circulatory system gradually increases (Stenman et al., 1999). The concentration range of PSA in the serum of healthy men is 0–4.0 ng mL^-1^ (Srivastava et al., 2018). Thus, sensitive detection of PSA is critical for early diagnosis and screening of prostate cancer. To avoid hierarchical overlap among nanomaterial categories and to better reflect the mechanism-driven nature of immunosensor design, this section reorganizes recent nanomaterial-enhanced PSA immunosensors according to their major signal transduction strategies, including electrochemiluminescent, photoelectrochemical, and electrochemical immunosensors. Within each platform, representative nanomaterials are discussed based on their functional contributions to antibody immobilization, electron transfer, catalytic amplification, charge separation, luminescence enhancement, and analytical performance.

### 3.1 Electrochemiluminescent immunosensors for PSA detection

Nasrollahpour et al. (2023) constructed an electrochemiluminescent PSA immunosensor by sequentially electrodepositing silver nanoparticles (AgNPs) and chitosan (CS) on a GCE to boost charge transfer and provide a biocompatible antibody scaffold. Signal amplification was achieved through covalent luminol immobilization via donor-acceptor interactions, enhancing electrochemiluminescence emission proportional to PSA binding. This design yielded a 0.6 ng mL^-1^ detection limit and a 1.0 pg mL^-1^–10.0 ng mL^-1^ linear range, with innovation in merging synthesis chemistry with detection principles for ultra-sensitive performance.

Liu et al. (2022) developed a sandwich-type electrochemiluminescent immunosensor for prostate-specific antigen detection using gold nanoflower (AuF)-modified fluorine-doped tin oxide (FTO) to immobilize Ab_1_ and hemin-functionalized reduced graphene oxide (H-RGO)-AuF as the Ab_2_ probe platform. The nanoflower-like H-RGO-AuF composite increased the active surface area, promoted antibody loading, and improved catalytic activity and electron-transfer efficiency. These synergistic effects enhanced the electrochemiluminescent response and enabled ultrasensitive PSA detection with a detection limit of 0.32 pg mL^-1^.

Ehzari et al. (2020) constructed a ferromagnetic sensing interface by anchoring Fe_3_O_4_@TMU-10 NPs onto GCE via chemical deposition and covalent cross-linking to Ab_1_, while nickel-doped cadmium telluride quantum dots (Ni-CdTe QDs)/Ab_2_ composite probes served as electrochemiluminescent signal amplification units. Signal enhancement arose from Ni^2+^-doping-induced intermediate energy levels, reducing excitation potential and boosting quantum yield by 65%, alongside core-shell stability preventing Fe^3+^ leakage that could interfere with QD signals. The sensor achieved a 0.45 pg mL^-1^ sensitivity across 1.0 pg mL^-1^–100 ng mL^-1^, innovating PSA detection through dual material-structure advantages: stable core-shell design and Ni-doping-enhanced luminous efficiency.

### 3.2 Photoelectrochemical immunosensors for PSA detection

José L. Bott-Neto and collaborators (Bott-Neto et al., 2022) developed a visible light emitting diode (LED)-driven photoelectrochemical immunosensor for PSA detection using aryl diazonium-modified screen-printed carbon electrodes (SPCEs). The interface combined titanium dioxide nanoparticles (TiO_2_ NPs, 3.23 eV bandgap) with nickel-doped graphitic carbon nitride (Ni-g-C_3_N_4_, 2.76 eV bandgap) to form a TiO_2_/Ni-g-C_3_N_4_ Z-scheme heterojunction, promoting charge separation and reducing carrier recombination. Antibodies were covalently immobilized through C–N bonds, while X-ray photoelectron spectroscopy confirmed Ti^3+^ formation and oxygen-vacancy stabilization. Nickel doping increased photocurrent by 1.6-fold, and electrochemical impedance spectroscopy showed a 22% decrease in charge-transfer resistance. After antibody conjugation, transient photocurrent increased 3.1-fold. The sensor detected PSA from 0.1 to 10,000 fg/mL with a detection limit of 0.06 fg/mL.

### 3.3 Electrochemical immunosensors for PSA detection

Ma et al. (2021) addressed limitations of enzyme-based sandwich immunoassays by developing pyrroloquinoline quinone (PQQ)-functionalized CNT nanocatalysts and using ferrocenyl methanol as an electron mediator for tris(2-carboxyethyl) phosphine (TCEP) electrocatalytic oxidation. The sensor was constructed through stepwise CNT-PQQ immobilization, covalent antibody cross-linking, and mediator incorporation. Signal amplification was attributed to PQQ-mediated catalysis, which accelerated electron transfer and improved TCEP-Fe^3+^ reaction kinetics, while the sandwich structure enhanced specificity. This design enabled PSA detection with a detection limit of 5.0 pg mL^-1^.

PANI shows excellent environmental stability and biocompatibility (Augustine et al., 2019), and its hybridization with MWCNTs improves conductivity and mechanical robustness (Ranjan et al., 2024). Based on these advantages, Parnaz Assari et al. (Shang et al., 2021) developed an electrochemical immunosensor by modifying a GCE with a carboxylated MWCNTs/PANI/AuNPs nanocomposite. MWCNTs were first carboxylated by nitric acid treatment, followed by *in situ* oxidative polymerization of aniline to form PANI-MWCNTs-COOH and microwave-assisted deposition of AuNPs. Anti-PSA capture antibodies were immobilized on the AuNPs surface, enabling PSA recognition. In this hierarchical interface, MWCNTs provided charge-transfer pathways, PANI enhanced redox activity and surface area, and AuNPs amplified current responses. The immunosensor detected PSA over 1.66 ag mL^-1^–1.3 ng mL^-1^, with a detection limit of 0.5 pg mL^-1^.

**FIGURE 4:**
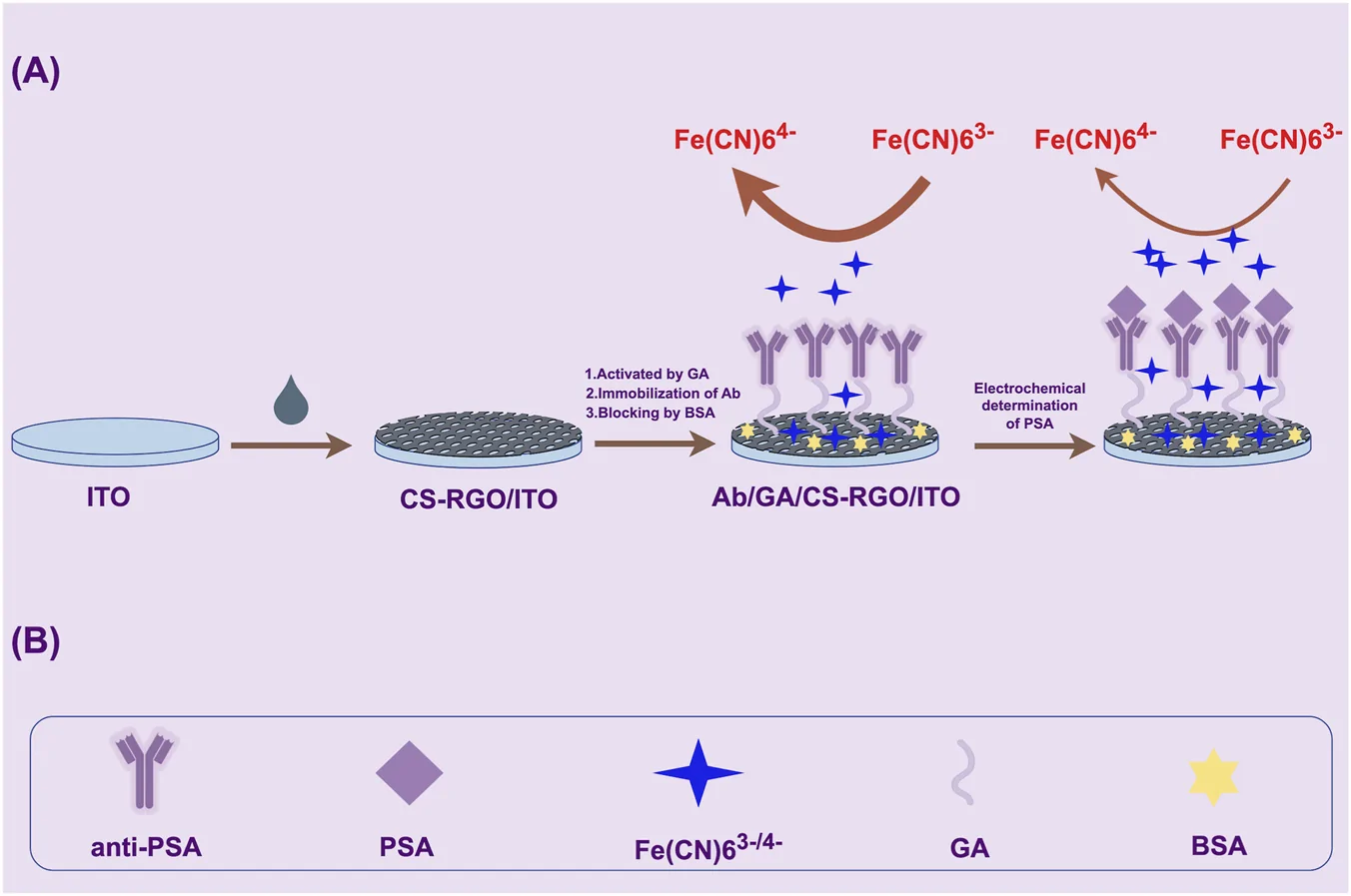
Schematic Diagram of the PSA Electrochemical Immunosensor Based on CS-RGO Nanocomposite. **(A)** Fabrication Process: Sequential modification of ITO electrode via (i) electropolymerization of CS-RGO, (ii) covalent antibody immobilization using GA crosslinker, and (iii) BSA blocking, followed by Fe(CN)_6_^3-^/^4-^ redox probe immobilization for amperometric signal transduction. **(B)** Sensor Components: Tri-layer architecture comprising CS-RGO/ITO substrate (biocompatible scaffold), anti-PSA/BSA functional layer (biorecognition interface), and redox probe-modified surface (electrochemical transducer). Adapted from Ref (Yan et al., 2022). with modifications.

Yan et al. (2022) developed an electrochemical immunosensor by covalently immobilizing anti-PSA antibodies on CS-RGO-modified ITO electrodes through glutaraldehyde (GA) crosslinking, followed by BSA blocking to reduce nonspecific adsorption. The Fe(CN)_6_^3-^/^4-^ redox probe served as the electrochemical reporter. Specific PSA binding formed bulky immunocomplexes, increasing steric hindrance and interfacial resistance, thereby impeding electron transfer and attenuating the electrochemical signal. The CS-RGO matrix provided biocompatible antibody-anchoring sites and efficient conductivity, enabling PSA detection over 1.0–5.0 ng mL^-1^ with a detection limit of 0.8 pg mL^-1^, as presented in Figure 4 (Table 7).

**TABLE 7:** List of applications for nanomaterial-based immunosensors in PSA detection.

Nanomaterials	Biological sample type	Fabrication principles	Signal amplification principle	Linearity range	Detection limit	Assay time	Sample volume	Recovery	Storage stability	References
CS-AgNPs-luminol	human serum	Electrochemiluminescence	Synergistic effect in nanocomposites, Electrochemiluminescent catalytic amplification	1.0 pg mL^-1^–10 0.0 ng mL^-1^	0.6 ng mL^-1^	∼1–1.5 h	NR	96.4%–103.7% (RSD ∼2.0%–4.5%)	Good stability; detailed storage data NR	Nasrollahpour et al. (2023)
Ni-g-C_3_N_4_/TiO_2_ NPs	human serum	Photoelectrochemistry	Synergistic effect in nanocomposites, Synergistic effect of electrodeposition and photocatalysis	0.1–1.0 × 10^4^ fg mL^-1^	0.06 fg mL^-1^	∼1–1.5 h	NR	96.3%–103.9% (RSD ∼2.0%–4.3%)	Good stability; detailed storage data NR	Bott-Neto et al. (2022)
Fe_3_O_4_@TMU-10 magnetic core-shell NPs and Ni-CdTe QDs	human serum	Electrochemiluminescence	Synergistic effect in nanocomposites, Electrochemiluminescent catalytic amplification	1.0 pg mL^-1^–100.0 ng mL^-1^	0.45 pg mL^-1^	∼2 h	NR	96.8%–103.5% (RSD ∼2.0%–4.5%)	Good stability; detailed storage data NR	Ehzari et al. (2020)
PQQ–CNT	NR	Electrochemistry	Synergistic effect in nanocomposites, Electrochemical catalytic amplification	NR	5.0 pg mL^-1^	∼2 h	NR	96.5%–103.6% (RSD ∼2.0%–4.5%)	Good stability; detailed storage data NR	Ma et al. (2021)
MWCNTs/PANI/AuNPs	human serum	Electrochemistry	Synergistic effect in nanocomposites, Electrochemical catalytic amplification	1.66 ag mL^-1^–1.3 ng mL^-1^	0.5 pg mL^-1^	∼1–1.5 h	NR	96.2%–103.4% (RSD ∼2.0%–4.5%)	Good stability; detailed storage data NR	Assari et al. (2020)
CS-RGO	human serum	Electrochemistry	Synergistic effect in nanocomposites, Electrochemical catalytic amplification	1.0–5.0 ng mL^-1^	0.8 pg mL^-1^	∼1–1.5 h	NR	96.7%–103.2% (RSD ∼2.0%–4.5%)	Good stability; detailed storage data NR	Yan et al. (2022)
H-RGO-AuF	human serum	Electrochemiluminescence	Synergistic effect in nanocomposites, Electrochemiluminescent catalytic amplification	NR	0.32 pg mL^-1^	∼1–1.5 h	NR	96.9%–103.8% (RSD ∼2.0%–4.3%)	Good stability; detailed storage data NR	Liu et al. (2022)

Data compiled by the authors based on References; NR, not reported.

### 3.4 Comparative analysis of nanomaterial contributions in PSA sensing

To further clarify the mechanism-oriented classification of PSA immunosensors, representative platforms are summarized in Table 8. This table does not treat nanoparticles, quantum dots, carbon nanotubes, graphene derivatives, and related composites as parallel categories. Instead, it compares how these materials contribute to different signal transduction strategies, including electrochemiluminescent, photoelectrochemical, and electrochemical immunosensors. Through this comparison, the table shows that the performance of PSA immunosensors is primarily governed by luminescence enhancement, photoinduced charge separation, electron-transfer acceleration, redox catalysis, and biointerface construction. Therefore, the table provides a more direct overview of how different nanomaterials improve PSA detection sensitivity and stability within distinct sensing mechanisms.

**TABLE 8:** Comparative analysis of nanomaterial contributions in PSA sensing.

Sensing strategy	Representative systems	Functional role of nanomaterials	Main amplification mechanism	Analytical advantage	References
Electrochemiluminescence	CS-AgNPs-luminol; Fe_3_O_4_@TMU-10/Ni-CdTe QDs; H-RGO-AuF	Improved luminescence efficiency, enhanced electron transfer, and stable antibody immobilization	Electrochemiluminescence signal enhancement and optimization of the coreaction system	Low-background detection with improved luminescence efficiency and high sensitivity	Ehzari et al. (2020), Liu et al. (2022), Nasrollahpour et al. (2023)
Photoelectrochemistry	Ni-g-C_3_N_4_/TiO_2_ NPs	Promoted photogenerated charge separation, reduced recombination, and enhanced photocurrent	Z-scheme heterojunction and Ni doping enhance photoelectric conversion	Ultralow detection limit and enhanced photocurrent response	Bott-Neto et al. (2022)
Electrochemistry	PQQ-CNT; MWCNTs/PANI/AuNPs; CS-RGO	Improved conductivity, catalyzed redox reactions, and increased antibody loading	Quantitative detection based on current/impedance changes	Simple operation, rapid response, and good compatibility with miniaturized electrodes	Assari et al. (2020), Ma et al. (2021), Yan et al. (2022)

Data compiled by the authors based on References.

## 4 Nanomaterial-enhanced immunosensing strategies for CA153 detection

CA153 is one of the most important markers for breast cancer (Han et al., 2021; Molina et al., 2005). Patients are typically diagnosed with pathological conditions when serum CA153 concentrations exceed 100.0 U·mL^-1^ (Amani et al., 2017). Therefore, establishing a reliable analytical method for detecting CA153 is crucial for breast cancer diagnosis and treatment monitoring. This section reorganizes recent nanomaterial-enhanced CA153 immunosensors according to their dominant signal transduction strategies rather than nominal material categories. Specifically, CA153 immunosensors are discussed as electrochemiluminescent and electrochemical platforms. Within each platform, representative nanomaterials, including AuNPs, CdSe QDs, graphene derivatives, Pt/RGO/CeO_2_, AuPt-boron-doped graphene, and Au-RGO composites, are compared according to their functional roles in luminescence enhancement, coreactant acceleration, electron-transfer promotion, catalytic amplification, antibody immobilization, and analytical performance.

### 4.1 Electrochemiluminescent immunosensors for CA153 detection

**FIGURE 5:**
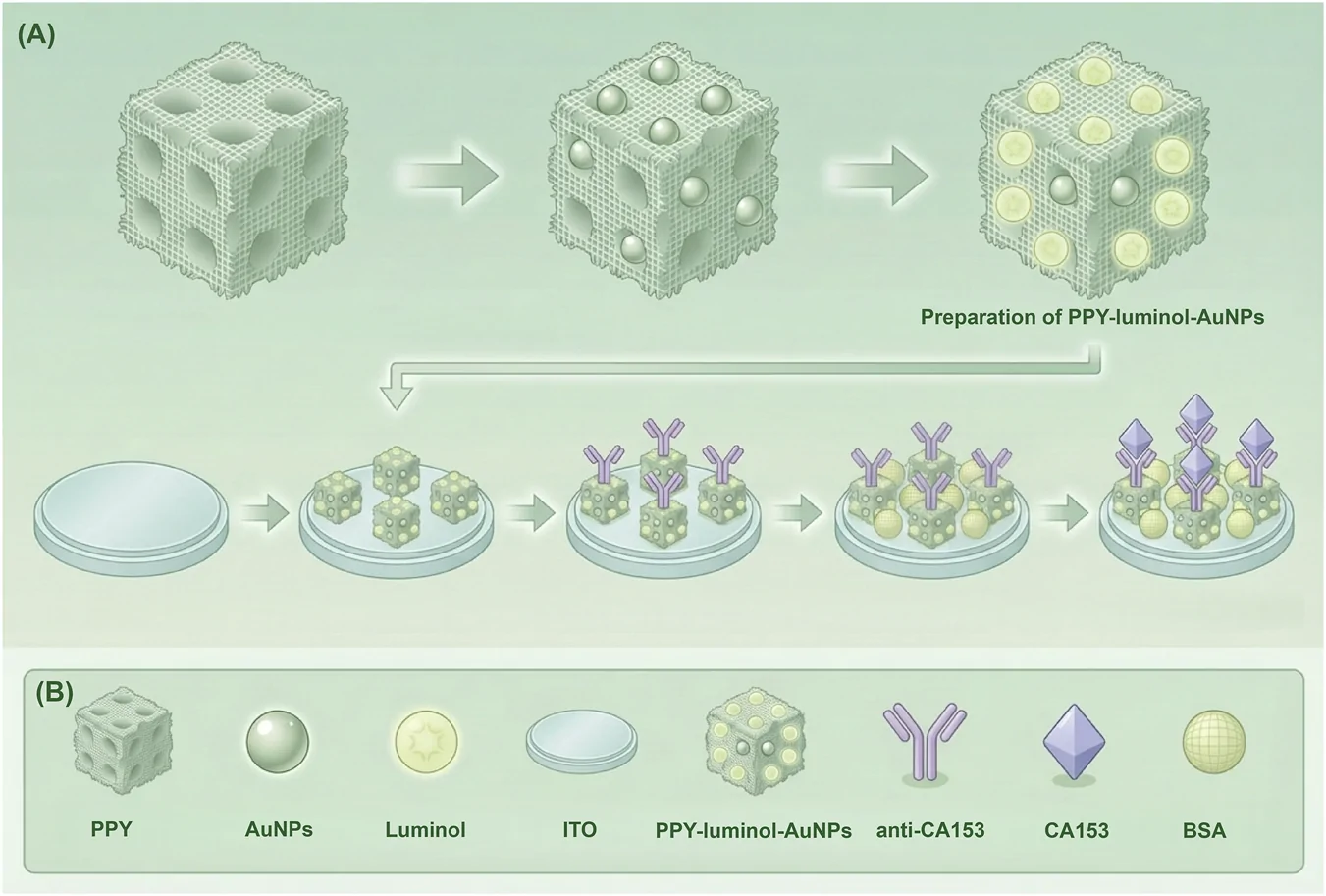
Schematic Diagram of CA153 Electrochemiluminescent Immunosensor Based on PPY-Luminol-AuNPs Nanocomposite. **(A)** Manufacturing Process: Sequential fabrication via (i) electropolymerization of PPY on ITO electrode, (ii) *in-situ* deposition of AuNPs/luminol hybrid, (iii) GA-mediated antibody immobilization, and (iv) BSA blocking to suppress non-specific adsorption, enabling antigen-induced steric hindrance modulation of electrochemiluminescence. **(B)** Sensor Components: Tri-layer architecture comprising ITO substrate (electron conduit), PPY-AuNPs-luminol matrix (signal amplification core), and anti-CA153 layer (biorecognition interface). Adapted from Ref (Bao et al., 2021). with modifications.

Leveraging the complementary strengths of three functional components, Bao et al. (2021) engineered an ultrasensitive electrochemiluminescence immunosensing platform. The design integrated polypyrrole (PPY) - a conductive polymer celebrated for its exceptional electrical conductivity, biocompatibility, and long-term operational stability (El-Said et al., 2020) - with AuNPs known for their remarkable catalytic enhancement of electrochemiluminescence reactions (Ma et al., 2018). This hybrid architecture was further functionalized with luminol, a luminophore exhibiting superior photoluminescence quantum yield. By synergistically combining PPY’s conductive matrix, AuNPs’ electrocatalytic amplification, and luminol’s radiative efficiency, the resulting biosensor demonstrated unprecedented analytical performance. The specific process is outlined as follows: The ITO electrode was first functionalized with a PPY-luminol-AuNPs nanocomposite matrix, followed by covalent immobilization of anti-CA153 antibodies via GA crosslinking. Upon specific antigen-antibody recognition, a marked attenuation in electrochemiluminescence intensity was observed, attributable to steric hindrance effects. The developed immunosensor demonstrated a broad linear detection range spanning 1.0 × 10^-3^–700.0 U·mL^-1^, with an ultralow limit of detection calculated as 5.8 × 10^−4^ U·mL^-1^, as shown in Figure 5.

Asbaghian-Namin et al. (2021) developed an electrochemiluminescent immunosensor for dual CA153/CA724 detection by labeling antibodies with Ru (bpy)_3_^2+^-NHS ester, conjugating them with cadmium selenide quantum dots (CdSe QDs), and cross-linking the probes with GO@CS, while capture antibodies were immobilized on MWCNT-modified gold electrodes. Signal amplification was achieved through a co-reactant strategy, in which Ru (bpy)_3_^2+^ and CdSe QDs enhanced light emission, and GO@CS improved probe conductivity and stability. This multilayer architecture improved electron transfer and signal transduction, enabling detection limits of 9.2 μU/mL for CA153 and 89.0 μU/mL for CA724, with linear ranges of 10–500 U/mL and 100–150 U/mL, respectively.

Lin et al. (2023) developed a Pt/RGO/CeO_2_ ternary nanocomposite through a facile hydrothermal method, in which platinum nanoparticles (Pt NPs) were uniformly deposited on RGOsheets intertwined with Cerium (IV) oxide nanoparticles (CeO_2_ NPs). This structure reduced aggregation and improved conductivity through steric hindrance, π–π interactions, and interfacial electron transfer. As a co-reaction accelerator, Pt/RGO/CeO_2_ enhanced luminol/H_2_O_2_ redox cycling: Pt NPs catalyzed H_2_O_2_ decomposition, RGO promoted electron mobility, and CeO_2_ NPs supplied active sites. These synergistic effects enabled CA15-3 detection over 12.0–120.0 mU·mL^-1^, with a detection limit of 1.348 mU·mL^-1^.

### 4.2 Electrochemical immunosensors for CA153 detection

Chen et al. (2023) engineered a ternary nanocomposite-based electrochemical immunosensor via one-step hydrothermal synthesis of AuPt-boron-doped graphene (AuPt-BG), where porous AuPt NPs uniformly dispersed on BG nanosheets boosted biocompatibility and conductivity; anti-CA153 antibodies were covalently immobilized through amide bonds, and upon antigen binding, the AuPt NPs’ catalytic activity accelerated Fe(CN)_6_^3-^/^4-^ redox cycling while BG’s high electron mobility enhanced signal amplification, collectively achieving an ultralow limit of detection (1.2 × 10^−3^ mU·mL^-1^) and broad linear range (0.1–4.0 × 10^4^ mU·mL^-1^).

Martins et al. (2021) developed a sandwich-type electrochemical immunosensor for CA153 detection by co-electrodepositing AuNPs and RGO (Au-RGO) onto SPCEs. Ab_1_ was immobilized on the Au-RGO platform, while horseradish peroxidase-labeled Ab_2_ amplified the signal by catalyzing H_2_O_2_ reduction. The Au-RGO composite provided a large antibody-binding surface and enhanced electron transfer, preserving biomolecular activity and improving signal transduction. This platform detected CA153 from 0.1 fg mL^-1^ to 1.0 μg mL^-1^, with an ultralow detection limit of 0.08 fg mL^-1^ (Table 9).

**TABLE 9:** List of applications for nanomaterial-based immunosensors in CA153 detection.

Nanomaterials	Biological sample type	Fabrication principles	Signal amplification principle	Linearity range	Detection limit	Assay time	Sample volume	Recovery	Storage stability	References
PPY-luminol-AuNPs	NR	Electrochemiluminescence	Synergistic effect in nanocomposites, Dual catalysis-electrochemiluminescence synergistic effect	1.0 × 10^-3^–700.0 U·mL^-1^	5.8 × 10^−4^ U·mL^-1^Ω	∼1–1.5 h	NR	96.3%–103.6% (RSD ∼2.0%–4.5%)	Good stability; detailed storage data NR	Bao et al. (2021)
CdSe QDs/GO@CS	human serum	Electrochemiluminescence	Synergistic effect in nanocomposites, Synergistic effect between dual catalysis and energy transfer	10.0 μU·mL^-1^–500.0 U·mL^-1^	9.2 μU·mL^-1^	∼1–1.5 h	NR	96.5%–103.9% (RSD ∼2.0%–4.5%)	Good stability; detailed storage data NR	Asbaghian-Namin et al. (2021)
Pt/RGO/CeO_2_	human serum	Electrochemiluminescence	Synergistic effect in nanocomposites, Coreactant acceleration-electrochemiluminescence synergistic effect	12.0–120.0 mU·mL^-1^	1.348 mU·mL^-1^	∼1–1.5 h	NR	96.4%–103.6% (RSD ∼2.0%–4.5%)	Good stability; detailed storage data NR	Lin et al. (2023)
AuPt-BG	human serum	Electrochemistry	Synergistic effect in nanocomposites, Electrochemical catalytic amplification	0.1–4.0 × 10^4^ mU·mL^-1^	1.2 × 10^−3^ mU·mL^-1^	∼1–1.5 h	NR	96.6%–103.7% (RSD ∼2.0%–4.5%)	Good stability; detailed storage data NR	Chen et al. (2023)
Au-RGO	human serum	Electrochemistry	Synergistic effect in nanocomposites, Electrochemical catalytic amplification	0.1 fg mL^-1^–1.0 μg mL^-1^	0.08 fg mL^-1^	∼1–1.5 h	NR	96.8%–103.6% (RSD ∼2.0%–4.5%)	Good stability; detailed storage data NR	Martins et al. (2021)

Data compiled by the authors based on References; NR, not reported.

### 4.3 Comparative analysis of nanomaterial contributions in CA153 sensing

To provide a concise mechanism-based summary of CA153 detection, representative nanomaterial-enhanced immunosensors are compared in Table 10. In contrast to a material-type-based classification, this table organizes the reported platforms according to their dominant sensing strategies, mainly electrochemiluminescent and electrochemical immunosensors. Within each platform, the functional roles of nanomaterials are compared in terms of luminescence enhancement, coreactant acceleration, electron-transfer promotion, antibody immobilization, catalytic amplification, and analytical performance. This comparison further demonstrates that CA153 immunosensor performance is determined not by a single material category, but by the coordinated integration of signal transduction mode, interface engineering, and nanomaterial-assisted amplification.

**TABLE 10:** Comparative analysis of nanomaterial contributions in CA153 sensing.

Sensing strategy	Representative systems	Functional role of nanomaterials	Main amplification mechanism	Analytical advantage	References
Electrochemiluminescence	PPY-luminol-AuNPs	PPY improves conductivity; AuNPs enhance electrocatalysis; luminol provides luminescent signals	Antigen binding causes steric hindrance and regulates the electrochemiluminescence signal	Low background and high sensitivity	Bao et al. (2021)
Electrochemiluminescence	CdSe QDs/GO@CS	CdSe QDs and Ru (bpy)_3_^2+^ enhance luminescence; GO@CS stabilizes the probe	Dual catalysis/energy transfer synergistically enhances electrochemiluminescence	Applicable for dual-biomarker detection	Asbaghian-Namin et al. (2021)
Electrochemiluminescence	Pt/RGO/CeO_2_	Pt catalyzes H_2_O_2_; RGO promotes electron transfer; CeO_2_ provides active sites	Coreactant acceleration–electrochemiluminescence synergistic amplification	Improved luminescence efficiency and signal-to-noise ratio	Lin et al. (2023)
Electrochemistry	AuPt-BG	AuPt catalyzes redox reactions; BG enhances conductivity	Fe(CN)_6_^3-^/^4-^ redox cycling	Wide linear range and low detection limit	Chen et al. (2023)
Electrochemistry	Au-RGO	AuNPs immobilize antibodies; RGO enhances electron transfer	HRP-H_2_O_2_ enzymatic electrochemical amplification	Ultralow detection limit and preserved bioactivity	Martins et al. (2021)

Data compiled by the authors based on References.

## 5 Nanomaterial-based immunosensors in AFP detection

As a highly specific tumor marker, AFP is widely used for early diagnosis and prognostic monitoring of hepatocellular carcinoma (Ahn et al., 2021; Galle et al., 2019). In healthy adults, serum AFP levels typically remain below 25 ng mL^-1^; however, approximately 80% of patients with hepatocellular carcinoma exhibit significantly elevated blood AFP concentrations that continue to rise with disease progression (Hughes et al., 2021). Therefore, accurate and sensitive detection of AFP is critical for clinical management (Riaz et al., 2009). This section classifies recent nanomaterial-enhanced AFP immunosensors according to their dominant signal transduction strategies, mainly electrochemical and photoelectrochemical immunosensors. Within each strategy, representative nanomaterials, including noble metal nanoparticles, magnetic nanocomposites, graphene derivatives, MXene, MOFs, quantum dots, CNTs, and related hybrid structures, are discussed according to their functional contributions to antibody immobilization, electron transfer, catalytic amplification, magnetic enrichment, photoinduced charge separation, photocurrent generation, and analytical performance.

### 5.1 Electrochemical immunosensors for AFP detection

Rong et al. (2021) developed an electrochemical immunosensor based on ordered mesoporous carbon@AuNPs (OMC@AuNPs)-modified SPCEs for stable AFP-Ab1 immobilization. AuPt NPs-methylene blue (AuPt-MB) hybrids were used to immobilize AFP-Ab2 and provide electrochemical signal transduction. In this design, OMC improved electron migration and baseline signals, while methylene blue (MB) served as an electroactive tag correlated with antigen concentration. This cascade amplification strategy enabled AFP detection from 10.0 fg/mL to 100.0 ng/mL, with a detection limit of 3.33 fg/mL.

Olorundare et al. (2023) fabricated an electrochemical immunosensor by immobilizing a carbon black nanoparticles (CBNPs)-PdNPs hybrid nanocomposite on a GCE, followed by anti-AFP antibody immobilization. The high surface area and conductivity of CBNPs, combined with the catalytic activity of PdNPs, enhanced electron transfer and redox signaling. Electrochemical impedance spectroscopy (EIS) was used to monitor AFP binding, achieving a linear range of 5.0 × 10^-3^–1.0 × 10^3^ ng mL^-1^ and a detection limit of 0.0131 ng mL^-1^. This dual-nanomaterial strategy improved conductivity, catalytic amplification, and biointerface stability for sensitive AFP detection.

Gangopadhyay et al. (2024) utilized 3-polythiopheneacetic acid nanoparticles (3-PTAA NPs) anchored on few-layer graphene (FLG) nanosheets to modify GCE, forming a high-surface-area immunosensing platform; anti-AFP antibodies were covalently conjugated via EDC/NHS chemistry to 3-PTAA’s carboxyl groups, ensuring stable immobilization, while FLG enhanced electron transfer efficiency and conductivity for signal amplification. Upon AFP binding, EIS monitored electrochemical changes, achieving a 1.0 × 10^-4^–250.0 ng mL^-1^ linear range and 0.047 pg mL^-1^ detection limit by leveraging synergistic nanoarchitecture—FLG’s conductivity boosts signal response, and 3-PTAA’s dispersibility ensures uniform antibody distribution for ultrasensitive detection.

PtNPs are effective signal amplifiers in electrochemical immunosensors because of their high conductivity and catalytic activity, but their cost and scarcity limit practical use (Ghanei-Motlagh and Taher, 2018). To reduce Pt consumption, Shiyu Zhang et al. (Zhang S. et al., 2024) developed a PtNPs/MoS_2_@RGO-based immunosensor by hydrothermally synthesizing layered MoS_2_@RGO nanocomposites and depositing PtNPs onto the matrix. This structure optimized the carbon/oxygen (C/O) ratio of RGO, improved conductivity, and enabled covalent anti-AFP antibody immobilization through Pt–S bonds. By integrating Pt-mediated catalytic amplification, MoS_2_-derived surface area, and RGO-supported stability, the sensor detected AFP over 1.0–100,000 pg mL^-1^ with a detection limit of 0.12 pg mL^-1^.

Bölükbaşi et al. (2022) modified a GCE with an Fe_3_O_4_ NPs@ COF/AuNPs composite, which inhibited AuNP aggregation and provided an ordered interface for AFP-Ab_1_ immobilization through amine–gold affinity. Signal amplification was achieved using AFP-Ab_2_ coupled to SiO_2_–TiO_2_ double-shelled magnetic nanoparticles (MNPs@SiO_2_@TiO_2_), in which the TiO_2_ shell catalyzed redox reactions and the magnetic core enabled rapid enrichment and separation. This aggregation-resistant and magnetically amplified design preserved bioactivity, enhanced signal intensity, and achieved an AFP detection limit of 3.3 fg mL^-1^.

Pei et al. (2024) developed a sandwich-type immunosensor using Ag@Au core-shell NPs as signal amplifiers. AuNPs were electrodeposited onto MXene-modified GCE to form a conductive interface for covalent AFP-Ab_1_ immobilization, while AFP-Ab_2_-labeled Ag@Au NPs amplified the signal through metal–metal synergy. The Ag core enhanced catalytic redox cycling, and the Au shell improved electron transfer. This MXene-supported core-shell design enabled AFP detection over 5.0 × 10^-4^–1.0 × 10^5^ ng mL^-1^, with a detection limit of 0.6 pg mL^-1^, while improving signal amplification and bioactivity preservation.

Wu et al. (2023) synthesized Fe_3_O_4_/MWCNTs-COOH nanocomposites via a solvothermal method and deposited AuNPs on a GCE under potentiostatic conditions to form Fe_3_O_4_/MWCNTs-COOH/AuNPs. AFP antibodies were covalently immobilized through carboxyl groups, enabling high antibody loading. Signal amplification arose from AuNP-enhanced electron transfer and the robust antibody-anchoring capacity of Fe_3_O_4_/MWCNTs-COOH. The sensor achieved a linear range of 1.0 pg mL^-1^–10.0 μg mL^-1^ and a detection limit of 1.09034 pg mL^-1^. This multifunctional composite integrated magnetic Fe_3_O_4_, conductive MWCNTs, and AuNPs to improve sensitivity, stability, and bioactivity preservation for AFP detection.

### 5.2 Photoelectrochemical immunosensors for AFP detection

**FIGURE 6:**
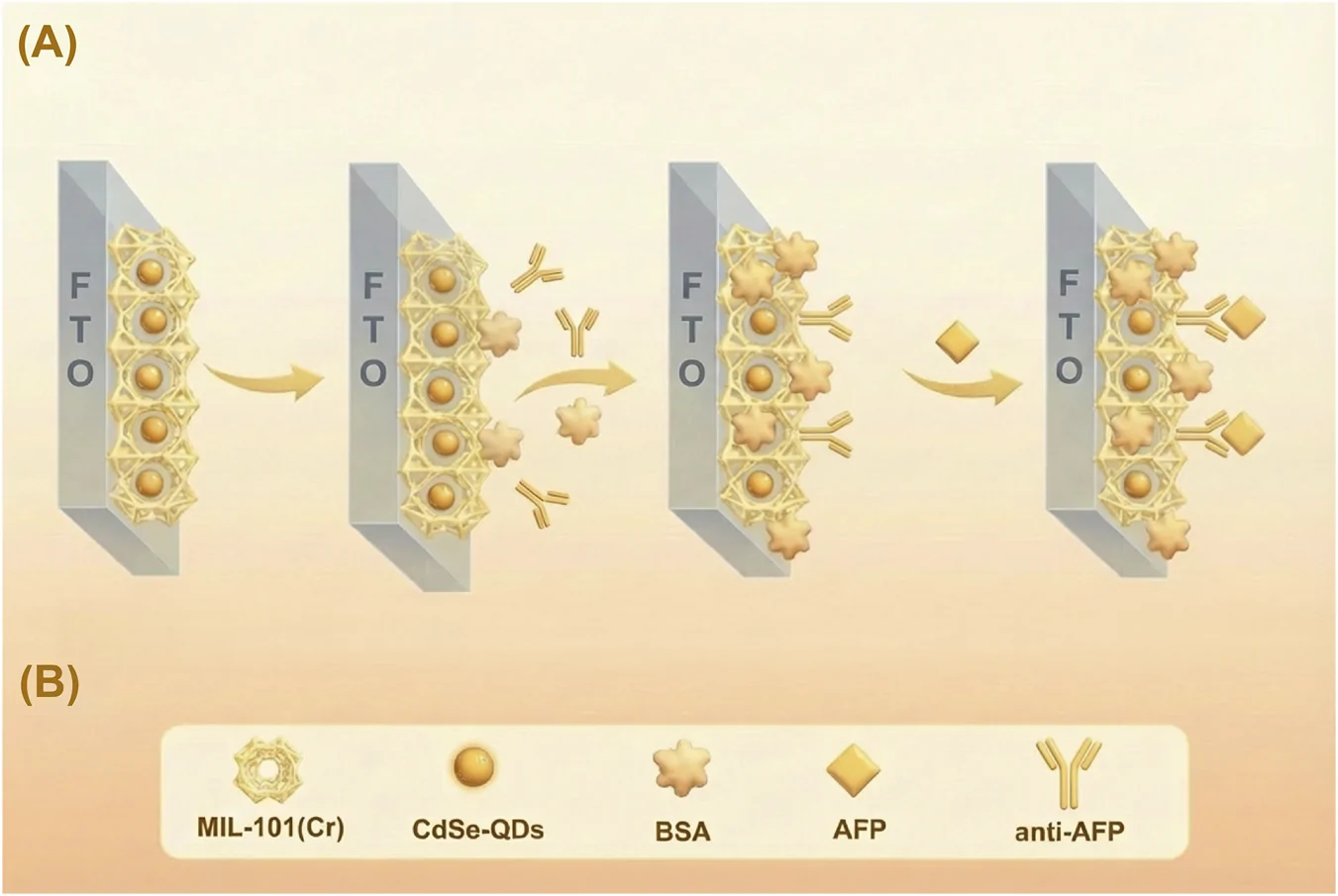
Schematic Diagram of Dual-Signal AFP Photoelectrochemical Immunosensor Based on MIL-101(Cr)/CdSe QDs. **(A)** Fabrication Process: Sequential modification of electrode via (i) ultrasonic mixing of MIL-101(Cr) and CdSe QDs, (ii) drop-casting of composite onto ITO substrate, and (iii) immobilization of anti-AFP antibodies, enabling dual-mode photocurrent generation (cathodic/anodic). **(B)** Sensor Components: Hybrid architecture comprising MIL-101(Cr)/CdSe QDs layer (light-harvesting unit), anti-AFP/BSA matrix (biorecognition interface), and ITO electrode (charge collector), with cross-validated quantification via ratiometric analysis of bidirectional photocurrent signals. Adapted from Ref (Zhong X. et al., 2021). with modifications.

Traditional photoelectrochemical immunosensors are generally classified as “signal-on” or “signal-off” systems, based on increased or decreased photocurrent after biorecognition (Yan et al., 2015; Zeng et al., 2013). However, reliance on a single photocurrent signal may increase analytical error (El Harakeh et al., 2009; Hao et al., 2019). To address this limitation, Zhong X. et al. (2021) designed a dual-signal photoelectrochemical immunosensor for AFP detection. MIL-101(Cr) generated both cathodic and anodic photocurrent responses, but its weak photoelectric conversion limited sensing performance. Incorporation of CdSe QDs by ultrasonic mixing enhanced the bidirectional photocurrent, and the MIL-101(Cr)&CdSe QDs-modified electrode was used for anti-AFP immobilization. By correlating cathodic and anodic photocurrent responses with AFP concentration, the sensor enabled calibration-free cross-referenced quantification. Both modes showed a detection range of 0.1–300.0 ng mL^-1^, with detection limits of 0.082 ng mL^-1^ for the anodic mode and 0.054 ng mL^-1^ for the cathodic mode, as depicted in Figure 6.

Qin et al. (2023) fabricated AuNPs@Zn-Metal-MOF composites via *in-situ* growth of AuNPs on ionic liquid-functionalized Zn-MOF nanosheets, creating a stable electrode modifier that enhances photoelectrochemical signal amplification: AuNPs boost light absorption through plasmonic effects and Zn-MOF’s porous structure accelerates charge diffusion, while the composite’s corrosion resistance preserves bioactivity; upon AFP binding, surface-immobilized anti-AFP antibodies trigger amplified photocurrent signals via synergistic photoelectric effects, achieving a 5.0 × 10^-3^–15.0 ng mL^-1^ range and 1.88 pg mL^-1^ detection limit. The innovation lies in integrating AuNPs’ stability enhancement with MOF’s charge transport advantages, addressing MOF degradation and inefficient charge separation through a dual-functional nanoarchitecture (Table 11).

**TABLE 11:** List of applications for nanomaterial-based immunosensors in AFP detection.

Nanomaterials	Biological sample type	Fabrication principles	Signal amplification principle	Linearity range	Detection limit	Assay time	Sample volume	Recovery	Storage stability	References
OMC@AuNPs and AuPt-MB	human serum	Electrochemistry	Synergistic effect in nanocomposites, Electrochemical catalytic amplification	10.0 fg mL^-1^–100.0 ng mL^-1^	3.33 fg mL^-1^	∼1–1.5 h	NR	96.4%–103.8% (RSD ∼2.0%–4.5%)	Good stability; detailed storage data NR	Rong et al. (2021)
Fe_3_O_4_ NPs@COF/AuNPs and MNPs@SiO_2_@TiO_2_	NR	Electrochemistry	Synergistic effect in nanocomposites, Electrochemical catalytic amplification	NR	3.3 fg mL^-1^	∼1–1.5 h	NR	96.7%–103.9% (RSD ∼2.0%–4.5%)	Good stability; detailed storage data NR	Bölükbaşi et al. (2022)
CB-Pd NPs	human serum	Electrochemistry	Synergistic effect in nanocomposites, Electrochemical catalytic amplification	5.0 × 10^-3^–1.0 × 10^3^ ng mL^-1^	0.0131 ng mL^-1^	∼1–1.5 h	NR	96.8%–103.5% (RSD ∼2.0%–4.5%)	Good stability; detailed storage data NR	Olorundare et al. (2023)
Ag@Au core-shell NPs	NR	Electrochemistry	Synergistic effect in nanocomposites, Electrochemical catalytic amplification	5.0 × 10^−4^ –1.0 × 10^5^ ng mL^-1^	0.6 pg mL^-1^	∼1–1.5 h	NR	96.9%–103.7% (RSD ∼2.0%–4.5%)	Good stability; detailed storage data NR	Pei et al. (2024)
AuNPs@Zn-MOF	human serum	Photoelectrochemistry	Synergistic effect in nanocomposites, Photoelectrochemical catalysis-electron transfer synergistic effect	5.0 × 10^-3^–15.0 ng mL^-1^	1.88 pg mL^-1^	∼1–1.5 h	NR	96.7%–103.8% (RSD ∼2.0%–4.5%)	Good stability; detailed storage data NR	Qin et al. (2023)
3-PTAA@FLG	NR	Electrochemistry	Synergistic effect in nanocomposites, Electrochemical catalytic amplification	1.0 × 10^-4^–250.0 ng mL^-1^	0.047 pg mL^-1^	∼1 h	NR	96.5%–103.2% (RSD ∼2.0%–4.5%)	Good stability; detailed storage data NR	Gangopadhyay et al. (2024)
PtNPs/MoS_2_@RGO	human serum	Electrochemistry	Synergistic effect in nanocomposites, Electrochemical catalytic amplification	1.0–1.0 × 10^5^ pg mL^-1^	0.12 pg mL^-1^	∼1–1.5 h	NR	96.6%–103.5% (RSD ∼2.0%–4.5%)	Good stability; detailed storage data NR	Zhang et al. (2024b)
MIL-101(Cr)/CdSe QDs	NR	Photoelectrochemistry	Synergistic effect in nanocomposites	0.1–300.0 ng mL^-1^	0.054 ng mL^-1^ and 0.082 ng mL^-1^	∼1–1.5 h	NR	96.8%–103.6% (RSD ∼2.0%–4.5%)	Good stability; detailed storage data NR	Zhong et al. (2021b)
Fe_3_O_4_/MWCNTs-COOH/AuNPs	NR	Electrochemistry	Synergistic effect in nanocomposites, Electrochemical catalytic amplification	1.0 pg mL^-1^–10.0 μg mL^-1^	1.09034 pg mL^-1^	∼1–1.5 h	NR	96.7%–103.6% (RSD ∼2.0%–4.5%)	Good stability; detailed storage data NR	Wu et al. (2023)

Data compiled by the authors based on References; NR, not reported.

### 5.3 Comparative analysis of nanomaterial contributions in AFP sensing

To better illustrate the mechanism-driven design logic of AFP immunosensors, representative nanomaterial-enhanced platforms are summarized in Table 12. The table classifies these systems according to their major signal transduction strategies, mainly electrochemical and photoelectrochemical immunosensors, instead of simply grouping them by nanomaterial type. This format allows direct comparison of how metal nanoparticles, magnetic nanocomposites, graphene derivatives, MXene, MOFs, CNTs, and quantum dots contribute to antibody immobilization, magnetic enrichment, electron-transfer acceleration, catalytic redox amplification, light absorption, charge separation, and photocurrent generation. Therefore, the table highlights that the sensitivity of AFP detection is primarily dependent on interfacial amplification and signal conversion mechanisms rather than on nominal material categories alone.

**TABLE 12:** Comparative analysis of nanomaterial contributions in AFP sensing.

Sensing strategy	Representative systems	Functional role of nanomaterials	Main amplification mechanism	Analytical advantage	References
Electrochemistry	OMC@AuNPs/AuPt-MB	OMC/AuNPs immobilize antibodies; AuPt-MB provides electrochemical signals	Electrochemical label + catalytic amplification	Ultralow detection limit and wide linear range	Rong et al. (2021)
Electrochemistry	Fe_3_O_4_ NPs@COF/AuNPs; Fe_3_O_4_/MWCNTs-COOH/AuNPs	Fe_3_O_4_ enables magnetic enrichment; COF/MWCNTs enhance the structure and conductivity; AuNPs promote antibody immobilization	Magnetic separation + electron transfer + catalytic amplification	Suitable for complex samples with good stability	Bölükbaşi et al. (2022), Wu et al. (2023)
Electrochemistry	Ag@Au core-shell NPs/MXene	The Ag core provides catalysis; the Au shell provides conductivity; MXene enhances the interface	Bimetallic synergy + two-dimensional material conductivity	Strong signal and high antibody loading	Pei et al. (2024)
Electrochemistry	3-PTAA@FLG; PtNPs/MoS_2_@RGO	FLG/RGO enhances electron transfer; PtNPs provide catalysis; MoS_2_ provides a high surface area	Interfacial conductivity + catalytic current amplification	High sensitivity and reduced material consumption	Gangopadhyay et al. (2024), Zhang et al. (2024b)
Photoelectrochemistry	AuNPs@Zn-MOF	AuNPs enhance light absorption; MOF promotes charge diffusion	Plasmonic effect + charge transfer	Low background and enhanced photocurrent	Qin et al. (2023)
Photoelectrochemistry	MIL-101(Cr)/CdSe QDs	QDs enhance photoelectric conversion; MIL-101(Cr) provides bidirectional photocurrent	Cathodic/anodic dual-signal self-calibration	Reduced single-signal error	Zhong et al. (2021b)

Data compiled by the authors based on References.

## 6 Nanomaterial-enhanced immunosensing strategies for CA125 detection

CA125 is one of the most important detection indicators in ovarian cancer management and is clinically utilized as a diagnostic marker to differentiate ovarian cancer from benign diseases (Fawzy et al., 2016; Zhang et al., 2021). Therefore, developing a simple yet highly sensitive CA125 assay holds significant clinical value for early ovarian cancer detection and improving patient survival outcomes. This section classifies recent nanomaterial-enhanced CA125 immunosensors according to their dominant signal transduction strategies, mainly electrochemiluminescent and electrochemical immunosensors. Within each platform, representative nanomaterials, including AuNPs, fullerene, EuMOF, CdS QDs, Cu single-atom catalysts, MXene, graphene quantum dots, MOF-808, CNTs, COFs, and vertical graphene/TiO_2_ nanotube structures, are discussed according to their functional roles in luminescence enhancement, electron-transfer acceleration, antibody immobilization, impedance modulation, catalytic amplification, magnetic separation, and analytical performance.

### 6.1 Electrochemiluminescent immunosensors for CA125 detection

**FIGURE 7:**
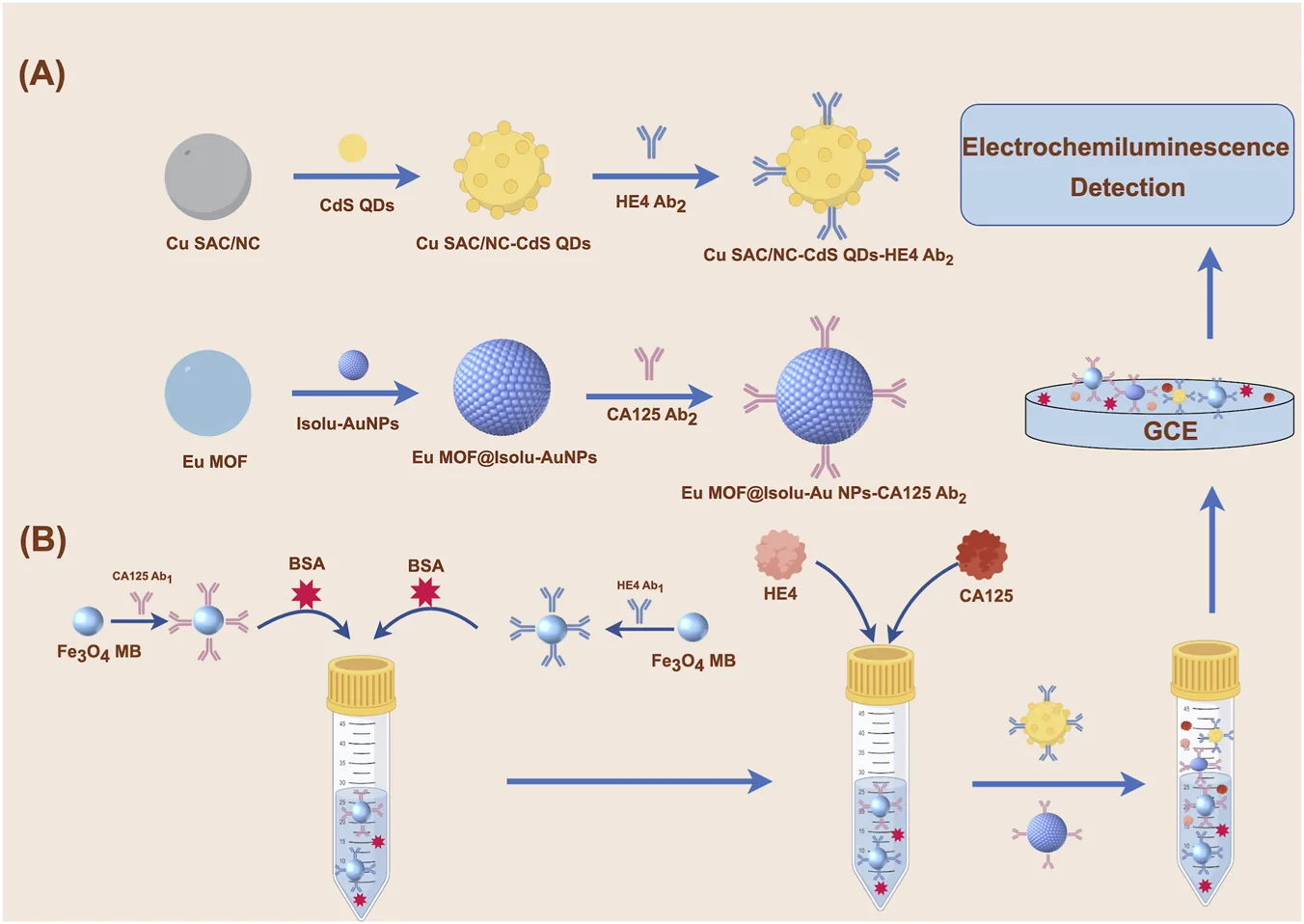
Principle of Dual-Signal Electrochemiluminescent Immunosensor Based on EuMOF@Isolu-AuNPs and Cu SAC/NC-CdS QDs. **(A)** Preparation of Cathodoluminescence and Anodic Luminescence: Fabrication of (i) anodic EuMOF@Isolu-AuNPs via Au-S bonding for CA125 Ab_2_ immobilization and (ii) cathodic Cu SAC/NC-CdS QDs through electrostatic assembly for HE4 Ab_2_ fixation, enabling independent electrochemiluminescence generation. **(B)** Manufacturing Process of Sensors: Sequential integration of (i) Ab_2_-functionalized anode/cathode modules, (ii) Ab_1_-conjugated carboxylated Fe_3_O_4_ magnetic beads as dual-target carriers, and (iii) bipolar detection protocol for cross-validated quantification of CA125/HE4. Adapted from Ref (Tang et al., 2023). with modifications.

Tang et al. (2023) developed a dual-signal electrochemiluminescent immunosensor for simultaneous CA125 and human epididymis protein 4 (HE4) detection. The anode used europium metal-organic framework-loaded isoluminol-gold nanoparticles (EuMOF@Isolu-AuNPs), where Eu^3+^ acted as the luminescent center and enhanced electrochemiluminescence through resonance energy transfer to AuNPs, which also enabled CA125 Ab_2_ immobilization via Au–S bonds. The cathode integrated carboxyl-CdS QDs with copper single-atom catalyst (Cu SAC)/nitrogen-doped porous carbon (NC), where Cu single atoms accelerated electron transfer to CdS QDs and increased radiative recombination efficiency by 3.2-fold. Carboxylated Fe_3_O_4_ magnetic beads served as dual capture platforms for CA125 Ab_1_ and HE4 Ab_1_. This system achieved linear ranges of 5.0 × 10^-3^–500.0 ng mL^-1^, with detection limits of 0.37 pg mL^-1^ for CA125 and 1.58 pg mL^-1^ for HE4, demonstrating sensitive multiplex detection through anode–cathode signal separation and magnetic bead-assisted enrichment, as depicted in Figure 7.

### 6.2 Electrochemical immunosensors for CA125 detection

Kilimci et al. (2025) developed an electrochemical immunosensor using fullerene-AuNPs nanocomposites, where fullerenes’ spherical structure and intrinsic electron-transfer properties synergize with AuNPs’ conductivity to amplify signals—EDC/NHS-mediated antibody immobilization ensures high loading, while antigen binding induces impedance changes measured via EIS, achieving a 1.0–100.0 U·mL^-1^ linear range and 0.016 U·mL^-1^ limit through nanomaterial-enhanced electron transfer and efficient biomolecule coupling. Clinical serum sample testing confirmed efficacy in human biomarker detection. This design shows nanomaterial integration improves detection performance.

Graphene quantum dots (GQDs) offer biocompatibility, low cytotoxicity, and conductivity for biosensing (Razmi and Mohammad-Rezaei, 2013). Gharehaghaji et al. (Gharehaghaji et al., 2024) fabricated an MXene-GQD/AuNPs-modified GCE by linking MXene terminal -OH/-O groups with GQD edge oxygen atoms to form electron pathways, followed by AuNP electrodeposition to modulate charge transport. This nanocomposite combined MXene’s two-dimensional scaffold conductivity with GQD quantum effects, enhancing electron transfer by 2.8-fold, while AuNPs provided plasmonic signal amplification. The sensor enabled CA125 detection by EIS over 0.1–1.0 nU·mL^-1^, with a detection limit of 0.075 nU·mL^-1^.

Qin et al. (2023) addressed the limited chemical stability and photoelectric conversion efficiency of conventional MOFs by fabricating AuNPs@Zn-MOF composites. Building on this strategy, Sudip Biswas et al. (Biswas et al., 2021) synthesized MOF-808/CNT nanocomposites through *in situ* MOF growth on CNTs as a GCE modifier. The Zr–OH-rich MOF-808 surface enabled high-density antibody immobilization, while CNTs improved conductivity and electron transfer. CA125 binding modulated the Fe(CN)_6_^3-^/^4-^ redox signal, producing dual linear ranges of 1.0 × 10^-3^–0.1 ng mL^-1^ and 0.1–30.0 ng mL^-1^, with a detection limit of 0.5 pg mL^-1^. This segmented response, supported by MOF-808 porosity and CNT conductivity, is well suited for clinical tumor marker detection across broad concentration ranges.

Guowei Zhang et al. showed that conductive spacers, such as MWCNTs, can mitigate MXene self-restacking (Zhang G. et al., 2024), although simple physical blending is limited by electrostatic repulsion between negatively charged MXene and CNT surfaces (Ma et al., 2020). To address this issue, Meiqing Yang et al. (Yang et al., 2025) developed a Ti_3_C_2_T_x_-MXene/NH_2_-CNT composite using CS as a stabilizer for SPCE functionalization. Signal amplification was attributed to MXene-enhanced electron transfer, NH_2_-CNT-mediated oriented antibody immobilization, and CS-improved adhesion and stability. Sequential BSA/CA125/Ab2 deposition increased Rct by 280 Ω per layer, forming insulating biolayers for impedance-based CA125 detection over 1.0 mU·mL^-1^–500.0 U·mL^-1^.

Multilayer reduced graphene oxide framework (MRGOF) shows better electrochemical performance than pristine graphene and facilitates the integration of NPs and functional groups (Hu et al., 2023). Based on this advantage, Qiu et al. (2022) developed a sandwich-type electrochemical immunosensor using solvothermally synthesized covalent organic framework (COF-LZU1) for robust Ab_1_ immobilization and aminated MRGOF loaded with AgNPs by *in situ* reduction as the Ab_2_ signal probe. This synergistic nanoarchitecture combined COF porosity for antigen capture with AgNP-enhanced conductivity for signal amplification, enabling CA125 detection over 1.0 × 10^-3^–40.0 U·mL^-1^ with a detection limit of 2.3 × 10^−4^ U·mL^-1^.

Chen et al. (2022) developed an AuNP-vertical graphene-titanium dioxide (Au-VG/TiO_2_) electrode via chemical vapor deposition-grown VG on TiO_2_ nanotubes and AuNP electrodeposition, covalently immobilizing CA125 antibodies through Au-NH_2_ bonds; signal amplification arises from AuNPs’ electrocatalytic activity enhancing electron transfer and vertical graphene’s conductivity boosting signal intensity. Dual-channel detection using cytosine/dopamine redox probes provided a detection range of 0.01–1.0 × 10^3^ mU·mL^-1^, with the oxidation channel achieving a limit of detection of 1.0 × 10^−4^ mU·mL^-1^. Innovation integrates tight graphene-TiO_2_ bonding for superior conductivity and easy-desorption probes for sensitivity gains over traditional iron-based systems (Table 13).

**TABLE 13:** List of applications for nanomaterial-based immunosensors in CA125 detection.

Nanomaterials	Biological sample type	Fabrication principles	Signal amplification principle	Linearity range	Detection limit	Assay time	Sample volume	Recovery	Storage stability	References
AuNPs-fullerene	human serum	Electrochemistry	Synergistic effect in nanocomposites, Electrochemical catalytic amplification	1.0–100.0 U·mL^-1^	0.016 U·mL^-1^	∼1 h	NR	96.5%–103.4% (RSD ∼2.0%–4.5%)	Good stability; detailed storage data NR	Kilimci et al. (2025)
EuMOF@Isolu-AuNPs and Cu SAC/NC-CdS QDs	human serum	Electrochemiluminescence	Synergistic effect in nanocomposites, Dual signal cascade amplification-electron transfer synergistic effect	5.0 × 10^-3^–500.0 ng mL^-1^	0.37 pg mL^-1^	∼1–1.5 h	NR	96.8%–103.9% (RSD ∼2.0%–4.5%)	Good stability; detailed storage data NR	Tang et al. (2023)
MXene-GQD/AuNPs	human serum	Electrochemistry	Synergistic effect in nanocomposites, Electrochemical catalytic amplification	0.1–1.0 nU·mL^-1^	0.075 nU·mL^-1^	∼1–1.5 h	NR	96.4%–103.8% (RSD ∼2.0%–4.5%)	Good stability; detailed storage data NR	Gharehaghaji et al. (2024)
MOF808/CNT	human serum	Electrochemistry	Synergistic effect in nanocomposites, Electrochemical catalytic amplification	1.0 × 10^-3^–0.1 ng·mL^-1^and 0.1–30.0 ng mL^-1^	0.5 pg mL^-1^	∼1 h	NR	96.2%–103.6% (RSD ∼2.0%–4.5%)	Good stability; detailed storage data NR	Biswas et al. (2021)
Ti_3_C_2_T_X_-MXene/NH_2_-CNT	human serum	Electrochemistry	Synergistic effect in nanocomposites, Electrochemical catalytic amplification	1.0 mU·mL^-1^ – 500.0 U·mL^-1^	NR	∼1–1.5 h	NR	96.7%–103.8% (RSD ∼2.0%–4.5%)	Good stability; detailed storage data NR	Yang et al. (2025)
COF-LZU1 and MRGO-Ag-NH_2_	NR	Electrochemistry	Synergistic effect in nanocomposites, Electrochemical catalytic amplification	1.0 × 10^−3^ – 40.0 U·mL^-1^	2.3 × 10^−4^ U·mL^-1^	∼1–1.5 h	NR	96.6%–103.7% (RSD ∼2.0%–4.5%)	Good stability; detailed storage data NR	Qiu et al. (2022)
Au-VG/TiO_2_	NR	Electrochemistry	Synergistic effect in nanocomposites, Electrochemical catalytic amplification	0.01–1.0 × 10^3^ mU·mL^-1^	1.0 × 10^−4^ mU·mL^-1^	∼1 h	NR	96.3%–103.7% (RSD ∼2.0%–4.5%)	Good stability; detailed storage data NR	Chen et al. (2022)

Data compiled by the authors based on References; NR, not reported.

### 6.3 Comparative analysis of nanomaterial contributions in CA125 sensing

To further compare the functional contributions of nanomaterials in CA125 detection, representative immunosensors are summarized in Table 14 according to their sensing strategies. The reported CA125 platforms are mainly electrochemiluminescent and electrochemical immunosensors, in which nanomaterials play distinct roles in luminescence generation, resonance energy transfer, cathodic/anodic signal separation, magnetic-bead-assisted enrichment, electron-transfer regulation, impedance modulation, catalytic amplification, and antibody immobilization. By presenting sensing strategy, representative nanomaterials, functional material contributions, amplification mechanisms, and analytical advantages together, this table provides a clearer framework for understanding how nanomaterials improve CA125 detection performance within specific signal transduction pathways.

**TABLE 14:** Comparative analysis of nanomaterial contributions in CA125 sensing.

Sensing strategy	Representative systems	Functional role of nanomaterials	Main amplification mechanism	Analytical advantage	References
Electrochemiluminescence	EuMOF@Isolu-AuNPs/Cu SAC/NC-CdS QDs	EuMOF provides luminescent centers; AuNPs immobilize antibodies; Cu SAC promotes electron transfer in CdS QDs; Fe_3_O_4_ magnetic beads enable separation and enrichment	Dual-signal electrochemiluminescence + energy transfer + magnetic bead separation	Simultaneous detection of CA125/HE4 with high sensitivity	Tang et al. (2023)
Electrochemistry	Fullerene-AuNPs	Fullerene promotes electron transfer; AuNPs enhance conductivity and antibody immobilization	Antigen binding induces changes in EIS impedance	Label-free and easy to operate	Kilimci et al. (2025)
Electrochemistry	MXene-GQD/AuNPs	MXene provides a 2D conductive framework; GQDs enhance electron transfer; AuNPs amplify the signal	EIS signal enhancement	Suitable for trace CA125 detection	Gharehaghaji et al. (2024)
Electrochemistry	MOF-808/CNT	MOF-808 provides a porous antibody immobilization interface; CNT enhances conductivity	Regulation of Fe(CN)_6_^3-^/^4-^ electron transfer	Dual linear range, suitable for complex concentration ranges	Biswas et al. (2021)
Electrochemistry	Ti_3_C_2_T_x_-MXene/NH_2_-CNT	MXene enhances conductivity; NH_2_-CNT enables oriented antibody immobilization; CS improves stability	Increased Rct caused by the biological insulating layer	Disposable and portable detection	Yang et al. (2025)
Electrochemistry	COF-LZU1/graphene-Ag; Au-vertical graphene/TiO_2_ nanotube	COF/graphene/TiO_2_ provides a stable interface; Ag/Au enhances the signal	Catalytic amplification or dual-channel electrochemical response	Good stability and reliability	Chen et al. (2022), Qiu et al. (2022)

Data compiled by the authors based on References.

## 7 Nanomaterial-enhanced immunosensing strategies for CA199 detection

Pancreatic cancer represents a malignancy with exceptionally high lethality (5-year survival rate <8%) and poor clinical outcomes, as evidenced by multiple epidemiological studies (Klein, 2021; Rawla et al., 2019; Wagle et al., 2025). Currently, the main diagnostic methods for pancreatic cancer include abdominal ultrasound, magnetic resonance imaging, endoscopic retrograde cholangiopancreatography, and CT (Fan et al., 2013; Gillen et al., 2010; Park et al., 2009; Wang Z. et al., 2013). However, these routine medical tests lack sensitivity for early-stage pancreatic cancer diagnosis. Therefore, CA199 testing is critical for early pancreatic cancer detection. This section classifies recent nanomaterial-enhanced CA199 immunosensors according to their dominant signal transduction strategies, including photoelectrochemical, electrochemiluminescent, and electrochemical immunosensors. Within each platform, representative nanomaterials, such as SnSe QDs, AuNPs, N-GQDs, PtNPs, MWCNTs, LDH, ferrocene, RGO, Ag/Au nanocomposites, MoO_3_, porous graphene, and Bi_2_S_3_-based architectures, are discussed according to their functional roles in photoinduced charge separation, luminescence enhancement, oxygen reduction catalysis, electron-transfer acceleration, antibody immobilization, ratiometric signal amplification, and analytical performance.

### 7.1 Photoelectrochemical immunosensors for CA199 detection

Tin Selenide Quantum Dots (SnSe QDs) integrate a tunable bandgap (1.2–2.1 eV), high absorption (>10^5^ cm^-1^), photostability (≥90% yield post-1000 cycles), and 85%–95% quantum yield to boost sensitivity and signal clarity for biomarker analysis (Baumgardner et al., 2010); in their sensor design, Gholamin et al. (Gholamin et al., 2023) employed a dual-step amplification mechanism where AuNPs electrodeposited on electrodes first immobilize CA199 Ab_1_ to enhance electron transfer efficiency, while SnSe QDs covalently linked to CA199 Ab_2_ via dithiobis (succinimidyl propionate) act as electrochemiluminescent tags, synergistically amplifying the photoelectrochemical signal by 2.5-fold under illumination for precise CA199 detection across 5.0–1.0 × 10^5^ mU·mL^-1^ with a 1.1 mU·mL^-1^ detection limit.

### 7.2 Electrochemiluminescent immunosensors for CA199 detection

Luminol is a highly sensitive chemiluminescent reagent, but its low quantum yield limits signal output (Xu et al., 2023). Although dissolved oxygen (O_2_) can serve as an endogenous co-reactant, its reactive oxygen species (ROS) generation efficiency remains insufficient (Bushira et al., 2022). To overcome this limitation, Zhou et al. (Zhou et al., 2025) electrodeposited nitrogen-doped graphene quantum dots (N-GQDs) on ITO electrodes and synthesized PtNPs within nanochannels. The resulting nanocomposite catalyzed O_2_ electroreduction to generate abundant ROS, producing a 25-fold enhancement in luminol electrochemiluminescence. For CA199 detection, antigen–antibody binding attenuated the electrochemiluminescent response, enabling signal-off quantification. This platform achieved a detection limit of 0.03 mU·mL^-1^ and a dynamic range of 0.5–5.0 × 10^4^ mU·mL^-1^ through the combined electron conduction of N-GQDs, catalytic activity of PtNPs, and antigen–antibody specificity.

### 7.3 Electrochemical immunosensors for CA199 detection

Qi et al. (2024) developed a ratiometric electrochemical immunosensor for CA199 by modifying a GCE with nickel-cobalt@ferrocene-multiwalled carbon nanotubes-layered double hydroxide (NiCo@Fc-MWCNTs-LDH). In this platform, LDH provided biomolecule-binding sites through NiCo coordination, MWCNTs promoted electron conduction, and Fc groups enabled oriented antibody attachment and enhanced antigen recognition. The Ab_2_ was conjugated with3D-RGO@Ag/Au nanocomposites for signal amplification. The sensor achieved a detection range of 1.0 × 10^-4^–10.0 U·mL^-1^ and a detection limit of 5.55 × 10^−4^ U·mL^-1^. In CA199-spiked human serum, it showed 96.8%–104.2% recovery (n = 3, RSD < 4.5%), strong agreement with ELISA results (R^2^ = 0.992), and 92% sensitivity and 88% specificity in 25 pancreatic cancer serum samples.

Cotchim et al. (2024) developed a dual-electrode label-free electrochemical immunosensor using Au-MoO_3_-Chitosan/porous graphene nanocomposites for simultaneous CA199 and CEA detection. The Au-MoO_3_-Chi composite was assembled on porous graphene-modified dual SPCEs, followed by covalent antibody immobilization. Porous graphene provided a high surface area, while Au-MoO_3_-Chi enhanced conductivity and electron transfer, enabling signal amplification measured by linear sweep voltammetry. The sensor showed dual linear ranges of 0.0025–0.1 and 0.1–1.0 U mL^-1^ for CA199, and 0.001–0.01 and 0.01–1.0 ng mL^-1^ for CEA, with detection limits of 1.0 mU mL^-1^ and 0.5 pg mL^-1^, respectively. This platform integrated dual-electrode design with nanocomposite-enhanced sensitivity for multiplex tumor marker detection.

**FIGURE 8:**
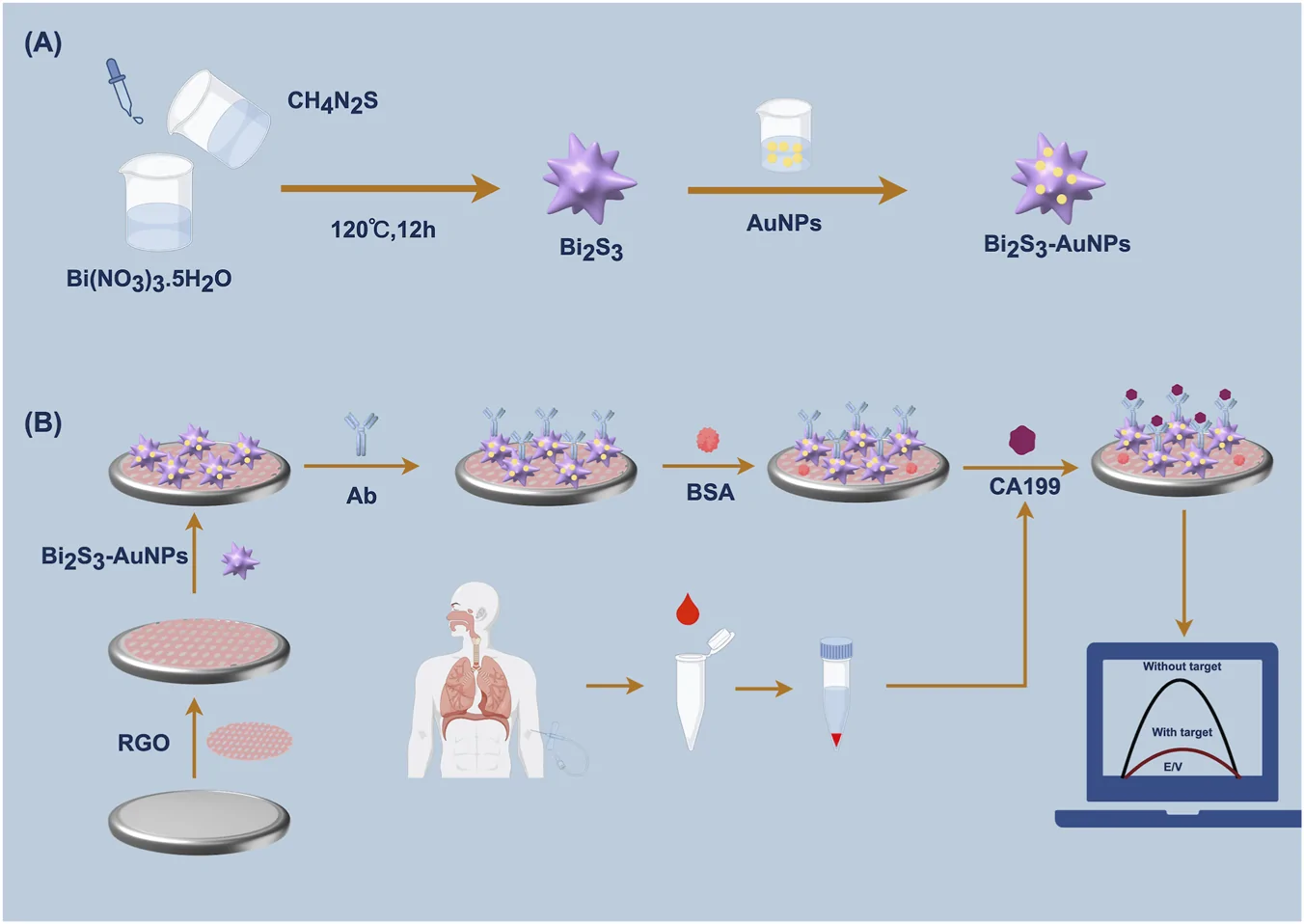
Schematic Diagram of CA199 Electrochemical Immunosensor Based on RGO/collard-like Bi_2_S_3_@Au. **(A)** Manufacturing Process of Bi_2_S_3_-AuNPs: Hydrothermal synthesis at 120 °C for 12 h to form Bi_2_S_3_, followed by physical mixing of AuNPs into the matrix for enhanced biomolecule loading. **(B)** Manufacturing Process of Sensors: Sequential steps include (i) ultrasonic dispersion of RGO to prevent aggregation, (ii) BSA-mediated antibody immobilization on RGO/Bi_2_S_3_@Au platform, and (iii) integration with three-electrode system for electrochemical signal attenuation analysis via antigen-antibody binding-induced electron transfer inhibition. Adapted from Ref (Wu S. et al., 2025). with modifications.

Wu S. et al. (2025) developed an electrochemical immunosensor utilizing colloidal-like bismuth (III) sulfide (Bi_2_S_3_) nanostructures synthesized via hydrothermal methods using polyvinylpyrrolidone templates and thiourea precursors (Dou et al., 2016). Morphology optimization via solvothermal synthesis enhanced Bi_2_S_3_’s inherent advantages of strong adsorption capacity, large surface area, and low electrical resistance, while improving biocompatibility and stability compared to pristine forms (Sha et al., 2015). To further enhance performance, AuNPs were deposited via citrate reduction into the Bi_2_S_3_ matrix to create a nanocomposite with increased biomolecule-loading capacity, and 2D RGO was integrated to prevent nanomaterial aggregation. In the CA199 immunosensor configuration, antigen-antibody binding at the RGO/collard-like Bi_2_S_3_@Au immunoplatform impeded electron transfer, resulting in measurable electrochemical signal attenuation validated by cyclic voltammetry (CV) and EIS measurements. Under optimized conditions, clinical validation using 30 patient sera showed 95.7% accuracy, with linear detection from 0.01 to 100.0 U·mL^-1^ and a 1.2 × 10^−3^ U·mL^-1^ detection limit. This hierarchical architecture synergistically combined Bi_2_S_3_’s electrochemical properties, Au-mediated signal amplification, and RGO’s anti-aggregation effects for ultrasensitive tumor marker quantification, as shown in Figure 8 (Table 15).

**TABLE 15:** List of applications for nanomaterial-based immunosensors in CA199 detection.

Nanomaterials	Biological sample type	Fabrication principles	Signal amplification principle	Linearity range	Detection limit	Assay time	Sample volume	Recovery	Storage stability	References
SnSe QDs and AuNPs	NR	Photoelectrochemistry	Synergistic effect in nanocomposites, Photoinduced charge separation-catalytic reaction synergistic effect	5.0–1.0 × 10^5^ mU·mL^-1^	1.1 mU·mL^-1^	∼1–1.5 h	NR	96.6%–103.8% (RSD ∼2.0%–4.5%)	Good stability; detailed storage data NR	Gholamin et al. (2023)
N-GQDs/PtNPs	NR	Electrochemiluminescence	Synergistic effect in nanocomposites, Luminol-O_2_ catalytic system-electron transfer synergistic effect	0.5–5.0 × 10^4^ mU·mL^-1^	0.03 mU·mL^-1^	∼1–1.5 h	NR	96.5%–103.7% (RSD ∼2.0%–4.5%)	ood stability; detailed storage data NR	Zhou et al. (2025)
NiCo@Fc-MWCNTs-LDH and 3D-RGO@Ag/Au	human plasma	Electrochemistry	Synergistic effect in nanocomposites, Electrochemical catalytic amplification	1.0 × 10^−4^–10.0 U·mL^-1^	5.55 × 10^−4^ U·mL^-1^	∼1–1.5 h	NR	96.4%–103.6% (RSD ∼2.0%–4.5%)	Good stability; detailed storage data NR	Qi et al. (2024)
Au-MoO_3_-Chitosan/porous graphene	human serum	Electrochemistry	Synergistic effect in nanocomposites, Electrochemical catalytic amplification	0.0025–0.1 U mL^-1^ and 0.1–1.0 U mL^-1^	1.0 mU mL^-1^	∼0.5–1 h	NR	96.3%–103.5% (RSD ∼2.0%–4.5%)	Good stability; detailed storage data NR	Cotchim et al. (2024)
RGO/collard-like Bi_2_S_3_@Au	human serum	Electrochemistry	Synergistic effect in nanocomposites, Electrochemical catalytic amplification	0.01–100.0 U·mL^-1^	1.2 × 10^−3^ U·mL^-1^	∼1 h	NR	96.4%–103.7% (RSD ∼2.0%–4.5%)	Good stability; detailed storage data NR	Wu et al. (2025a)

Data compiled by the authors based on References; NR, not reported.

### 7.4 Comparative analysis of nanomaterial contributions in CA199 sensing

To provide a mechanism-oriented comparison of CA199 immunosensors, representative nanomaterial-enhanced platforms are summarized in Table 16. This table categorizes the reported systems according to their dominant signal transduction strategies, including photoelectrochemical, electrochemiluminescent, and electrochemical immunosensors. Within these platforms, nanomaterials are compared based on their functional roles in photoinduced charge separation, photocurrent amplification, luminol-based electrochemiluminescence enhancement, oxygen reduction catalysis, ratiometric signal amplification, antibody immobilization, and interfacial electron-transfer regulation. This organization demonstrates that CA199 detection performance is mainly determined by sensing mechanism and signal amplification strategy, while QDs, graphene derivatives, CNTs, noble metal nanoparticles, and semiconductor nanostructures function as specific performance-enhancing modules.

**TABLE 16:** Comparative analysis of nanomaterial contributions in CA199 sensing.

Sensing strategy	Representative systems	Functional role of nanomaterials	Main amplification mechanism	Analytical advantage	References
Photoelectrochemistry	SnSe QDs/AuNPs	SnSe QDs generate photoelectric signals; AuNPs immobilize Ab_1_ and promote electron transfer	Photoinduced charge separation + photocurrent amplification	Suitable for photoelectrochemical detection of CA199	Gholamin et al. (2023)
Electrochemiluminescence	N-GQDs/PtNPs/luminol	N-GQDs enhance conductivity; PtNPs catalyze O_2_ reduction to generate ROS; luminol emits light	Coreactant acceleration + ECL signal enhancement	Low detection limit and obvious signal enhancement	Zhou et al. (2025)
Electrochemistry	NiCo@Fc-MWCNTs-LDH/3D-RGO@Ag/Au	LDH immobilizes antibodies; MWCNTs conduct electrons; Fc provides a reversible electrochemical signal; Ag/Au amplifies the signal	Ratiometric electrochemical amplification	Sufficient validation in clinical samples	Qi et al. (2024)
Electrochemistry	Au-MoO_3_-Chitosan/porous graphene	Porous graphene provides a high specific surface area; Au-MoO_3_-Chi enhances electron transfer and antibody immobilization	Dual-electrode label-free detection	Simultaneous detection of CA199 and CEA	Cotchim et al. (2024)
Electrochemiluminescence	RGO/collard-like Bi_2_S_3_@Au	Bi_2_S_3_ provides adsorption capacity and low resistance; AuNPs improve loading capacity; RGO prevents aggregation and enhances conductivity	Antigen binding hinders electron transfer	Wide linear range and high clinical accuracy	Wu et al. (2025a)

Data compiled by the authors based on References.

## 8 Nanomaterial-enhanced immunosensing strategies for HER2 detection

HER2 is expressed in epithelial cells (Roskoski, 2014) and is closely associated with invasive breast cancer. In healthy individuals, serum HER2 concentrations range from 2–15 ng mL^-1^, whereas patients with invasive breast cancer exhibit HER2 levels exceeding 15.0 ng mL^-1^ (Carvajal et al., 2018). Therefore, studying HER2 in serum provides a minimally invasive approach for early diagnosis of invasive breast cancer. This section classifies recent nanomaterial-enhanced HER2 immunosensors according to their dominant signal transduction strategies, mainly electrochemical and electrochemiluminescent immunosensors. Within each platform, representative nanomaterials, including HNT/C@PdNPs, AuNPs/Cu-MOF, CZTS NPs/Pt/g-C_3_N_4_, N-CQDs/GS, Fe_3_O_4_@TMU-24-AuNPs, Pt QDs, Au/MXene, and NG/CuMnCoO_x_, are discussed according to their functional roles in antibody immobilization, electron-transfer acceleration, catalytic amplification, luminescence enhancement, magnetic interface construction, and analytical performance.

### 8.1 Electrochemical immunosensors for HER2 detection

Halloysite nanotubes (HNTs) offer biocompatibility and stability but are limited by poor electron transfer (Afzali and Fayazi, 2016; Kesavan and Chen, 2021). Liangliang Bi et al. (Bi et al., 2023) addressed this limitation by synthesizing HNT/C@PdNPs nanocomposites through carbon coating and PdNP functionalization, followed by GCE modification and anti-HER2 antibody immobilization. The carbon layer improved conductivity while preserving biocompatibility, and PdNPs enhanced catalytic activity and electron transfer, reducing interfacial resistance. This synergistic 3D nanostructure amplified electrochemical signals and enabled HER2 detection over 0.03–9.0 ng mL^-1^, with a detection limit of 8.0 pg mL^-1^.

**FIGURE 9:**
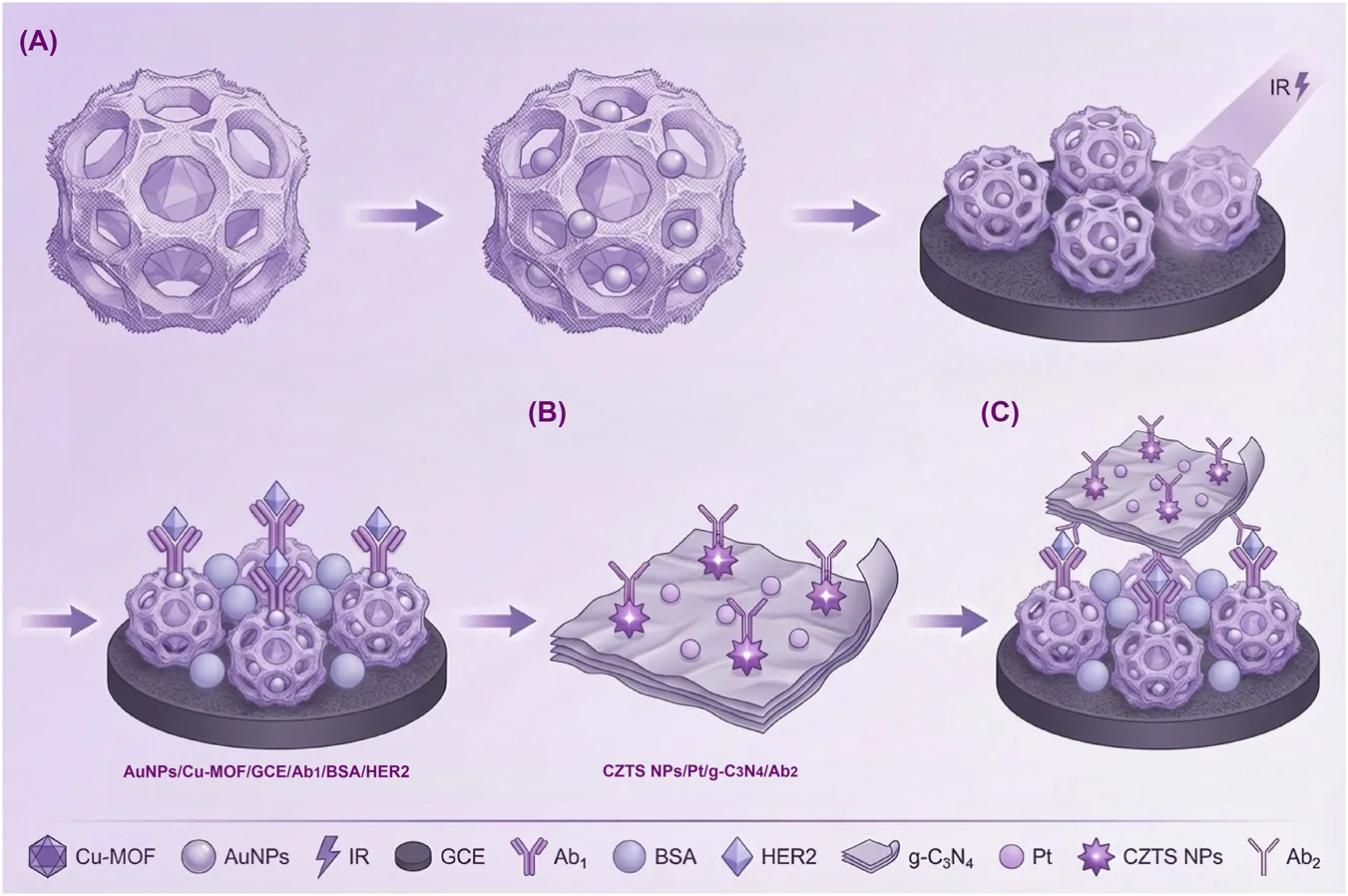
Schematic Diagram of HER2 Electrochemical Immunosensor Based on AuNPs/Cu-MOF and CZTS NPs/Pt/g-C_3_N_4_. **(A)** Development of the Immunosensing Platform: Amine-derived AuNPs/Cu-MOF composite was synthesized via amidation reaction, followed by GCE modification and covalent Ab_1_ immobilization through amine coupling. **(B)** Fabrication of the Immune Probe (Signal Amplifier): Sequential preparation of CZTS/Pt/g-C_3_N_4_ nanocomposite via Pt doping and CZTS integration, conjugated with Ab_2_ through interaction for Z-scheme heterojunction-mediated signal amplification. **(C)** Immunosensing Recognition Process: Antigen-antibody binding induced charge redistribution in Z-scheme architecture, with sulfur vacancy defects enhancing carrier separation efficiency and Pt’s plasmonic effect amplifying photoelectrochemical response. Adapted from Ref (Yola, 2021). with modifications.

Copper zinc tin sulfide (Cu_2_ZnSnS_4_, CZTS), a catalytic metal chalcogenide with excellent optoelectronic properties, has attracted interest in sensor applications (Badkoobehhezaveh et al., 2018). Metal nanoparticle-modified g-C_3_N_4_, including Au- and Pt-based composites, also improves catalytic activity and electron-transfer kinetics (Devaraji and Gopinath, 2018; Ong et al., 2016; Zhang et al., 2019). Based on these advantages, Mehmet Lütfi Yola et al. (Yola, 2021) developed an HER2 electrochemical immunosensor with a detection limit of 3.0 fg mL^-1^. The sensor was fabricated by modifying a GCE with amino-functionalized AuNPs/Cu-MOF for anti-HER2-Ab_1_ immobilization, while CZTS NPs/Pt-doped g-C_3_N_4_ (Pt/g-C_3_N_4_) conjugated with anti-HER2-Ab_2_ served as the signal amplifier. The Z-scheme heterostructure integrated CZTS, Pt, and g-C_3_N_4_, where sulfur vacancies in CZTS, Pt-mediated surface plasmon resonance, and the high surface area of layered g-C_3_N_4_ synergistically enhanced charge separation and signal amplification, as depicted in Figure 9.

Ehzari and Safari (2022) synthesized Fe_3_O_4_@TMU-24-AuNPs magnetic nanocomposite by integrating Fe_3_O_4_NPs, a metal-organic framework (MOF, TMU-24), and AuNPs, covalently immobilizing anti-HER2 Ab_1_ via amine coupling on the Au^3+^-ligand framework within the MOF—leveraging its high surface area and adjustable pores for stable antibody binding—while Pt-doped CdTe QDs served as electrochemical labels for Ab_2_, creating a sandwich-type immunosensor; the magnetism of Fe_3_O_4_ enabled rapid enrichment of low-concentration HER2, and Pt:CdTe QDs amplified signals through quantum dot electrochemiluminescence, achieving a 1.0 pg/mL to 100.0 ng/mL detection range with a 0.175 pg/mL limit of detection.

Xiaoying Pei’s experiments confirmed that AuNPs/MXene nanocomposites improve electron-transfer kinetics and antibody loading (Pei et al., 2024). Building on this strategy, Sakthivel et al. (2024) developed a sandwich-type HER2 immunosensor by modifying electrodes with AuNP-decorated Ti_3_C_2_T_x_ (Au/MXene) for anti-HER2 Ab_1_ immobilization. A nitrogen-doped graphene (NG)/MOF-derived copper-manganese-cobalt oxide (CuMnCoO_x_) composite labeled anti-HER2 Ab_2_, in which NG provided a high surface area and rapid signal conduction, while CuMnCoO_x_ contributed catalytic signal amplification. This integrated Au/MXene–NG/CuMnCoO_x_ platform enabled HER2 detection over 1.0 × 10^-4^–50.0 ng mL^-1^, with a detection limit of 0.757 pg mL^-1^.

### 8.2 Electrochemiluminescent immunosensors for HER2 detection

Wasi et al. (2024) optimized sensor fabrication by using a graphite sheet (GS) electrode as a stable, conductive substrate, doping carbon quantum dots with nitrogen (N-CQDs) to enhance surface functional groups for antibody binding, and modifying with BSA to stabilize the HER2 antibody; this design leverages N-CQDs’ abundant functional groups to amplify signal via increased antibody conjugation efficiency, while GS ensures robust electron transfer, collectively enabling a 0.1–1.0 ng mL^-1^ detection range and 4.8 pg mL^-1^ limit of detection—innovatively addressing CQD limitations through nitrogen doping and synergistic material integration for precise HER2 biomarker analysis (Table 17).

**TABLE 17:** List of applications for nanomaterial-based immunosensors in HER2 detection.

Nanomaterials	Biological sample type	Fabrication principles	Signal amplification principle	Linearity range	Detection limit	Assay time	Sample volume	Recovery	Storage stability	References
HNT/C@PdNPs	human serum	Electrochemistry	Synergistic effect in nanocomposites, Electrochemical catalytic amplification	0.03–9.0 ng mL^-1^	8.0 pg mL^-1^	∼0.5–1 h	NR	96.2%–103.5% (RSD ∼2.0%–4.5%)	Good stability; detailed storage data NR	Bi et al. (2023)
CZTS NPs/Pt/g-C_3_N_4_ and AuNPs/Cu-MOF	NR	Electrochemistry	Synergistic effect in nanocomposites, Electrochemical catalytic amplification	N/A	3.0 fg mL^-1^	∼1–1.5 h	NR	96.5%–103.7% (RSD ∼2.0%–4.5%)	Good stability; detailed storage data NR	Yola (2021)
N-CQDs/GS	NR	Electrochemiluminescence	Synergistic effect in nanocomposites, Nitrogen doping effect-electron transfer synergistic effect	0.1–1.0 ng mL^-1^	4.8 pg mL^-1^	∼1 h	NR	96.5%–103.6% (RSD ∼2.0%–4.5%)	Good stability; detailed storage data NR	Amir et al. (2024)
Fe_3_O_4_@TMU-24-AuNPs and Pt:CdTe QDs	human serum	Electrochemistry	Synergistic effect in nanocomposites, Electrochemical catalytic amplification	1.0 pg mL^-1^–100.0 ng mL^-1^	0.175 pg mL^-1^	∼1–1.5 h	NR	96.8%–103.9% (RSD ∼2.0%–4.5%)	Good stability; detailed storage data NR	Ehzari and Safari (2022)
Au/MXene and NG/CuMnCoOx	NR	Electrochemistry	Synergistic effect in nanocomposites, Electrochemical catalytic amplification	1.0 × 10^-4^–50.0 ng mL^-1^	0.757 pg mL^-1^	∼1–1.5 h	NR	96.7%–103.8% (RSD ∼2.0%–4.5%)	Good stability; detailed storage data NR	Sakthivel et al. (2024)

Data compiled by the authors based on References; NR, not reported.

### 8.3 Comparative analysis of nanomaterial contributions in HER2 sensing

To further clarify the mechanism-oriented classification of HER2 immunosensors, representative platforms are summarized in Table 18. This table compares the reported systems according to electrochemical and electrochemiluminescent signal transduction strategies rather than treating nanoparticles, quantum dots, MXene, graphene, MOFs, and related composites as equivalent material categories. In electrochemical HER2 immunosensors, nanomaterials mainly enhance antibody immobilization, interfacial electron transfer, catalytic redox amplification, magnetic enrichment, and sandwich-type signal transduction. In electrochemiluminescent HER2 immunosensors, nanomaterials contribute to luminescence generation, conductive interface construction, and electron-transfer-assisted signal enhancement. This comparison provides a clearer understanding of how nanomaterials improve HER2 detection performance within specific sensing mechanisms.

**TABLE 18:** Comparative analysis of nanomaterial contributions in HER2 sensing.

Sensing strategy	Representative systems	Functional role of nanomaterials	Main amplification mechanism	Analytical advantage	References
Electrochemistry	HNT/C@PdNPs	HNT provides a stable biological interface; the carbon layer enhances conductivity; PdNPs enhance catalytic activity	Enhanced electron transfer + electrocatalytic amplification	Overcomes the insufficient conductivity of HNT	Bi et al. (2023)
Electrochemistry	AuNPs/Cu-MOF and CZTS NPs/Pt/g-C_3_N_4_	Cu-MOF provides a porous antibody immobilization interface; AuNPs promote electron transfer; CZTS/Pt/g-C_3_N_4_ acts as a signal probe	Sandwich structure + multicomponent catalytic amplification	Extremely low detection limit, reaching the fg·mL^-1^ level	Yola (2021)
Electrochemistry	Fe_3_O_4_@TMU-24-AuNPs and Pt:CdTe QDs	Fe_3_O_4_ provides a magnetic interface; MOF improves loading capacity; AuNPs enhance conductivity; QDs amplify the signal	Magnetic enrichment + electrochemical signal amplification	High sensitivity and stable interface	Ehzari and Safari (2022)
Electrochemistry	Au/MXene and NG/CuMnCoO_x_	MXene enhances charge transport; AuNPs immobilize antibodies; NG improves conductivity and catalysis; CuMnCoO_x_ provides catalytic signals	Enhanced electron transfer + catalytic amplification	Wide linear range and stable sandwich structure	Sakthivel et al. (2024)
Electrochemiluminescence	N-CQDs/GS	N-CQDs provide ECL emission; GS enhances conductivity and loading capacity	Nitrogen-doping effect + electron-transfer synergy	Low background and highly sensitive detection	Amir et al. (2024)

Data compiled by the authors based on References.

## 9 Emerging nanotechnology-enabled detection technologies for tumor marker immunosensing

Although nanomaterials have greatly improved the sensitivity and signal amplification capability of immunosensors, the clinical translation of tumor marker detection also depends on the integration of advanced sensing technologies. Recent progress indicates that nanomaterials are increasingly being combined with microfluidic platforms, artificial intelligence-assisted diagnosis, digital biosensing, single-molecule detection, multiplexed detection systems, and wearable or portable devices. These emerging strategies extend the role of nanomaterials from signal-enhancing components to key elements of integrated diagnostic systems.

### 9.1 Microfluidic nanobiosensing platforms

Microfluidic platforms provide miniaturized, low-volume, and highly integrated systems for biomarker detection. When combined with nanomaterial-enhanced immunosensors, microfluidic chips can improve sample handling, accelerate antigen-antibody reactions, reduce reagent consumption, and enable automated detection. Nanomaterials such as AuNPs, quantum dots, graphene derivatives, magnetic nanoparticles, and MXenes can be integrated into microchannels as signal probes, capture substrates, or catalytic amplifiers (Kumari et al., 2024; Liu et al., 2024). For tumor marker detection, microfluidic nanobiosensors are particularly attractive because they can process small amounts of serum, plasma, saliva, or other body fluids while maintaining high sensitivity and rapid response. Therefore, microfluidics provides an important route for converting conventional laboratory immunoassays into point-of-care diagnostic platforms.

### 9.2 Artificial intelligence-assisted diagnosis and signal interpretation

Artificial intelligence has become an important tool for improving biosensor performance and diagnostic interpretation. In nanomaterial-enhanced immunosensors, artificial intelligence and machine learning algorithms can be used for signal denoising, feature extraction, calibration curve optimization, pattern recognition, and multi-biomarker data interpretation. This is especially valuable for complex sensing outputs generated by electrochemical, photoelectrochemical, electrochemiluminescent, chemiluminescent, and optical platforms (Wasi et al., 2024). By integrating artificial intelligence with nanosensor arrays, weak signals from low-abundance tumor markers can be more accurately distinguished from background interference (Yin, 2025). In addition, artificial intelligence-assisted models may help combine biomarker levels with clinical information, imaging data, or longitudinal monitoring results, thereby improving diagnostic accuracy and risk stratification.

### 9.3 Digital biosensing and single-molecule detection

Digital biosensing and single-molecule detection represent important directions for ultrasensitive tumor marker analysis. Unlike conventional ensemble measurements, digital biosensing divides the sample into numerous isolated micro- or nano-compartments and converts molecular recognition events into countable digital signals. This strategy can reduce background noise and improve the detection of extremely low-abundance biomarkers. Nanomaterials can further enhance digital immunoassays by improving capture efficiency, fluorescence or electrochemical readout, magnetic separation, and signal stability (He et al., 2021). Single-molecule detection platforms, including bead-based digital immunoassays, nanopore sensing, and nanostructure-enhanced optical detection, are particularly useful for early cancer diagnosis, minimal residual disease monitoring, and recurrence surveillance, where biomarker concentrations may be far below the detection range of traditional assays (Wang et al., 2025; Zhang J. et al., 2024).

### 9.4 Multiplexed tumor marker detection systems

Because cancer diagnosis rarely depends on a single biomarker, multiplexed detection systems are increasingly important for improving diagnostic reliability. Nanomaterials provide multiple coding and signal amplification strategies for multiplexed immunosensing, including spatial coding, color or fluorescence coding, electrochemical peak coding, Raman tag coding, and magnetic or bead-based barcoding (Geka et al., 2023; Jarockyte et al., 2020; Li et al., 2025). These strategies allow simultaneous detection of multiple tumor markers, such as CEA, PSA, CA153, AFP, CA125, CA199, HER2, and other disease-related biomarkers. Multiplexed nanobiosensors can improve diagnostic specificity, reduce false-positive results, and provide more comprehensive information for cancer screening, prognosis evaluation, and treatment monitoring.

### 9.5 Wearable and portable sensing devices

Wearable and portable biosensing devices represent another promising direction for tumor marker monitoring. Compared with conventional laboratory assays, wearable or portable platforms offer advantages in real-time monitoring, non-invasive or minimally invasive sampling, patient-centered testing, and point-of-care applications (Kashaninejad et al., 2025; Wu KY. et al., 2025). Nanomaterials can improve the performance of wearable sensors by enhancing conductivity, flexibility, catalytic activity, anti-fouling properties, and signal stability (Singh et al., 2022). Although most wearable cancer biosensors are still at the proof-of-concept stage, their integration with flexible electronics, microfluidics, wireless data transmission, and artificial intelligence may enable continuous monitoring of cancer-related biomarkers in sweat, interstitial fluid, saliva, or other accessible biofluids (Omar et al., 2024; Yuan et al., 2023).

## 10 Discussion

This review systematically summarizes recent advances in nanomaterial-enhanced immunosensors for tumor marker detection, with a focus on clinically relevant biomarkers including CEA, PSA, CA15-3, AFP, CA125, CA19-9, and HER2. In electrochemical, photoelectrochemical, electrochemiluminescence, and chemiluminescence platforms, nanomaterials such as AuNPs, quantum dots, carbon nanotubes, MXenes, and graphene-based composites have significantly improved sensitivity, signal amplification efficiency, and multiplexing capability. Most reported systems achieve detection limits in the fg–pg·mL^-1^ range, which is clearly superior to conventional ELISA-based methods. However, different sensing strategies show distinct advantages and limitations. Electrochemical immunosensors remain the most widely used due to their simplicity, low cost, and compatibility with miniaturization. However, they are often limited by matrix interference and baseline drift. Photoelectrochemical sensors provide higher signal-to-noise ratios through light-driven charge separation, but require complex material engineering and light source integration. Electrochemiluminescence systems show excellent sensitivity and a wide dynamic range, but rely on luminophore systems and co-reactants whose stability should be carefully controlled. Chemiluminescence sensors are simple in design, but often suffer from limited temporal stability and lower reproducibility. Therefore, no single platform is universally optimal. Performance depends on the balance between sensitivity, stability, and operational complexity.

The performance of nanomaterial-based immunosensors fundamentally depends on several physicochemical parameters: (i) high specific surface area for high-density antibody immobilization, (ii) tunable electronic structure for efficient charge transfer, (iii) catalytic activity for signal amplification, and (iv) surface chemistry for stable bioconjugation. In addition, structural engineering strategies such as heterojunction formation, core–shell design, and hybrid conductive networks significantly enhance electron transfer kinetics and reduce recombination losses. These design principles explain why many nanomaterial-enhanced platforms can achieve ultralow detection limits and broad linear ranges. Nevertheless, analytical sensitivity alone is insufficient to determine whether a sensing platform can be translated into routine clinical testing.

Despite excellent analytical performance at the laboratory scale, nanomaterial-enhanced immunosensors still face several key barriers to clinical translation. First, variability in nanomaterial synthesis leads to differences in structure and surface chemistry between independent batches, resulting in poor reproducibility and inconsistent sensor performance. This issue is particularly evident in different synthesis routes such as hydrothermal methods, sol–gel processes, and electrochemical deposition. Even small changes in nanointerface structure can significantly affect electron transfer kinetics and biomolecule immobilization efficiency. Second, although most studies demonstrate good performance in short-term serum or buffer systems, long-term stability in complex biological environments is still not well studied. In real physiological conditions, protein corona formation, ionic strength fluctuations, and enzymatic degradation continuously alter nanointerface structure and charge transfer behavior, leading to signal drift and reduced sensitivity. Third, biofouling and non-specific adsorption in real clinical samples significantly reduce sensing reliability. High-abundance serum proteins such as albumin, immunoglobulins, and fibrinogen can form competitive adsorption layers on nanointerfaces, masking active binding sites and increasing background signals. Although PEGylation, zwitterionic coatings, and biomimetic antifouling interfaces have been proposed, their long-term stability and scalability remain limited. Fourth, large-scale manufacturing and standardization remain major barriers to industrial translation. Multicomponent hybrid nanostructures usually involve multi-step synthesis and post-functionalization processes, which are difficult to control under good manufacturing practice conditions. In addition, a unified quality standard system for nanointerface structure–property relationships is still lacking. This issue has been widely emphasized in nanomedicine translation research.

A further important limitation is that many reported sensors are not sufficiently evaluated from the perspective of practical clinical relevance. In routine clinical laboratories, tumor markers are commonly quantified using commercially available automated immunoassay systems, including enzyme-linked immunosorbent assay, chemiluminescence immunoassay, electrochemiluminescence immunoassay, fluorescence-based immunoassay, and LOCI-based platforms. These established systems are widely accepted because they provide standardized calibration, high-throughput operation, quality control procedures, and clinically validated reference intervals. Therefore, emerging nanomaterial-enhanced immunosensors should not be assessed only by their limit of detection or linear range, but should also be benchmarked against these commercial diagnostic methods whenever possible.

In addition, the analytical range of a nanosensor should be interpreted in relation to clinically accepted decision thresholds. For example, commonly used clinical cutoffs include approximately 4.0 ng mL^-1^ for PSA, around 5 ng mL^-1^ for CEA, 25–35 U·mL^-1^ for CA125, 37 U·mL^-1^ for CA19-9, and 25–30 U·mL^-1^ for CA15-3, although these values may vary depending on patient populations, disease context, assay platform, and clinical guidelines. Therefore, an ultralow detection limit far below the clinical cutoff does not automatically indicate superior clinical utility. A clinically useful sensor should demonstrate stable accuracy, precision, recovery, and reproducibility near the decision-making concentration range. This point is particularly important for tumor markers such as CEA, PSA, AFP, CA15-3, CA125, CA19-9, and HER2, because their clinical application often depends on monitoring dynamic changes around clinically meaningful intervals rather than simply detecting trace concentrations in ideal buffer systems.

Real patient sample validation is also essential for clinical translation. Many nanomaterial-enhanced immunosensors are still validated only in buffer solutions, spiked serum, or a small number of serum samples. These conditions cannot fully reflect the complexity of real clinical matrices. Future studies should therefore include larger cohorts of real serum or plasma samples and directly compare the results with commercial diagnostic assays, such as enzyme-linked immunosorbent assay, chemiluminescence immunoassay, electrochemiluminescence immunoassay, or automated analyzer-based methods. Correlation coefficients, recovery rates, relative standard deviations, receiver operating characteristic analysis, and agreement analysis such as Bland–Altman plots should be reported to evaluate diagnostic reliability. Practical assay parameters, including assay time, sample volume, storage stability, inter-batch reproducibility, anti-interference ability, and operational simplicity, should also be systematically provided. These parameters are necessary to determine whether a proof-of-concept nanosensor can move toward routine clinical diagnostics.

Based on these challenges, future development of nanomaterial-enhanced immunosensors should focus on integrated optimization from material synthesis to interface engineering and system integration, in order to achieve true clinical translation. At the material level, improving reproducibility and structural consistency of nanomaterial synthesis remains a primary task. At the interface level, antibody immobilization should shift from empirical coupling to standardized surface chemistry design. This can be achieved by controlling functional group density, spatial orientation, and interfacial energy barriers, enabling more efficient and stable oriented immobilization while reducing activity loss caused by random adsorption. In this context, antifouling interface design is critical for improving reliability in complex biological samples. Biomimetic antifouling layers, zwitterionic polymers, and multiscale hydrated interfaces can effectively suppress protein corona formation and non-specific adsorption, thereby improving long-term stability and clinical applicability. Meanwhile, integration of multiplex detection capability is an important direction for next-generation biosensing systems. By constructing multi-channel signal transduction architectures or multi-probe cooperative recognition systems, simultaneous and dynamic detection of multiple tumor markers can be achieved, improving diagnostic accuracy and disease stratification. At the system level, artificial intelligence-assisted signal decoding is becoming an important trend. Machine learning algorithms can extract features from complex electrochemical or optical signals and improve both sensitivity and anti-interference capability. In addition, microfluidic integration enables automated sample processing, reaction, and detection, significantly reducing sample consumption and improving throughput. Wearable biosensors and smartphone-based readout systems provide new opportunities for real-time, non-invasive, and home-based health monitoring. Furthermore, rational nanomaterial design guided by theoretical modeling and machine learning can further optimize sensor performance.

Overall, nanomaterial-enhanced immunosensors provide a powerful strategy for ultrasensitive tumor marker detection and show great potential for early cancer diagnostics. However, future studies should move beyond reporting only ultralow detection limits and should place greater emphasis on clinically relevant concentration ranges, comparison with commercial diagnostic assays, validation using real patient samples, and standardized reporting of practical assay parameters. Only by combining high analytical sensitivity with reproducibility, stability, clinical benchmarking, and workflow compatibility can nanomaterial-enhanced immunosensors be translated from laboratory-scale platforms into clinically applicable diagnostic tools.

## References

[B1] Abelev G. I. Sell S. (1999). Tumor markers. Introduction. Semin. Cancer Biol. 9 (2), 61–65. 10.1006/scbi.1998.0088 10202128

[B2] Afzali D. Fayazi M. (2016). Deposition of MnO2 nanoparticles on the magnetic halloysite nanotubes by hydrothermal method for lead(II) removal from aqueous solutions. J. Taiwan Inst. Chem. Eng. 63, 421–429. 10.1016/j.jtice.2016.02.025

[B3] Ahn K. S. O'Brien D. R. Kim Y. H. Kim T. S. Yamada H. Park J. W. (2021). Associations of serum tumor biomarkers with integrated genomic and clinical characteristics of hepatocellular carcinoma. Liver Cancer 10 (6), 593–605. 10.1159/000516957 34950182 PMC8647136

[B4] Amani J. Khoshroo A. Rahimi-Nasrabadi M. (2017). Electrochemical immunosensor for the breast cancer marker CA 15-3 based on the catalytic activity of a CuS/reduced graphene oxide nanocomposite towards the electrooxidation of catechol. Mikrochim. Acta 185 (1), 79. 10.1007/s00604-017-2532-5 29594363

[B5] Amir H. Subramanian V. Sornambikai S. Ponpandian N. Viswanathan C. (2024). Nitrogen-enhanced carbon quantum dots mediated immunosensor for electrochemical detection of HER2 breast cancer biomarker. Bioelectrochemistry 155, 108589. 10.1016/j.bioelechem.2023.108589 37918312

[B6] Asbaghian-Namin H. Karami P. Naghshara H. Gholamin D. Johari-Ahar M. (2021). Electrochemiluminescent immunoassay for the determination of CA15-3 and CA72-4 using graphene oxide nanocomposite modified with CdSe quantum dots and Ru(bpy)(3) complex. Mikrochim. Acta 188 (7), 238. 10.1007/s00604-021-04890-2 34184115

[B7] Assari P. Rafati A. A. Feizollahi A. Joghani R. A. (2020). Fabrication of a sensitive label free electrochemical immunosensor for detection of prostate specific antigen using functionalized multi-walled carbon nanotubes/polyaniline/AuNPs. Mater Sci. Eng. C Mater Biol. Appl. 115, 111066. 10.1016/j.msec.2020.111066 32600691

[B8] Augustine S. Kumar P. Malhotra B. D. (2019). Amine-Functionalized MoO3@RGO nanohybrid-based biosensor for breast cancer detection. ACS Appl. Bio Mater. 2 (12), 5366–5378. 10.1021/acsabm.9b00659 35021536

[B9] Azzouzi S. Ben Ali M. Bellagambi F. Elaissari A. Jaffrezic-Renault N. Errachid A. (2022). Spatially hierarchical nano-architecture for real time detection of Interleukin-8 cancer biomarker. Talanta 246, 123436. 10.1016/j.talanta.2022.123436 35489096

[B10] Badkoobehhezaveh A. M. Abdizadeh H. Golobostanfard M. R. (2018). Electrophoretic behavior of solvothermal synthesized anion replaced Cu2ZnSn(SxSe1-x)4 films for photoelectrochemical water splitting. Int. J. Hydrogen Energy 43 (27), 11990–12001. 10.1016/j.ijhydene.2018.04.140

[B11] Bao Y. Han K. Ding Z. Li Y. Li T. Guan M. (2021). A label-free electrochemiluminescence immunosensor for carbohydrate antigen 153 based on polypyrrole-luminol-AuNPs nanocomposites with bi-catalysis. Spectrochim. Acta A Mol. Biomol. Spectrosc. 253, 119562. 10.1016/j.saa.2021.119562 33611216

[B12] Baumgardner W. J. Choi J. J. Lim Y.-F. Hanrath T. (2010). SnSe nanocrystals: synthesis, structure, optical properties, and surface chemistry. J. Am. Chem. Soc. 132 (28), 9519–9521. 10.1021/ja1013745 20578741

[B13] Beauchemin N. Arabzadeh A. (2013). Carcinoembryonic antigen-related cell adhesion molecules (CEACAMs) in cancer progression and metastasis. Cancer Metastasis Rev. 32 (3-4), 643–671. 10.1007/s10555-013-9444-6 23903773

[B14] Bi L. Teng Y. Baghayeri M. Bao J. (2023). Employing Pd nanoparticles decorated on halloysite nanotube/carbon composite for electrochemical aptasensing of HER2 in breast cancer patients. Environ. Res. 237 (Pt 2), 117030. 10.1016/j.envres.2023.117030 37659641

[B15] Biswas S. Lan Q. Xie Y. Sun X. Wang Y. (2021). Label-Free electrochemical immunosensor for ultrasensitive detection of carbohydrate antigen 125 based on antibody-immobilized biocompatible MOF-808/CNT. ACS Appl. Mater Interfaces 13 (2), 3295–3302. 10.1021/acsami.0c14946 33400479

[B16] Bölükbaşi Ö. S. Yola B. B. Karaman C. Atar N. Yola M. L. (2022). Electrochemical α-fetoprotein immunosensor based on Fe(3)O(4)NPs@covalent organic framework decorated gold nanoparticles and magnetic nanoparticles including SiO(2)@TiO(2). Mikrochim. Acta 189 (6), 242. 10.1007/s00604-022-05344-z 35654985

[B17] Bott-Neto J. L. Martins T. S. Buscaglia L. A. Machado S. A. S. Oliveira O. N. Jr (2022). Photocatalysis of TiO(2) sensitized with graphitic carbon nitride and electrodeposited aryl diazonium on screen-printed electrodes to detect prostate specific antigen under visible light. ACS Appl. Mater Interfaces 14 (19), 22114–22121. 10.1021/acsami.2c03106 35324137

[B18] Bray F. Laversanne M. Sung H. Ferlay J. Siegel R. L. Soerjomataram I. (2024). Global cancer statistics 2022: GLOBOCAN estimates of incidence and mortality worldwide for 36 cancers in 185 countries. CA Cancer J. Clin. 74 (3), 229–263. 10.3322/caac.21834 38572751

[B19] Brunstein J. (2016). Cost-effectiveness considerations with molecular diagnostics in oncology. MLO Med. Lab. Obs. 48 (5), 30–31. 27326449

[B20] Bushira F. A. Wang P. Jin Y. (2022). High-Entropy oxide for highly efficient luminol–dissolved oxygen electrochemiluminescence and biosensing applications. Anal. Chem. 94 (6), 2958–2965. 10.1021/acs.analchem.1c05005 35099931

[B21] Cao K. Xia Y. Yao J. Han X. Lambert L. Zhang T. (2023). Large-scale pancreatic cancer detection via non-contrast CT and deep learning. Nat. Med. 29 (12), 3033–3043. 10.1038/s41591-023-02640-w 37985692 PMC10719100

[B22] Carvajal S. Fera S. N. Jones A. L. Baldo T. A. Mosa I. M. Rusling J. F. (2018). Disposable inkjet-printed electrochemical platform for detection of clinically relevant HER-2 breast cancer biomarker. Biosens. Bioelectron. 104, 158–162. 10.1016/j.bios.2018.01.003 29331430 PMC5794514

[B23] Charkhchi P. Cybulski C. Gronwald J. Wong F. O. Narod S. A. Akbari M. R. (2020). CA125 and ovarian cancer: a comprehensive review. Cancers (Basel) 12 (12), 3730. 10.3390/cancers12123730 33322519 PMC7763876

[B24] Chen L. Chen Y. Feng Y. L. Zhu Y. Wang L. Q. Hu S. (2020a). Tumor circulome in the liquid biopsies for digestive tract cancer diagnosis and prognosis. World J. Clin. Cases 8 (11), 2066–2080. 10.12998/wjcc.v8.i11.2066 32548136 PMC7281040

[B25] Chen Y. Deng W. Tan Y. Xie Q. (2020b). CdS quantum-dots-decorated V(2)O(5) nanosheets as chemically etchable active materials for sensitive photoelectrochemical immunoassay of carcinoembryonic antigen. ACS Appl. Mater Interfaces 12 (26), 29066–29073. 10.1021/acsami.0c06793 32510918

[B26] Chen Z. Li B. Liu J. Li H. Li C. Xuan X. (2022). A label-free electrochemical immunosensor based on a gold-vertical graphene/TiO(2) nanotube electrode for CA125 detection in oxidation/reduction dual channels. Mikrochim. Acta 189 (7), 257. 10.1007/s00604-022-05332-3 35701556

[B27] Chen Z. Li H. Chen Z. Xuan X. Zhou B. Li M. (2023). Two-channel electrochemical immunosensor based on one-step-synthesized AuPt-boron-doped graphene electrode for CA153 detection. Biosens. Bioelectron. 222, 114974. 10.1016/j.bios.2022.114974 36495718

[B28] Cotchim S. Kongkaew S. Thavarungkul P. Kanatharana P. Limbut W. (2024). A dual-electrode label-free immunosensor based on *in situ* prepared Au-MoO(3)-Chi/porous graphene nanoparticles for point-of-care detection of cholangiocarcinoma. Talanta. 272, 125755. 10.1016/j.talanta.2024.125755 38364561

[B29] Cui R. Pan H. C. Zhu J. J. Chen H. Y. (2007). Versatile immunosensor using CdTe quantum dots as electrochemical and fluorescent labels. Anal. Chem. 79 (22), 8494–8501. 10.1021/ac070923d 17927140

[B30] Dai Y. Chiu L. Y. Chen Y. Qin S. Wu X. Liu C. C. (2019). Neutral charged immunosensor platform for protein-based biomarker analysis with enhanced sensitivity. ACS Sens. 4 (1), 161–169. 10.1021/acssensors.8b01126 30582808

[B31] Deng L. Lai G. Fu L. Lin C. T. Yu A. (2020). Enzymatic deposition of gold nanoparticles at vertically aligned carbon nanotubes for electrochemical stripping analysis and ultrasensitive immunosensing of carcinoembryonic antigen. Analyst 145 (8), 3073–3080. 10.1039/c9an02633a 32142088

[B32] Devaraji P. Gopinath C. S. (2018). Pt – g-C3N4 – (Au/TiO2): electronically integrated nanocomposite for solar hydrogen generation. Int. J. Hydrogen Energy 43 (2), 601–613. 10.1016/j.ijhydene.2017.11.057

[B33] Dou R. Du Z. Bao T. Dong X. Zheng X. Yu M. (2016). The polyvinylpyrrolidone functionalized rGO/Bi2S3 nanocomposite as a near-infrared light-responsive nanovehicle for chemo-photothermal therapy of cancer. Nanoscale 8 (22), 11531–11542. 10.1039/c6nr01543c 27203525

[B34] Ehzari H. Safari M. (2022). A sandwich-type electrochemical immunosensor using antibody-conjugated Pt-Doped CdTe QDs as enzyme-free labels for sensitive HER2 detection based on a magnetic framework. Front. Chem. 10, 881960. 10.3389/fchem.2022.881960 35755254 PMC9218600

[B35] Ehzari H. Amiri M. Safari M. (2020). Enzyme-free sandwich-type electrochemical immunosensor for highly sensitive prostate specific antigen based on conjugation of quantum dots and antibody on surface of modified glassy carbon electrode with core-shell magnetic metal-organic frameworks. Talanta. 210, 120641. 10.1016/j.talanta.2019.120641 31987217

[B36] El Harakeh M. Alawieh L. Saouma S. Halaoui L. I. (2009). Charge separation and photocurrent polarity-switching at CdS quantum dots assembly in polyelectrolyte interfaced with hole scavengers. Phys. Chem. Chem. Phys. 11 (28), 5962–5973. 10.1039/b820895f 19588019

[B37] El-Said W. A. Alshitari W. Choi J. W. (2020). Controlled fabrication of gold nanobipyramids/polypyrrole for shell-isolated nanoparticle-enhanced Raman spectroscopy to detect γ-aminobutyric acid. Spectrochim. Acta A Mol. Biomol. Spectrosc. 229, 117890. 10.1016/j.saa.2019.117890 31839573

[B38] Fan Z. Li Y. Yan K. Wu W. Yin S. Yang W. (2013). Application of contrast-enhanced ultrasound in the diagnosis of solid pancreatic lesions--a comparison of conventional ultrasound and contrast-enhanced CT. Eur. J. Radiol. 82 (9), 1385–1390. 10.1016/j.ejrad.2013.04.016 23727375

[B39] Fawzy A. Mohamed M. R. Ali M. A. Abd El-Magied M. H. Helal A. M. (2016). Tissue CA125 and HE4 gene expression levels offer superior accuracy in discriminating benign from malignant pelvic masses. Asian Pac J. Cancer Prev. 17 (1), 323–333. 10.7314/apjcp.2016.17.1.323 26838232

[B40] Fiebiger W. Wiltschke C. (2001). Tumor markers. Acta Med. Austriaca 28 (2), 33–37. 10.1046/j.1563-2571.2001.01008.x 11382139

[B41] Galle P. R. Foerster F. Kudo M. Chan S. L. Llovet J. M. Qin S. (2019). Biology and significance of alpha-fetoprotein in hepatocellular carcinoma. Liver Int. 39 (12), 2214–2229. 10.1111/liv.14223 31436873

[B42] Gangopadhyay B. Roy A. Paul D. Panda S. Das B. Karmakar S. (2024). 3-Polythiophene acetic acid nanosphere anchored few-layer graphene nanocomposites for label-free electrochemical immunosensing of liver cancer biomarker. ACS Appl. Bio Mater 7 (1), 485–497. 10.1021/acsabm.3c01126 38165836

[B43] Geka G. Kanioura A. Likodimos V. Gardelis S. Papanikolaou N. Kakabakos S. (2023). SERS immunosensors for cancer markers detection. Mater. (Basel) 16 (10), 3733. 10.3390/ma16103733 37241360 PMC10221005

[B44] Ghanei-Motlagh M. Taher M. A. (2018). A novel electrochemical sensor based on silver/halloysite nanotube/molybdenum disulfide nanocomposite for efficient nitrite sensing. Biosens. Bioelectron. 109, 279–285. 10.1016/j.bios.2018.02.057 29573727

[B45] Gharehaghaji Z. H. Khalilzadeh B. Yousefi H. Mohammad-Rezaei R. (2024). An electrochemical immunosensor based on MXene-GQD/AuNPs for the detection of trace amounts of CA-125 as specific tracer of ovarian cancer. Mikrochim. Acta 191 (7), 418. 10.1007/s00604-024-06469-z 38914884

[B46] Gholamin D. Karami P. Pahlavan Y. Johari-Ahar M. (2023). Highly sensitive photoelectrochemical immunosensor for detecting cancer marker CA19-9 based on a new SnSe quantum dot. Mikrochim. Acta 190 (4), 154. 10.1007/s00604-023-05718-x 36961600

[B47] Gillen S. Schuster T. Meyer Zum Büschenfelde C. Friess H. Kleeff J. (2010). Preoperative/neoadjuvant therapy in pancreatic cancer: a systematic review and meta-analysis of response and resection percentages. PLoS Med. 7 (4), e1000267. 10.1371/journal.pmed.1000267 20422030 PMC2857873

[B48] Hajdu S. I. Vadmal M. (2013). A note from history: landmarks in history of cancer, part 6. Cancer 119 (23), 4058–4082. 10.1002/cncr.28319 24105604

[B49] Han S. Yao A. Wang Y. (2021). An ionic liquid-molecularly imprinted composite based on graphene oxide for the specific recognition and extraction of cancer antigen 153. RSC Adv. 11 (22), 13085–13090. 10.1039/d1ra00134e 35423893 PMC8697238

[B50] Hao N. Lu J. Chi M. Xiong M. Zhang Y. Hua R. (2019). A universal photoelectrochemical biosensor for dual microRNA detection based on two CdTe nanocomposites. J. Mater Chem. B 7 (7), 1133–1141. 10.1039/c8tb03195a 32254781

[B51] He L. Tessier D. R. Briggs K. Tsangaris M. Charron M. McConnell E. M. (2021). Digital immunoassay for biomarker concentration quantification using solid-state nanopores. Nat. Commun. 12 (1), 5348. 10.1038/s41467-021-25566-8 34504071 PMC8429538

[B52] Henry N. L. Hayes D. F. (2006). Uses and abuses of tumor markers in the diagnosis, monitoring, and treatment of primary and metastatic breast cancer. Oncologist 11 (6), 541–552. 10.1634/theoncologist.11-6-541 16794234

[B53] Ho W. K. Hassan N. T. Yoon S. Y. Yang X. Lim J. M. C. Binte Ishak N. D. (2024). Age-specific breast and ovarian cancer risks associated with germline BRCA1 or BRCA2 pathogenic variants - an Asian study of 572 families. Lancet Reg. Health West Pac 44, 101017. 10.1016/j.lanwpc.2024.101017 38333895 PMC10851205

[B54] Hu L. Cui J. Wang Y. Jia J. (2023). An ultrasensitive electrochemical biosensor for bisphenol A based on aptamer-modified MrGO@AuNPs and ssDNA-functionalized AuNP@MBs synergistic amplification. Chemosphere 311 (Pt 2), 137154. 10.1016/j.chemosphere.2022.137154 36351468

[B55] Hughes D. M. Berhane S. Emily de Groot C. A. Toyoda H. Tada T. Kumada T. (2021). Serum levels of α-Fetoprotein increased more than 10 years before detection of hepatocellular carcinoma. Clin. Gastroenterol. Hepatol. 19 (1), 162. 10.1016/j.cgh.2020.04.084 32389887 PMC7611145

[B56] Jarockyte G. Karabanovas V. Rotomskis R. Mobasheri A. (2020). Multiplexed nanobiosensors: current trends in early diagnostics. Sensors (Basel) 20 (23), 6890. 10.3390/s20236890 33276535 PMC7729484

[B57] Kailemia M. J. Park D. Lebrilla C. B. (2017). Glycans and glycoproteins as specific biomarkers for cancer. Anal. Bioanal. Chem. 409 (2), 395–410. 10.1007/s00216-016-9880-6 27590322 PMC5203967

[B58] Kashaninejad N. De Saram P. Abdelfattah M. A. Bakare A. Vu H. H. (2025). Wearable biosensors for cancer detection and monitoring. Prog. Mol. Biol. Transl. Sci. 215, 311–354. 10.1016/bs.pmbts.2025.05.005 40683746

[B59] Kesavan G. Chen S. M. (2021). Manganese oxide anchored on carbon modified halloysite nanotubes: an electrochemical platform for the determination of chloramphenicol. Colloids Surfaces A Physicochem. Eng. Aspects 615, 126243. 10.1016/j.colsurfa.2021.126243

[B60] Kilimci U. Öndeş B. Sunna Ç. Uygun M. Aktaş Uygun D. (2025). Development of label-free immunosensors based on AuNPs-fullerene nanocomposites for the determination of cancer antigen 125. Bioelectrochemistry 163, 108863. 10.1016/j.bioelechem.2024.108863 39642770

[B61] Klein A. P. (2021). Pancreatic cancer epidemiology: understanding the role of lifestyle and inherited risk factors. Nat. Rev. Gastroenterol. Hepatol. 18 (7), 493–502. 10.1038/s41575-021-00457-x 34002083 PMC9265847

[B62] Kumari M. Gupta V. Kumar N. Arun R. K. (2024). Microfluidics-based nanobiosensors for healthcare monitoring. Mol. Biotechnol. 66 (3), 378–401. 10.1007/s12033-023-00760-9 37166577 PMC10173227

[B63] Lai Y. Wang L. Liu Y. Yang G. Tang C. Deng Y. (2018). Immunosensors based on nanomaterials for detection of tumor markers. J. Biomed. Nanotechnol. 14 (1), 44–65. 10.1166/jbn.2018.2505 29463365

[B64] Li M. M. Datto M. Duncavage E. J. Kulkarni S. Lindeman N. I. Roy S. (2017). Standards and Guidelines for the interpretation and reporting of sequence variants in cancer: a joint consensus recommendation of the Association for molecular pathology, American Society of clinical oncology, and college of American pathologists. J. Mol. Diagn 19 (1), 4–23. 10.1016/j.jmoldx.2016.10.002 27993330 PMC5707196

[B65] Li M. Jiang F. Xue L. Peng C. Shi Z. Zhang Z. (2022). Recent progress in biosensors for detection of tumor biomarkers. Molecules 27 (21), 7327. 10.3390/molecules27217327 36364157 PMC9658374

[B66] Li Z. Xu K. Qin L. Zhao D. Yang N. Wang D. (2023). Hollow nanomaterials in advanced drug delivery systems: from Single-to multiple shells. Adv. Mater 35 (12), e2203890. 10.1002/adma.202203890 35998336

[B67] Li J. Y. Zuo L. P. Xu J. Sun C. Y. (2024). Clinical applications of circulating tumor DNA in hematological malignancies: from past to the future. Blood Rev. 68, 101237. 10.1016/j.blre.2024.101237 39261219

[B68] Li Z. Guo M. Zhong W. (2025). Multiplex detection of biomarkers empowered by nanomaterials. Precis. Chem. 3 (6), 297–318. 10.1021/prechem.4c00096 40574998 PMC12188404

[B69] Lilja H. Ulmert D. Vickers A. J. (2008). Prostate-specific antigen and prostate cancer: prediction, detection and monitoring. Nat. Rev. Cancer 8 (4), 268–278. 10.1038/nrc2351 18337732

[B70] Lin L. P. Tham S. Y. Loh H. S. Tan M. T. T. (2021). Biocompatible graphene-zirconia nanocomposite as a cyto-safe immunosensor for the rapid detection of carcinoembryonic antigen. Sci. Rep. 11 (1), 22536. 10.1038/s41598-021-99498-0 34795382 PMC8602324

[B71] Lin Z. Zheng S. Xie J. Zhou R. Chen Y. Gao W. (2023). A sensitive electrochemiluminescence immunosensor for the detection of CA15-3 based on CeO(2)/Pt/rGO as a novel co-reaction accelerator. Talanta 253, 123912. 10.1016/j.talanta.2022.123912 36115102

[B72] Liu G. Guan X. Li B. Zhou H. Kong N. Wang H. (2022). Hemin-graphene oxide-gold nanoflower-assisted enhanced electrochemiluminescence immunosensor for determination of prostate-specific antigen. Mikrochim. Acta 189 (8), 297. 10.1007/s00604-022-05387-2 35900602

[B73] Liu Z. Zhou Y. Lu J. Gong T. Ibáñez E. Cifuentes A. (2024). Microfluidic biosensors for biomarker detection in body fluids: a key approach for early cancer diagnosis. Biomark. Res. 12 (1), 153. 10.1186/s40364-024-00697-4 39639411 PMC11622463

[B74] Ma H. Li X. Yan T. Li Y. Zhang Y. Wu D. (2016). Electrochemiluminescent immunosensing of prostate-specific antigen based on silver nanoparticles-doped Pb (II) metal-organic framework. Biosens. Bioelectron. 79, 379–385. 10.1016/j.bios.2015.12.080 26735872

[B75] Ma X. Fang C. Yan J. Zhao Q. Tu Y. (2018). A label-free electrochemiluminescent immunosensor for glutamate decarboxylase antibody detection on AuNPs supporting interface. Talanta. 186, 206–214. 10.1016/j.talanta.2018.04.052 29784351

[B76] Ma X. Tu X. Gao F. Xie Y. Huang X. Fernandez C. (2020). Hierarchical porous MXene/amino carbon nanotubes-based molecular imprinting sensor for highly sensitive and selective sensing of fisetin. Sensors Actuators B Chem. 309, 127815. 10.1016/j.snb.2020.127815

[B77] Ma X. Deng D. Xia N. Hao Y. Liu L. (2021). Electrochemical immunosensors with PQQ-Decorated carbon nanotubes as signal labels for electrocatalytic oxidation of Tris(2-carboxyethyl)phosphine. Nanomater. (Basel) 11 (7), 1757. 10.3390/nano11071757 PMC8308108 34361143

[B78] Malati T. (2007). Tumour markers: an overview. Indian J. Clin. biochem. 22 (2), 17–31. 10.1007/bf02913308 23105677 PMC3453798

[B79] Martins T. S. Bott-Neto J. L. Oliveira O. N. Jr. Machado S. A. S. (2021). A sandwich-type electrochemical immunosensor based on Au-rGO composite for CA15-3 tumor marker detection. Mikrochim. Acta 189 (1), 38. 10.1007/s00604-021-05145-w 34958417

[B80] Molina R. Barak V. van Dalen A. Duffy M. J. Einarsson R. Gion M. (2005). Tumor markers in breast cancer- European Group on Tumor Markers recommendations. Tumour Biol. 26 (6), 281–293. 10.1159/000089260 16254457

[B81] Nasrollahpour H. Khalilzadeh B. Naseri A. Mamaghani S. Isildak I. Rashidi M. R. (2023). Chitosan/luminol/AgNPs nanocomposite for electrochemiluminescent determination of prostate-specific antigen. Mikrochim. Acta 190 (3), 90. 10.1007/s00604-023-05680-8 36786882

[B82] Olorundare F. O. G. Sipuka D. S. Sebokolodi T. I. Kodama T. Arotiba O. A. Nkosi D. (2023). An electrochemical immunosensor for an alpha-fetoprotein cancer biomarker on a carbon black/palladium hybrid nanoparticles platform. Anal. Methods 15 (29), 3577–3585. 10.1039/d3ay00702b 37458385

[B83] Omar R. Saliba W. Khatib M. Zheng Y. Pieters C. Oved H. (2024). Biodegradable, biocompatible, and implantable multifunctional sensing platform for cardiac monitoring. ACS Sens. 9 (1), 126–138. 10.1021/acssensors.3c01755 38170944 PMC10825867

[B84] Ong W.-J. Tan L.-L. Ng Y. H. Yong S.-T. Chai S.-P. (2016). Graphitic carbon nitride (g-C3N4)-Based photocatalysts for artificial photosynthesis and environmental remediation: are we a step closer to achieving sustainability? Chem. Rev. 116 (12), 7159–7329. 10.1021/acs.chemrev.6b00075 27199146

[B85] Padmanabhan J. Kyriakides T. R. (2015). Nanomaterials, inflammation, and tissue engineering. Wiley Interdiscip. Rev. Nanomed Nanobiotechnol 7 (3), 355–370. 10.1002/wnan.1320 25421333 PMC4397128

[B86] Park H. S. Lee J. M. Choi H. K. Hong S. H. Han J. K. Choi B. I. (2009). Preoperative evaluation of pancreatic cancer: comparison of gadolinium-enhanced dynamic MRI with MR cholangiopancreatography versus MDCT. J. Magn. Reson Imaging 30 (3), 586–595. 10.1002/jmri.21889 19711405

[B87] Pathak A. Gangan A. S. Ratha S. Chakraborty B. Rout C. S. (2017). Enhanced pseudocapacitance of MoO3-Reduced graphene oxide hybrids with Insight from density functional theory investigations. J. Phys. Chem. C 121 (35), 18992–19001. 10.1021/acs.jpcc.7b04478

[B88] Pei X. Liu J. Zhang Y. Huang Y. Li Z. Niu X. (2024). Tetrahedral DNA-linked aptamer-antibody-based sandwich-type electrochemical sensor with Ag@Au core-shell nanoparticles as a signal amplifier for highly sensitive detection of α-fetoprotein. Mikrochim. Acta 191 (7), 414. 10.1007/s00604-024-06485-z 38904836

[B89] Qi F. Wu M. Liu S. Mu W. Wu C. Ren X. (2024). Ratiometric electrochemical immunosensor for the detection of CA199 based on the ratios of NiCo@Fc-MWCNTs-LDH and 3D-rGOF@Ag/Au complexes. Talanta 272, 125606. 10.1016/j.talanta.2023.125606 38394747

[B90] Qin D. Jiang X. Mo G. Feng J. Yu C. Deng B. (2019). A novel carbon quantum Dots signal amplification strategy coupled with sandwich Electrochemiluminescence immunosensor for the detection of CA15-3 in human serum. ACS Sens. 4 (2), 504–512. 10.1021/acssensors.8b01607 30693767

[B91] Qin X. Pan Y. Zhang J. Shen J. Li C. (2023). Ionic liquid functionalized trapezoidal Zn-MOF nanosheets integrated with gold nanoparticles for photoelectrochemical immunosensing alpha-fetoprotein. Talanta. 253, 123684. 10.1016/j.talanta.2022.123684 36126519

[B92] Qiu Z. Shu J. Tang D. (2017). Bioresponsive release System for visual fluorescence detection of carcinoembryonic antigen from mesoporous Silica nanocontainers mediated optical color on quantum dot-enzyme-impregnated paper. Anal. Chem. 89 (9), 5152–5160. 10.1021/acs.analchem.7b00989 28376620

[B93] Qiu R. Mu W. Wu C. Wu M. Feng J. Rong S. (2022). Sandwich-type immunosensor based on COF-LZU1 as the substrate platform and graphene framework supported nanosilver as probe for CA125 detection. J. Immunol. Methods. 504, 113261. 10.1016/j.jim.2022.113261 35351484

[B94] Ranjan P. Abubakar Sadique M. Yadav S. Khan R. Kumar Srivastava A. (2024). Electrochemical nanobiosensor of ionic liquid functionalized MoO(3)-rGO for sensitive detection of carcinoembryonic antigen. Chempluschem 89 (6), e202300625. 10.1002/cplu.202300625 38321835

[B95] Rao C. Shi S. (2022). Development of nanomaterials to target articular cartilage for osteoarthritis therapy. Front. Mol. Biosci. 9, 900344. 10.3389/fmolb.2022.900344 36032667 PMC9402910

[B96] Rawla P. Sunkara T. Gaduputi V. (2019). Epidemiology of pancreatic cancer: global trends, etiology and risk factors. World J. Oncol. 10 (1), 10–27. 10.14740/wjon1166 30834048 PMC6396775

[B97] Razmi H. Mohammad-Rezaei R. (2013). Graphene quantum dots as a new substrate for immobilization and direct electrochemistry of glucose oxidase: application to sensitive glucose determination. Biosens. Bioelectron. 41, 498–504. 10.1016/j.bios.2012.09.009 23098855

[B98] Ren Y. Zhou B. Yu M. Xue Y. Kong W. Yang R. (2025). Label-free differential chemiluminescent immunosensor based on magnetic nanoparticles CuFe(2)O(4)@ABEI-GNPs with dual catalytic sites. Anal. Chim. Acta 1333, 343397. 10.1016/j.aca.2024.343397 39615914

[B99] Riaz A. Ryu R. K. Kulik L. M. Mulcahy M. F. Lewandowski R. J. Minocha J. (2009). Alpha-fetoprotein response after locoregional therapy for hepatocellular carcinoma: oncologic marker of radiologic response, progression, and survival. J. Clin. Oncol. 27 (34), 5734–5742. 10.1200/JCO.2009.23.1282 19805671

[B100] Rong S. Zou L. Li Y. Guan Y. Guan H. Zhang Z. (2021). An ultrasensitive disposable sandwich-configuration electrochemical immunosensor based on OMC@AuNPs composites and AuPt-MB for alpha-fetoprotein detection. Bioelectrochemistry 141, 107846. 10.1016/j.bioelechem.2021.107846 34087545

[B101] Roskoski R. (2014). The ErbB/HER family of protein-tyrosine kinases and cancer. Pharmacol. Res. 79, 34–74. 10.1016/j.phrs.2013.11.002 24269963

[B102] Sakthivel R. Lin Y. C. Yu M. C. Dhawan U. Liu X. Chen J. C. (2024). A sensitive sandwich-type electrochemical immunosensor using nitrogen-doped graphene/metal-organic framework-derived CuMnCoO(x) and Au/MXene for the detection of breast cancer biomarker. Colloids Surf. B Biointerfaces 234, 113755. 10.1016/j.colsurfb.2024.113755 38241894

[B103] Seale K. N. Tkaczuk K. H. R. (2022). Circulating biomarkers in breast cancer. Clin. Breast Cancer 22 (3), e319–e331. 10.1016/j.clbc.2021.09.006 34756687

[B104] Sha Y. Guo Z. Chen B. Wang S. Ge G. Qiu B. (2015). A one-step electrochemiluminescence immunosensor preparation for ultrasensitive detection of carbohydrate antigen 19-9 based on multi-functionalized graphene oxide. Biosens. Bioelectron. 66, 468–473. 10.1016/j.bios.2014.12.013 25497987

[B105] Shang L. Zhao X. H. Zhang W. Jia L. P. Ma R. N. Xue Q. W. (2021). Graphene-PtPd nanocomposite for low-potential-driven electrochemiluminescent determination of carcinoembryonic antigen using Ru(bpy)(3)(2). Mikrochim. Acta 189 (1), 17. 10.1007/s00604-021-05120-5 34873664

[B106] Shanmugam M. Alsalme A. Alghamdi A. Jayavel R. (2015). Enhanced photocatalytic performance of the Graphene-V2O5 nanocomposite in the degradation of Methylene blue dye under direct sunlight. ACS Appl. Mater Interfaces 7 (27), 14905–14911. 10.1021/acsami.5b02715 26098875

[B107] Shen J. Situ B. Du X. Wang Z. Hu R. Li B. (2022). Aggregation-Induced emission luminogen-based dual-mode enzyme-linked immunosorbent assay for ultrasensitive detection of cancer biomarkers in a broad concentration range. ACS Sens. 7 (3), 766–774. 10.1021/acssensors.1c02237 35179886

[B108] Shimada H. Noie T. Ohashi M. Oba K. Takahashi Y. (2014). Clinical significance of serum tumor markers for gastric cancer: a systematic review of literature by the Task Force of the Japanese Gastric Cancer Association. Gastric Cancer 17 (1), 26–33. 10.1007/s10120-013-0259-5 23572188

[B109] Siegel R. L. Giaquinto A. N. Jemal A. (2024). Cancer statistics, 2024. CA Cancer J. Clin. 74 (1), 12–49. 10.3322/caac.21820 38230766

[B110] Singh S. U. Chatterjee S. Lone S. A. Ho H. H. Kaswan K. Peringeth K. (2022). Advanced wearable biosensors for the detection of body fluids and exhaled breath by graphene. Mikrochim. Acta 189 (6), 236. 10.1007/s00604-022-05317-2 35633385 PMC9146825

[B111] Srivastava M. Nirala N. R. Srivastava S. K. Prakash R. (2018). A comparative Study of aptasensor Vs immunosensor for label-free PSA cancer detection on GQDs-AuNRs modified screen-printed electrodes. Sci. Rep. 8 (1), 1923. 10.1038/s41598-018-19733-z 29386538 PMC5792442

[B112] Stenman U. H. Leinonen J. Zhang W. M. Finne P. (1999). Prostate-specific antigen. Semin. Cancer Biol. 9 (2), 83–93. 10.1006/scbi.1998.0086 10202130

[B113] Tang Y. Qiao G. Xu E. Xuan Y. Liao M. Yin G. (2017). Biomarkers for early diagnosis, prognosis, prediction, and recurrence monitoring of non-small cell lung cancer. Onco Targets Ther. 10, 4527–4534. 10.2147/OTT.S142149 28979144 PMC5602468

[B114] Tang Y. Liu Y. Xia Y. Zhao F. Zeng B. (2023). Simultaneous detection of ovarian cancer-concerned HE4 and CA125 markers based on Cu single-atom-triggered CdS QDs and Eu MOF@Isoluminol ECL. Anal. Chem. 95 (10), 4795–4802. 10.1021/acs.analchem.3c00273 36867090

[B115] Tao C. Rouhi J. (2023). A biosensor based on graphene oxide nanocomposite for determination of carcinoembryonic antigen in colorectal cancer biomarker. Environ. Res. 238 (Pt 1), 117113. 10.1016/j.envres.2023.117113 37696325

[B116] Viswambari Devi R. Doble M. Verma R. S. (2015). Nanomaterials for early detection of cancer biomarker with special emphasis on gold nanoparticles in immunoassays/sensors. Biosens. Bioelectron. 68, 688–698. 10.1016/j.bios.2015.01.066 25660660

[B117] Wagle N. S. Nogueira L. Devasia T. P. Mariotto A. B. Yabroff K. R. Islami F. (2025). Cancer treatment and survivorship statistics, 2025. CA Cancer J. Clin. 75 (4), 308–340. 10.3322/caac.70011 40445120 PMC12223361

[B118] Wang Y. Su Y. R. Qiao L. Liu L. X. Su Q. Zhu C. Q. (2011). Synthesis of one-dimensional TiO2/V2O5 branched heterostructures and their visible light photocatalytic activity towards Rhodamine B. Nanotechnology 22 (22), 225702. 10.1088/0957-4484/22/22/225702 21454938

[B119] Wang X. Chen L. Su X. Ai S. (2013a). Electrochemical immunosensor with graphene quantum dots and apoferritin-encapsulated Cu nanoparticles double-assisted signal amplification for detection of avian leukosis virus subgroup J. Biosens. Bioelectron. 47, 171–177. 10.1016/j.bios.2013.03.021 23570680

[B120] Wang Z. Chen J. Q. Liu J. L. Qin X. G. Huang Y. (2013b). FDG-PET in diagnosis, staging and prognosis of pancreatic carcinoma: a meta-analysis. World J. Gastroenterol. 19 (29), 4808–4817. 10.3748/wjg.v19.i29.4808 23922481 PMC3732856

[B121] Wang Y. S. Ren S. F. Jiang W. Lu J. Q. Zhang X. Y. Li X. P. (2021). CA125-Tn ELISA assay improves specificity of pre-operative diagnosis of ovarian cancer among patients with elevated serum CA125 levels. Ann. Transl. Med. 9 (9), 788. 10.21037/atm-20-8053 34268401 PMC8246179

[B122] Wang J. Bei J. Guo X. Ding Y. Chen T. Lu B. (2022). Ultrasensitive photoelectrochemical immunosensor for carcinoembryonic antigen detection based on pillar[5]arene-functionalized Au nanoparticles and hollow PANI hybrid BiOBr heterojunction. Biosens. Bioelectron. 208, 114220. 10.1016/j.bios.2022.114220 35358775

[B123] Wang X. Che Y. Liu S. Ma B. Liu Y. Lei J. (2025). Single-Molecule liquid biopsy detects Low- and high-abundance protein markers simultaneously for pancreatic cancer diagnosis. Anal. Chem. 97 (15), 8385–8393. 10.1021/acs.analchem.4c07031 40194997

[B124] Wasilewski T. Kamysz W. Gębicki J. (2024). AI-Assisted detection of biomarkers by sensors and biosensors for early diagnosis and monitoring. Biosens. (Basel). 14 (7), 356. 10.3390/bios14070356 PMC11274923 39056632

[B125] Wu H. Zhang G. Yang X. (2023). Electrochemical immunosensor based on Fe(3)O(4)/MWCNTs-COOH/AuNPs nanocomposites for trace liver cancer marker alpha-fetoprotein detection. Talanta. 259, 124492. 10.1016/j.talanta.2023.124492 37011563

[B126] Wu S. Ma H. Chen X. Zhong W. Gu Y. Miao Y. (2025a). Collard-like Bi(2)S(3)@Au nanocomposites-based label free electrochemical immunosensor for quantitative detection of CA19-9. Talanta 285, 127299. 10.1016/j.talanta.2024.127299 39671994

[B127] Wu K. Y. Su M. E. Kim Y. Nguyen L. Marchand M. Tran S. D. (2025b). Wearable biosensors: a comprehensive overview. Prog. Mol. Biol. Transl. Sci. 215, 101–154. 10.1016/bs.pmbts.2025.05.011 40683741

[B128] Xing Y. Lan X. Liu X. Huang Y. (2024). Nanomaterial-Mediated immunosensors and their performance in detecting tumor markers. Discov. Med. 36 (186), 1316–1333. 10.24976/discov.med.202436186.122 39054703

[B129] Xu Z. Zhou Y. Li M. Guo Z. Zheng X. (2023). A carbonate-involved amplification strategy for cathodic electrochemiluminescence of Luminol triggered by the catalase-like CoO nanorods. Anal. Chem. 95 (27), 10457–10463. 10.1021/acs.analchem.3c02066 37385957

[B130] Yan K. Liu Y. Yang Y. Zhang J. (2015). A cathodic “Signal-off” photoelectrochemical aptasensor for ultrasensitive and selective detection of oxytetracycline. Anal. Chem. 87 (24), 12215–12220. 10.1021/acs.analchem.5b03139 26551579

[B131] Yan L. Zhang C. Xi F. (2022). Disposable amperometric label-free immunosensor on chitosan-graphene-modified patterned ITO electrodes for prostate specific antigen. Molecules 27 (18), 5895. 10.3390/molecules27185895 36144631 PMC9505937

[B132] Yang H. Bright J. Kasani S. Zheng P. Musho T. Chen B. (2019). Metal–organic framework coated titanium dioxide nanorod array p–n heterojunction photoanode for solar water-splitting. Nano Res. 12 (3), 643–650. 10.1007/s12274-019-2272-4

[B133] Yang M. Wang L. Xie C. Lu H. Wang J. Li Y. (2025). A disposable ultrasensitive immunosensor based on MXene/NH(2)-CNT modified screen-printed electrode for the detection of ovarian cancer antigen CA125. Talanta 281, 126893. 10.1016/j.talanta.2024.126893 39288586

[B134] Yin S. (2025). Artificial intelligence-assisted nanosensors for clinical diagnostics: current advances and future prospects. Biosens. (Basel). 15 (10), 656. 10.3390/bios15100656 PMC12564406 41149307

[B135] Yola M. L. (2021). Sensitive sandwich-type voltammetric immunosensor for breast cancer biomarker HER2 detection based on gold nanoparticles decorated Cu-MOF and Cu(2)ZnSnS(4) NPs/Pt/g-C(3)N(4) composite. Mikrochim. Acta 188 (3), 78. 10.1007/s00604-021-04735-y 33569679

[B136] Yoon J. Shin M. Lim J. Lee J. Y. Choi J. W. (2020). Recent advances in MXene nanocomposite-based biosensors. Biosens. (Basel). 10 (11), 185. 10.3390/bios10110185 33233574 PMC7699737

[B137] Yuan X. Li C. Yin X. Yang Y. Ji B. Niu Y. (2023). Epidermal wearable biosensors for monitoring biomarkers of chronic disease in sweat. Biosens. (Basel). 13 (3), 313. 10.3390/bios13030313 36979525 PMC10045998

[B138] Zaak D. Kriegmair M. Stepp H. Stepp H. Baumgartner R. Oberneder R. (2001). Endoscopic detection of transitional cell carcinoma with 5-aminolevulinic acid: results of 1012 fluorescence endoscopies. Urology 57 (4), 690–694. 10.1016/s0090-4295(00)01053-0 11306382

[B139] Zeng X. Ma S. Bao J. Tu W. Dai Z. (2013). Using graphene-based plasmonic nanocomposites to quench energy from Quantum dots for Signal-On Photoelectrochemical aptasensing. Anal. Chem. 85 (24), 11720–11724. 10.1021/ac403408y 24256069

[B140] Zhang Y. Chai C. Zhang X. Liu J. Duan D. Fan C. (2019). Construction of Pt-decorated g-C3N4/Bi2WO6 Z-scheme composite with superior solar photocatalytic activity toward rhodamine B degradation. Inorg. Chem. Commun. 100, 81–91. 10.1016/j.inoche.2018.12.019

[B141] Zhang M. Cheng S. Jin Y. Zhao Y. Wang Y. (2021). Roles of CA125 in diagnosis, prediction, and oncogenesis of ovarian cancer. Biochim. Biophys. Acta Rev. Cancer 1875 (2), 188503. 10.1016/j.bbcan.2021.188503 33421585

[B142] Zhang G. Zhang H. Fu Y. Xia P. Chen C. Wei Y. (2024a). Construction of electrochemical immunosensor from MXene/multi-walled carbon nanotubes/gold nanoparticles for specific detection of carcinoembryonic antigen. Mikrochim. Acta 191 (10), 626. 10.1007/s00604-024-06706-5 39325066

[B143] Zhang S. Chen X. Hu S. Cai K. Peng C. Luo L. (2024b). Electrochemical immunosensor based on PtNPs/MoS(2)@rGO composite for the detection of alpha-fetoprotein in human serum. Mikrochim. Acta 191 (11), 662. 10.1007/s00604-024-06712-7 39387898

[B144] Zhang J. Liu J. Qiao L. Zhang Q. Hu J. Zhang C. Y. (2024c). Recent advance in single-molecule fluorescent biosensors for tumor biomarker detection. Biosens. (Basel). 14 (11), 540. 10.3390/bios14110540 39589999 PMC11591580

[B145] Zheng D. Pei S. Geng S. Zhang L. (2019). Synthesis of Fe-Fe3O4/rGO composite with heterostructure through room-temperature reduction as photo-fenton catalyst. J. Nanosci. Nanotechnol. 19 (9), 5858–5863. 10.1166/jnn.2019.16513 30961750

[B146] Zhong Y. Xu F. Wu J. Schubert J. Li M. M. (2021a). Application of next generation sequencing in laboratory medicine. Ann. Lab. Med. 41 (1), 25–43. 10.3343/alm.2021.41.1.25 32829577 PMC7443516

[B147] Zhong X. Zhang M. Guo L. Xie Y. Luo R. Chen W. (2021b). A dual-signal self-checking photoelectrochemical immunosensor based on the sole composite of MIL-101(Cr) and CdSe quantum dots for the detection of α-fetoprotein. Biosens. Bioelectron. 189, 113389. 10.1016/j.bios.2021.113389 34091283

[B148] Zhou Y. Zhang C. Liu J. Mou Y. (2025). Nanochannel confined graphene quantum dots/platinum nanoparticles boosts electrochemiluminescence of luminal-O(2) system for sensitive immunoassay. Talanta 285, 127223. 10.1016/j.talanta.2024.127223 39613487

[B149] Zhu X. Shan J. Dai L. Shi F. Wang J. Wang H. (2023). PB@PDA nanocomposites as nanolabels and signal reporters for separate-type cathodic photoelectrochemical immunosensors in the detection of carcinoembryonic antigens. Talanta. 254, 124134. 10.1016/j.talanta.2022.124134 36450179

